# Role of N6‐methyladenosine RNA modification in cancer

**DOI:** 10.1002/mco2.715

**Published:** 2024-09-09

**Authors:** Yi Qu, Nannan Gao, Shengwei Zhang, Limin Gao, Bing He, Chao Wang, Chunli Gong, Qiuyue Shi, Zhibin Li, Shiming Yang, Yufeng Xiao

**Affiliations:** ^1^ Department of Gastroenterology Xinqiao Hospital Army Medical University Chongqing China; ^2^ Department of Gastroenterology the First Affiliated Hospital of Guangxi Medical University Nanning Guangxi China

**Keywords:** cancer, immunity, m6A, microorganism, posttranslational modification, programmed cell death

## Abstract

N6‐methyladenosine (m6A) is the most abundant modification of RNA in eukaryotic cells. Previous studies have shown that m6A is pivotal in diverse diseases especially cancer. m6A corelates with the initiation, progression, resistance, invasion, and metastasis of cancer. However, despite these insights, a comprehensive understanding of its specific roles and mechanisms within the complex landscape of cancer is still elusive. This review begins by outlining the key regulatory proteins of m6A modification and their posttranslational modifications (PTMs), as well as the role in chromatin accessibility and transcriptional activity within cancer cells. Additionally, it highlights that m6A modifications impact cancer progression by modulating programmed cell death mechanisms and affecting the tumor microenvironment through various cancer‐associated immune cells. Furthermore, the review discusses how microorganisms can induce enduring epigenetic changes and oncogenic effect in microorganism‐associated cancers by altering m6A modifications. Last, it delves into the role of m6A modification in cancer immunotherapy, encompassing RNA therapy, immune checkpoint blockade, cytokine therapy, adoptive cell transfer therapy, and direct targeting of m6A regulators. Overall, this review clarifies the multifaceted role of m6A modification in cancer and explores targeted therapies aimed at manipulating m6A modification, aiming to advance cancer research and improve patient outcomes.

## Graphical abstract

N6‐methyladenosine (m6A) is the most abundant modification of RNA in eukaryotic cells. Previous studies have shown that m6A is pivotal in diverse diseases especially cancer. m6A corelates with the initiation, progression, resistance, invasion and metastasis of cancer. However, despite these insights, a comprehensive understanding of its specific roles and mechanisms within the complex landscape of cancer is still elusive. This review begins by outlining the key regulatory proteins of m6A modification and their posttranslational modifications, as well as the role in chromatin accessibility and transcriptional activity within cancer cells. Additionally, it highlights that m6A modifications impact cancer progression by modulating programmed cell death (PCD) mechanisms and affecting the tumor microenvironment through various cancer‐associated immune cells. Furthermore, the review discusses how microorganisms can induce enduring epigenetic changes and oncogenic effect in microorganism‐associated cancers by altering m6A modifications. Last, it delves into the role of m6A modification in cancer immunotherapy, encompassing RNA therapy, immune checkpoint blockade, cytokine therapy, adoptive cell transfer therapy, and direct targeting of m6A regulators. Overall, this review clarifies the multifaceted role of m6A modification in cancer and explores targeted therapies aimed at manipulating m6A modification, aiming to advance cancer research and improve patient outcomes

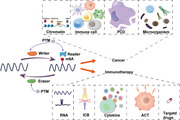

## 1 INTRODUCTION

Cancer is the second leading cause of death and imposes tremendous personal and societal burdens.^1^ Development, invasion, and metastasis of cancer involve an imbalance between cancer and immune cells. This imbalance is related to multiple factors, including exogenous (environmental pollution, chronic irritation, poisons or drugs, microbial action, and personal habits) and endogenous (genetic, epigenetic, and endocrine factors). Epigenetic processes include DNA methylation, histone modification, chromatin remodeling, noncoding RNA (ncRNA) and RNA modification.^2^, ^3^ Among the various RNA modification mechanisms, N6‐methyladenosine (m6A), first discovered in mammalian cells in 1974, is the most abundant.^4^ m6A, a dynamic and reversible RNA modification, is regulated by various proteins, including writers (methyltransferases), erasers (demethylases), and readers (m6A‐binding proteins). Previous studies have extensively investigated these proteins. However, recent research has increasingly focused on the posttranslational modifications (PTMs) of these regulatory proteins, which can significantly impact their activity and function. Exploring PTMs of m6A regulators represents a promising avenue for future research. m6A modification plays critical roles in RNA splicing, translation, stability, degradation, and translocation^5^ and can also reverse regulate chromatin status,^6^ ultimately influencing a range of human diseases, including cancer^7^ and other diseases such as cardiovascular diseases,^8^ autoimmune diseases,^9^ central nervous system diseases,^10^ reproductive system diseases,^11^ and metabolic diseases.^12^ Therefore, elucidating the role of m6A modifications in cancer may provide better targets for treatment.

Programmed cell death (PCD) represents a highly orchestrated form of cellular demise and is among the processes regulated by m6A modifications, impacting the interplay between cancer and immune cells. Recent studies indicate that m6A RNA modification can govern tumor‐associated immune cells, encompassing macrophages, neutrophils, dendritic cells (DCs), T cells, B cells, and natural killer (NK) cells. m6A modification crucially influences the growth, polarization, activation, and differentiation of these immune cells within the tumor microenvironment (TME). Moreover, m6A may be pivotal in anticancer immunity, particularly in cancers associated with specific microorganisms. The modification regulates the life cycle of several pathogens, and certain pathogens can produce oncogenic proteins that promote carcinogenesis by altering m6A methylation levels in the human body. Thus, targeting m6A modification in microorganisms presents a promising avenue for future cancer therapies.^13^

Various cancer treatments have been rapidly developed and widely used, including surgery,^14^ chemotherapy,^15^ radiotherapy,^16^ targeted therapy,^17^ endocrine therapy,^18^ and immunotherapy.^18^ In contrast to other therapies, immunotherapy focuses on the interactions between immune and cancer cells.^19^ Immunotherapy consists of gene therapy, immune checkpoint blockade (ICB), cytokine therapy, adoptive cell transfer (ACT) treatment,^20^ and direct targeting of m6A regulators. Although this strategy has achieved a degree of return, some patients still do not benefit because of multiple factors, such as immune evasion^21^ and drug resistance.^22^ However, the underlying mechanisms have not been fully elucidated. Therefore, we reviewed recent studies on m6A‐based immunotherapies. Targeting m6A modifications may shed new light on improving immunotherapy.

In this review, we first provide a comprehensive summary of the regulatory proteins involved in m6A modification, encompassing the writers (methyltransferases), erasers (demethylases), and readers (m6A‐binding proteins). Additionally, we elucidate their PTMs, including methylation, acetylation, lactylation, ubiquitination, SUMOylation, phosphorylation, and O‐GlcNAcylation. PTMs play a pivotal role in regulating the activity or stability of m6A regulatory proteins and impact their functions in cancer cells. Subsequently, we delve into the interplay between m6A modification and chromatin accessibility within cancer cells. Furthermore, our focus lies on recent advancements in understanding the implications of m6A modification in cancer from three distinct perspectives. First, m6A modifications are intricately involved in various PCD mechanisms such as autophagy, ferroptosis, pyroptosis, cuproptosis, and disulfidoptosis, which exhibit dual roles in cancer processes. Second, m6A modification exerts influence on the TME by modulating proliferation, polarization, recruitment, and activity of diverse immune cells including macrophages, neutrophils, DCs, T cells, B cells, and NK cells. Last, in certain microorganism‐associated cancers, m6A could potentially affect pathogen life cycles while specific oncogenic proteins derived from cancer‐related microorganisms can alter human m6Amethylation patterns as well. We summarize the roles played by *Helicobacter pylori* (Hp), *Fusobacterium nucleatum* (Fn), hepatitis B virus, Epstein‐Barr virus (EBV), and Human papillomavirus (HPV). At the end, we highlight how m6A modification plays an important role in cancer immunotherapy including RNA therapy, ICB, cytokine therapy, ACT, and direct targeting treatment of m6A regulatory proteins. A deeper investigation into the mechanisms underlying m6A may shed new light on future cancer research and treatment.

## 2 REGULATORY PROTEINS OF M6A MODIFICATION

**FIGURE 1:**
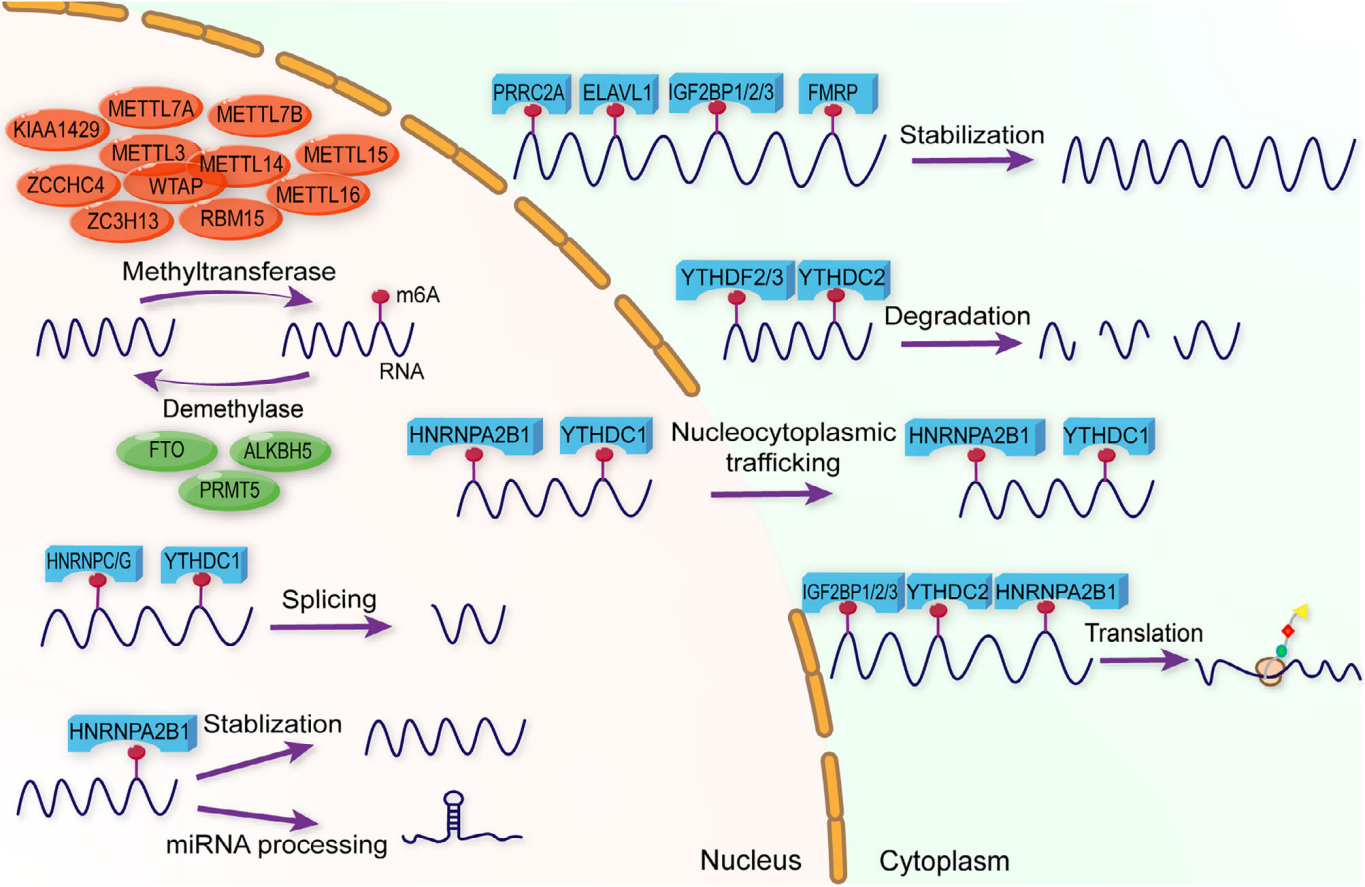
Regulatory proteins of m6A modification. m6A modification is methylated by methyltransferases including METTL3–METTL14–WTPA complex, ZC3H13, RBM15, METTL7A/B, METTL15, METTL16, KIAA1429, and ZCCHC4, demethylated by demethylase including FTO, ALKBH5, and PRM15. Furthermore, m6A modification is recognized by m6A binding proteins that execute diverse biological functions. In nucleus, HNRNPA2B1 and YTHDC1 facilitate mRNA nucleocytoplasmic trafficking while HNRNPC/G and YTHDC1 are involved in RNA splicing. In addition, HNRNPA2B1 stabilizes RNA and promotes miRNA processing. In cytoplasm, PRRC2A, ELAV1, IGF2BP1/2/3, and FMRP promote mRNA stabilization, whereas YTHDF2/3 and YTHDC2 promote mRNA decay. HNRNPA2B1, YTHDC2, and IGF2BP1/2/3 play roles in mRNA translation.

m6A modification is regulated by diverse proteins including writers, erasers, and readers. Writers, specifically methyltransferases, methylate mRNA bases. Erasers, known as demethylases, remove these modifications. Readers are proteins that modulate mRNA metabolism by selectively binding to m6A modifications. Each regulator plays a crucial role in m6A processes, influencing both physiological functions and pathological conditions (Figure 1 and Table 1).

**TABLE 1:** Regulatory proteins of m6A modification.

Type	Regulator	Function	References
Writer	METTL3	Catalyze m6A RNA modification	23, 24
	METTL14	Catalyze m6A RNA modification	23, 24, 25
	WTAP	Assist the localization of METTL3/METTL14 complex into nuclear speckles	26
	METTL5	Promote 18S rRNA m6A modification	27
	METTL16	Promote U6 small nuclear RNA m6A modification and S‐adenosylmethionine (SAM) synthetase pre‐mRNA	32, 33
	KIAA1429	Methylate several target RNA in m6A manner	35
	ZCCHC4	Promote 28S rRNA m6A modification	27, 37
	METTL7A	The sequence between 76 and 172 aa of METTL7A contributes to the methylation of adenosine at 481 of LOC606724.	28
	METTL7B	Induce m6A modification of GPX4, HMOX1, and SOD1 mRNA.	30
	ZC3H13	Anchor WTAP in nuclear and enhance its activity	38
	RBM15	Catalyze m6A RNA modification	36
Eraser	FTO	Remove m6A modification	44
	ALKBH5	Remove m6A modification	47
	PRMT5	Inhibit RNA m6A methylation by enhancing the nuclear translocation of ALKBH5	50
Reader	YTHDF1	Promote m6A‐modified mRNA translation	51, 52, 54
	YTHDF2	Recognize m6A modification and degrade mRNA	51
	YTHDF3	Enhance the translation of m6A‐enriched transcripts and mRNA degradation	53
	YTHDC1	Promote targeted mRNA splicing and nuclear export of mRNA	55, 70
	YTHDC2	Enhance targeted mRNA translation efficiency and decrease mRNA abundance	56
	IGF2BP1	Promote m6A‐modified mRNA stability and translation in cytoplasm	59
	IGF2BP2	Promote m6A‐modified RNA translation in cytoplasm	57
	IGF2BP3	Promote m6A‐modified RNA translation in cytoplasm	58
	HNRNPC	Promote m6A‐modified RNA splicing in nuclear	74
	HNRNPA2B1	Interact with and promote primary miRNA processing in nuclear Promote target mRNA nucleocytoplasmic trafficking Promote mRNA stabilization	60, 61, 72, 73
	HNRNPG	Promote m6A‐modified RNA splicing	75
	PRRC2A	Stabilize m6A‐modified mRNA	62
	ELAVL1	Interact with other m6A regulators and stabilize target RNA	40, 63, 76
	FMRP	Stabilize m6A‐modified mRNA	64

Abbreviations: METTL3, methyltransferase‐like 3; METTL14, methyltransferase‐like 14; WTAP, Wilms tumor 1‐associating protein; METTL5, methyltransferase‐like 5; METTL16, methyltransferase‐like 16; ZCCHC4, zinc finger CCHC domain‐containing protein 4; METTL7A, methyltransferase‐like 7A; METTL7B, methyltransferase‐like 7B; ZC3H13, zinc finger CCCH domain containing protein 13; RBM15, RNA binding motif protein 15; FTO, Fat mass and obesity‐associated protein; ALKBH5, human AlkB homolog 5; PRMT5, Protein arginine methyltransferase 5; YTHDF1, YT521‐B homology (YTH) domain‐containing family protein 1; YTHDF2, YT521‐B homology (YTH) domain‐containing family protein 2; YTHDF3, YT521‐B homology (YTH) domain‐containing family protein 3; YTHDC1, YT512‐B homology domain‐containing protein 1; YTHDC2, YT512‐B homology domain‐containing protein 2; IGF2BP1, insulin‐like growth factor 2 mRNA‐binding protein 1; IGF2BP2, insulin‐like growth factor 2 mRNA‐binding protein 2; IFG2BP3, Insulin‐like growth factor 2 mRNA‐binding protein 3; HNRNPC, heterogeneous nuclear ribonucleoprotein C; HNRNPA2B1, heterogeneous nuclear ribonucleoprotein A2B1; HNRNPG, heterogeneous nuclear ribonucleoprotein G; PRRC2A, proline‐rich coiled‐coil 2A; ELAVL1, ELAV‐like RNA‐binding protein 1; FMRP, fragile X mental retardation protein

### 2.1 m6A writers

m6A writers primarily comprise the m6A methyltransferase complex, which includes methyltransferase‐like 3 (METTL3), methyltransferase‐like 14 (METTL14), and Wilms tumor 1‐associating protein (WTAP).^23^ METTL3, the first discovered writer and a key member of this complex, is a 70‐kDa protein that contains a methyltransferase domain that methylates RNA. Similarly, METTL14 can methylate RNA. In addition, when METTL3 and METTL14 form a complex with a 1:1 stoichiometry,^24^ METTL3 and METTL14 have much better m6A methyltransferase activity than either alone.^25^ Although WTAP does not directly methylate RNA, it significantly influences the structure and substrate specificity of the METTL3/METTL14 complex. In nuclear speckles, WTAP is associated with the accumulation of METTL3 and METTL14.^26^ Moreover, other important methyltransferases have been reported, including methyltransferase‐like 5 (METTL5),^27^ methyltransferase‐like 7A (METTL7A),^28^, ^29^ methyltransferase‐like 7B (METTL7B),^30^, ^31^ methyltransferase‐like 16 (METTL16),^32^, ^33^ KIAA1429,^34^, ^35^ RNA binding motif protein 15 (RBM15),^36^ zinc finger CCHC domain‐containing protein 4 (ZCCHC4),^37^ and zinc finger CCCH domain containing protein 13 (ZC3H13).^38^ Emerging evidence has revealed the important role of writers in tumorigenesis,^39^ tumor metastasis,^40^ and immunotherapy.^41^ m6A writers can upregulate the m6A methylation of cancer‐related genes. For example, Wei et al.^42^ demonstrated that METTL3 accelerates gastric cancer (GC) progression through the ADAMTS9‐mediated phosphatidylinositol‐3 kinase (PI3K)/V‐akt murine thymoma viral oncogene homolog (AKT) pathway. Zhou et al.^43^ found that the METTL3/YTHDF2 m6A axis accelerated colorectal carcinogenesis. Collectively, m6A writers promote RNA methylation and induce multiple biological functions.

### 2.2 m6A erasers

m6A erasers, also known as m6A demethylases, remove m6A from RNA and decrease m6A levels. Fat mass and obesity‐associated protein (FTO) has been considered as the first m6A demethylase since He et al.^44^ discovered that m6A was the main substrate of FTO in nuclear RNA in 2011. FTO not only plays an essential role in obesity by regulating adipogenic pathways and inducing preadipocyte differentiation to facilitate adipogenesis but also participates in tumor processes.^45^, ^46^ Another key demethylase, human AlkB homolog 5 (ALKBH5), also plays a role in m6A modifications associated with various diseases, especially tumors. ALKBH5 belongs to the alkB family of dioxygenases, which regulate oxidative demethylation to modulate the repair of N‐alkylated nucleobases.^47^, ^48^ ALKBH5 participates in multiple cancer or noncancer processes.^49^ Protein arginine methyltransferase 5 (PRMT5) is a new demethylase that inhibits RNA m6A modification by enhancing the nuclear translocation of ALKBH5.^50^ In summary, m6A erasers play important roles in demethylating RNA and inducing subsequent functions.

### 2.3 m6A readers

m6A readers, also called m6A‐binding proteins, can recognize and bind to m6A‐modified transcripts to regulate the expression and function of several genes during various processes. Several m6A readers have been identified, including the YTHDF family,^51^, ^52^, ^53^, ^54^ YTHDC family,^55^, ^56^ IGF2BP family,^57^, ^58^, ^59^ RNA‐binding protein heterogeneous nuclear ribonucleoprotein (HNRNP) family,^60^, ^61^ proline‐rich coiled‐coil 2A (PRRC2A),^62^ ELAV‐like RNA‐binding protein 1 (ELAVL1),^63^ and fragile X mental retardation protein (FMRP).^64^ The YTHDF family includes three proteins: YTHDF1, YTHDF2, and YTHDF3. YTHDFs have different functions. YTHDF1 recognizes m6A‐modified RNA and promotes its translation into the cytoplasm. YTHDF2 participates in the degradation of m6A‐modified RNA. YTHDF3 enhances mRNA translation and degradation. YTHDFs regulate cancer progression in an m6A‐dependent manner. For instance, Chen et al.^65^ found that YTHDF1 could facilitate the translation of FOXM1 via m6A modification, which subsequently promotes breast cancer (BC) progression. However, a new model for YTHDFs provided by Zaccara and Jaffrey^66^ illustrates that YTHDFs function together to mediate the degradation of m6A‐modified RNA. Liquid‒liquid phase separation (LLPS) is the formation of several membraneless condensates. Interestingly, YTHDFs have the potential to form condensates. Specifically, the N‐terminal of YTHDFs is mainly an intrinsically disordered region (IDR), whereas the C‐terminal consists of the m6A‐binding YTH domain. Both play vital roles in the formation of the condensates.^67^ Zou et al.^68^ found that YTHDF1 and YTHDF2 can form different granules because of their diverse low‐complexity regions. In addition, Fu et al.^67^ found that under oxidative stress, YTHDF1 and YTHDF3 are abundant in stress granules rather than in processing bodies (P‐bodies). However, YTHDF2 was abundant in both stress granules and P‐bodies.^67^ Stress granules and P‐bodies have several functions, including metabolic reprogramming, targeting and silencing of specific mRNAs via the RNA‐induced silencing complex, and restoring and permitting the translation of key specific mRNAs.^69^ YTHDCs are YTH domain‐containing proteins, including YTHDC1 and YTHDC2. YTHDC1 mediates nuclear export of m6A‐modified mRNA and facilitates mRNA splicing.^55^, ^70^ Recent studies have also demonstrated its ability to form membraneless organisms such as nuclear condensates via LLPS, which may be associated with gene expression, transcript splicing, and nucleocytoplasmic export.^71^ YTHDC2 enhances translation efficacy and destabilizes cytoplasm.^56^ IGF2BP family refers to Insulin‐like growth factor 2 mRNA‐binding proteins, which mainly consist of IGF2BP1, IGF2BP2, and IGF2BP3. IGF2BPs are key readers of m6A modifications that stabilize mRNA and promote its translation in the cytoplasm.^59^ HNRNPA2B1 has multiple functions in m6A modification, including processing of miRNA,^72^ promotion of nucleocytoplasmic trafficking,^73^ and stabilization of m6A‐modified mRNA.^60^, ^61^ HNRNPC is an abundant nuclear RNA‐binding protein that recognizes and splices m6A‐modified RNA.^74^ HNRNPG recognizes RNA via RNA recognition motif (RRM) and Arg‐Gly‐Gly (RGG) motifs and splices m6A‐modified RNA.^75^ PRRC2A plays a role in target RNA stabilization. For example, PRRC2A stabilizes Olig2 mRNA via the GGACU motif.^62^ ELAVL1 (also known as HuR) interacts with other m6A regulators and promotes m6A‐modified RNA stabilization.^40^, ^76^ FMRP sustains m6A‐modified stability and maintains its expression via m6A modification.^77^ Collectively, m6A‐binding proteins recognize m6A‐modified mRNA and influence their stability, localization, and translation. Notably, condensate formation of reader proteins induced by LLPS is an important direction for future research.

## 3 PTMs OF M6A REGULATORS

**FIGURE 2:**
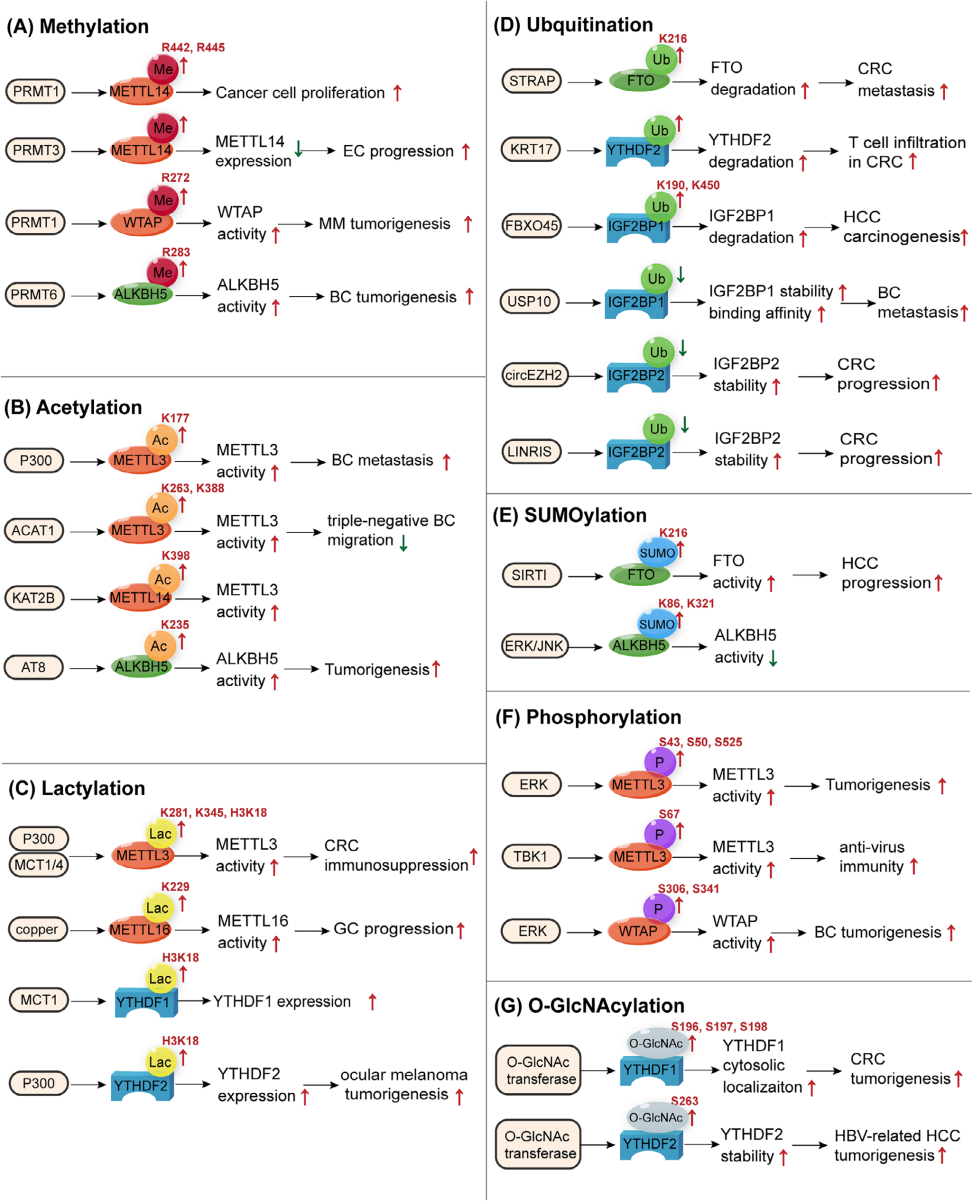
Posttranslational modifications (PTMs) of m6A regulators. m6A regulators can be modified in diverse post‐translational ways. (A) Role of methylation in m6A regulators. (B) Role of acetylation in m6A regulators. (C) Role of lactylation in m6A regulators. (D) Role of ubiquitination in m6A regulators. (E) Role of SUMOylation in m6A regulators. (F) Role of phosphorylation in m6A regulators. (G) Role of O‐GlcNAcylation in m6A regulators. EC, endometrial carcinoma; MM, multiple myeloma; BC, breast cancer; CRC, colorectal cancer; GC, gastric cancer; HCC, hepatocellular carcinoma.

As a posttranscriptional RNA modification, m6A is regulated by writers, erasers, and readers. Moreover, these regulatory proteins undergo PTMs. We investigated various forms of modifications, such as methylation, acetylation, lactylation, ubiquitination, SUMOylation, phosphorylation, and O‐GlcNAcylation (Figure 2 and Table 2).

**TABLE 2:** Posttranslational modifications of m6A regulators.

Modification	Regulator	Site	Factors	Function	References
Methylation	METTL14	R442 R445	PRMT1	Methylate METTL14 and promote cancer cell proliferation	78
	METTL14		PRMT3	Methylate METTL14 and upregulate its expression to promote EC progression	79
	WTAP	R272	PRMT1	Methylate WTAP and promote the oxidative phosphorylation of MM	80
	ALKBH5	R283	PRMT6	Methylate ALKBH5, which promotes aerobic glycolysis and tumorigenesis in BCs	81
Acetylation	METTL3	K177	P300	Catalyze METTL3 acetylation and affect subcellular localization of METTL3 to impede cancer metastasis in BC	83
	METTL3	K263 K388	ACAT1	Catalyze METTL3 acetylation, inhibit migration of cancer cells via NR2F6/ACAT/METTL3 axis	84
	METTL3‐METTL14‐WTAP complex		Sulfatide	Acetylation of the writer complex down‐regulates MTF1 expression and the growth of HCC cells	85
	METTL14	K398	KAT2B	Promote METTL14 acetylation and protein stability	86
	ALKBH5	K235	AT8	Regulate demethylase activity and promote tumorigenesis	87
	IGF2BP2	K530	SIRT1	Loss of SIRTI acetylates IGF2BP2 to recruit the nuclease XRN2 and degrade the ATP6V1A transcript.	88
Lactylation	METTL3	H3K18 K281 K345	P300	H3K18la facilitate the transcription of METTL3 to promote its expression; lactylation of METTL3 at K281 and K345 enhance its capture of m6A‐modified RNA to promote immunosuppression in CRC	95
	METTL16	K229	Copper	Lactylation of METTL16 at K229 promotes its methyltransferase activity and promotes FDX1 expression to induce cuproptosis	96
	YTHDF2	H3K18	P300	H3K18la facilitates the transcription of YTHDF2 and then promote the degradation of m6A‐modified PER1 and TP53 mRNAs to promote tumorigenesis in ocular melanoma	93
	YTHDF1	H3K18	MCT1	H3K18la facilitates the transcription of YTHDF1 and its protein expression by binding to the promoter	94
Ubiquitination	YTHDF2		KRT17	Promote YTHDF2 degradation and therefore stabilize CXCL10 mRNA to induce T cell infiltration in CRC	102
	IGF2BPs		TRIM25, circNDUFB2	Degrade IGF2BPs proteins and activate antitumor immunity during NSCLC progression	103
	IGF2BP2		circEZH2	Block IGF2BP2 degradation and promote its function of stabilizing CREB1 mRNA to promote CRC progression	105
	IGF2BP2	K139	LINRIS	Block IGF2BP2 ubiquitination and facilitate its glycolysis in CRC	106
	IGF2BP1		USP10	Block IGF2BP1 ubiquitination and stabilize IGF2BP1, enhance its binding to m6A‐modified CPT1A mRNA, leading to breast cancer metastasis	107
	IGF2BP1	K190 K450	FBXO45	Increase IF2BP1 ubiquitination and subsequent PLK1 upregulation to promote HCC carcinogenesis	104
	FTO	K216	STRAP	Increase FTO ubiquitination and promote its degradation to stabilize MTA1 mRNA and promote CRC metastasis	101
	METTL14		HRD1	Promote METTL14 ubiquitination and degradation to suppress endoplasmic reticulum‐related liver disease	100
SUMOylation	YTHDF2	K281, K571, K572	PIAS1	Promote viral RNA decay to restrict EBV replication	110
	YTHDF2	K571	SUMO1	Increase its binding affinity of m6A‐modified mRNAs and decrease‐related gene expression	111
	METTL3	K177, K211, K212, K215	SUMO1	Represses METTL3 m6A methytransferase activity	113
	ALKBH5	K86, K321	ERK/JNK	Inhibit ALKBH5 demethylation activity and increase m6A modification to protect genomic integrity of cells in response to ROS	109
	FTO	K216	SIRT1	Promote FTO SUMOylation and its degradation to decrease GNAO1 expression and promote HCC	112
Phosphorylation	METTL3	S43, S50, S525	ERK	Stabilize m6A methyltransferase complex to upregulate m6A levels and promote tumorigenesis	117, 118
	METTL3	S67	TBK1	Phosphorylate METTL3 and promote its activation, enhancing antivirus immunity	120
	WTAP	S306, S341	ERK	Stabilize m6A methyltransferase complex to upregulate m6A levels and promote tumorigenesis	117
	WTAP	S341	ERK	Promote WTAP phosphorylation and stabilize WTAP protein, further promoting RNA m6A methylation of ENO1, impacting the glycolytic activity of BC cells.	119
	YTHDF2	S39, T381	EGFR, SRC, ERK	Promote YTHDF2 phosphorylation and stabilization, subsequently facilitate m6A‐modified LXRA and HIVEP2 mRNA decay to promote glioblastoma tumorigenesis	121
O‐GlcNAcylation	YTHDF2	S263	OGT	Enhance YTHDF2 stability and oncogenic activity in HBV‐related HCC	123
	YTHDF1	S196, S197, S198	OGT	Promote YTHDF1 cytosolic localization and upregulate downstream target gene expression to promote CRC tumorigenesis	124

### 3.1 Methylation

Arginine methylation of m6A regulatory proteins, induced by protein arginine methyltransferases (PRMTs), plays a vital role in cancer initiation and progression. Methylated METTL14 participates in cancer development. For instance, PRMT1‐mediated methylation of METTL14 at the C‐terminus is important for its function in catalyzing m6A modification, which promotes cancer cell proliferation.^78^ PRMT3 methylates METTL14, which downregulates its expression and promotes EC progression by influencing glutathione peroxidase 4 (GPX4) expression.^79^ WTAP is methylated by PRMT1, which targets the oxidative phosphorylation of multiple myeloma (MM) cells and promotes carcinogenesis via m6A modification of NDUFS6.^80^ PRMT6 can methylate the m6A eraser ALKBH5 at R283, which upregulates LDHA expression and promotes aerobic glycolysis and tumorigenesis in BC.^81^ In conclusion, arginine methylation of the m6A regulator is a potent target for cancer modulation.

### 3.2 Acetylation

Acetylation is a PTM.^82^ METTL3 is acetylated by P300 at K177. Acetylated METTL3 dampens its subcellular localization and function in BC cells, impeding cancer metastasis.^83^ Similarly, METTL3 is acetylated by acetyl‐CoA acetyltransferase 1 (ACAT1) at residues K263 and K388, which stabilizes METTL3 and suppresses triple‐negative BC migration and invasion via the NR2F6/ACAT/METTL3 axis.^84^ Acetylation of the m6A writer complex METTL3–METTL14–WTAP can be induced by sulfatide, thus regulating MTF1 expression to promote the growth of hepatocellular carcinoma (HCC) cells.^85^ KAT2B, a lysine acetyltransferase, catalyzes METTL14 acetylation at K398 and increases METTL14 protein stability to upregulate m6A methylation of Spi2a mRNA, which inactivates the NF‐κB pathway.^86^ ALKBH5 is acetylated by lysine acetyltransferase 8 (AT8) at K235, which enhances its demethylase activity.^87^ IGF2BP2 is acetylated at K530. Loss of its deacetylase SIRT1 recruits nuclease XRN2 to degrade the ATP6V1A transcript.^88^ All the abovementioned studies show that acetylation can regulate the function of m6A regulatory proteins to influence disease processes.

### 3.3 Lactylation

The Warburg effect involves an increase in glycolytic metabolism even in the presence of O_2_, which is crucial for carcinogenesis, metastasis and drug resistance.^89^, ^90^ Lactate, a product of glycolytic metabolism, has recently gained increasing attention owing to its potential biological functions in cancer. Lactylation is a novel epigenetic modification.^91^ In 2019, Zhang et al.^92^ reported the metabolic regulation of histone lactylation gene expression. Current evidence indicates that lactylation is involved in m6A modifications. Histone lactylation is abundant in the promoters of m6A regulators and significantly influences their expression to a great extent. For instance, YTHDF1 and YTHDF2 expression are facilitated by H3K18la.^93^, ^94^ In contrast, lactylation directly modifies m6A regulators. Xiong et al.^95^ identified two lactylation modification sites, K281 and K345, in the zinc‐finger domain of METTL3. The lactylation of METTL3 enhances its binding to Jak1 mRNA and therefore promotes the immunosuppression of colorectal cancer (CRC). Sun et al.^96^ found that high copper stress could induce METTL16 lactylation at K229, which upregulates the activity of METTL16 and, therefore, promotes FDX1 expression to facilitate carcinogenesis in GC. In summary, lactylation is important and may provide a new strategy for m6A modification in cancer cells.

### 3.4 Ubiquitination

Ubiquitin is a small protein consisting of 76 amino acids that assists in protein degradation by the S26 proteasome.^97^ Protein ubiquitination is a ubiquitin‐dependent PTM that affects all cellular processes.^98^ Ubiquitination plays a key role in translation by modifying ribosomal and regulatory proteins.^99^ In this study, we focused on its role as an m6A regulator. METTL14 is ubiquitinated and degraded by HRD1 to suppress endoplasmic reticulum‐related liver diseases.^100^ Serine/threonine kinase receptor‐associated protein (STRAP) ubiquitinates FTO at K216, promotes its degradation, stabilizes MTA1 mRNA, and promotes CRC metastasis.^101^ KRT17 promotes YTHDF2 degradation via ubiquitination, which stabilizes CXCL10 mRNA and induces T‐cell infiltration in CRC.^102^ IGF2BP protein ubiquitination can be upregulated by circNDUFB2 and TRIM25 (a kind of E3 ubiquitin ligase), leading to antitumor immunity in non‐small cell lung cancer (NSCLC).^103^ IGF2BP1 is ubiquitinated by the E3 ubiquitin ligase FBXO45 at K190 and K450, which subsequently induces PLK1 upregulation and HCC carcinogenesis.^104^ Additionally, blocking the ubiquitination of IGF2BP proteins can suppress their degradation and promote their binding to RNA.^105^, ^106^, ^107^ These studies demonstrate that ubiquitination mainly affects m6A regulator proteins by promoting degradation.

### 3.5 SUMOylation

SUMOylation is a novel PTM associated with small ubiquitin‐like modifiers that participate in cancer development of cancer.^108^ SUMOylation of m6A regulators influences their functions. For example, extracellular signal‐regulated kinase (ERK)/c‐Jun N‐terminal kinases (JNK) signaling‐induced SUMOylation of ALKBH5 represses its demethylase activity.^109^ YTHDF2 can be SUMOylated by PIAS1 at K281, K571, and K572, thereby facilitating the degradation of viral RNA and suppresses the replication of EBV.^110^ Additionally, YTHDF2 SUMOylation at K571 promotes mRNA decay and tumorigenesis.^111^ FTO is modified by SIRT1 at K216, which promotes FTO SUMOylation and degradation, decreases GNAO1 expression, and promotes HCC.^112^ METTL3 is reported to be SUMOylated at K177, K211, K212, and K215, which represses METTL3 m6A methyltransferase activity.^113^

### 3.6 Phosphorylation

Phosphorylation is an extensively studied PTM of proteins.^114^ Over the past 25 years, protein phosphorylation has been observed at the serine, threonine, and tyrosine residues.^115^ Protein phosphorylation regulates important cellular functions.^116^ ERK phosphorylates and stabilizes the METTL3/14‐WTAP methyltransferase complex, which increases m6A methylation and promotes tumorigenesis.^117^ Specifically, METTL3 is typically modified at S43, S50, and S525,^117^, ^118^ whereas WTAP is modified at S306 and S341.^117^, ^119^ METTL3 is also phosphorylated at residue S67, which promotes its activation and helps m6A modification to stabilize IRF3 mRNA, leading to antiviral immunity.^120^ YTHDF2 can be phosphorylated and stabilized by EGFR/SRC/ERK signaling at S39 and T381, which facilitates the degradation of LXRA and HIVEP2 mRNA, leading to glioblastoma (GBM) tumorigenesis.^121^ Therefore, phosphorylation mainly stabilizes m6A regulators and activates their function in target mRNAs to modulate downstream gene expression and cellular processes. Current studies on SUMOylation of m6A regulators are limited and require further research.

### 3.7 O‐GlcNAcylation

O‐linked N‐acetylglucosaminylation (O‐GlcNAcylation) is a type of glycosylation related to O‐GlcNAc at serine or threonine residues.^122^ Yang et al.^123^ found that YTHDF2 is modified by O‐GlcNAc transferase 8(OGT8) at S263, which stabilizes YTHDF2 and enhances its activity by inhibiting its ubiquitination. Subsequently, O‐GlcNAc‐modified YTHDF2 stabilizes the minichromosome maintenance protein 2/5 transcripts to facilitate the onset of HBV‐related HCC.^123^ YTHDF1 O‐GlcNAcylation at S196, S197, and S198 promotes YTHDF1 cytosolic localization and upregulates the expression of downstream target genes to promote CRC tumorigenesis.^124^ The O‐GlcNAcylation of m6A regulators has a significant impact on diseases, and related research has great potential.

These studies show that diverse PTMs in m6A regulators significantly influence their functions in cancer.

## 4 M6A MODIFICATIONS WITH CHROMATIN REGULATION IN CANCER

**FIGURE 3:**
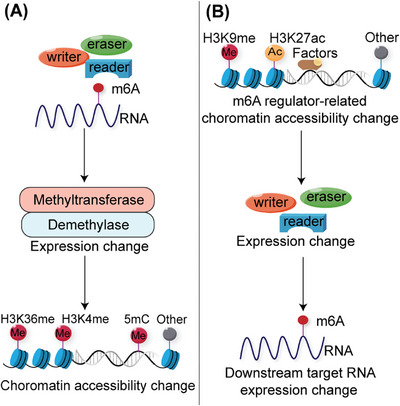
m6A modifications with chromatin regulation in cancer. (A) m6A modification alters histone/DNA methyltransferase or demethylase expression, subsequently affecting chromatin accessibility via histone/DNA modification. (B) Several important factors correlate with m6A regulator‐related chromatin accessibility, which changes the expression of m6A regulator proteins, accordingly affecting downstream target gene expression.

Chromatin and transcriptional states, which are dynamically regulated by epigenetic modification networks, are critical for establishing and maintaining cellular identity. Studies have shown that the m6A modification can regulate the chromatin state, known as chromatin accessibility, to affect transcriptional activity (Figure 3).

Changes in chromatin accessibility due to m6A‐modified target genes significantly impact downstream transcription levels. Liu et al.^6^ discovered that METTL3 mediates m6A modification of chromosome‐associated regulatory RNAs, including promoter‐associated RNAs, enhancer RNAs, and repeat RNAs. YTHDC1 facilitates the nuclear degradation of a subset of these m6A‐modified RNAs, ultimately leading to reduced chromatin accessibility and downstream transcription inhibition.^6^ The interactions between RNA m6A and DNA/histone modifications are important for physiological and pathogenic processes. In esophageal squamous cell carcinoma (ESCC) cells, METTL3 mediates m6A modification of the DNA demethylase TET1. The m6A reader FXR1 recognizes m6A RNA and recruits TET1 to genomic sites to demethylate DNA, leading to chromatin accessibility and reprogramming of gene transcription.^125^ In PDAC cells, m6A super‐enhancer RNAs modified by the METTL3‐CFL1 complex are recognized by YTHDC2, which recruits H3K4 methyltransferase MLL1 to catalyze H3K4me3 deposition, thus enhancing local chromatin accessibility and oncogene transcription.^126^ ALKBH5‐demethylated lncRNA SNHG15 promotes myeloma tumorigenicity by increasing chromatin accessibility and recruiting the H3K36me3 modifier SETD2.^127^

Changes in the chromatin state of m6A regulators alter the levels of m6A modifications in downstream target genes, influencing their transcriptional activity. PARP1 regulates the transcription factor NFIC, and the activation of METTL3 transcription relies on PARP1 in conjunction with the METTL3 promoter. Upon irradiation or treatment with a PARP1 inhibitor, PARP1 dissociates from the METTL3 promoter chromatin. This leads to reduced accessibility of nuclear factor I‐C (NFIC) and TATA‐box binding protein (TBP), resulting in the repression of METTL3 expression and RNA m6A methylation.^128^ KDM4C regulates ALKBH5 expression to maintain leukemia stem cell (LSC) function in acute myeloid leukemia (AML) by increasing chromatin accessibility at ALKBH5 locus, reducing H3K9me3 levels, and promoting v‐myb avian myeloblastosis viral oncogene homolog (MYB) and RNA Polymerase II (Pol II) recruitment. ALKBH5 affects the mRNA stability of the receptor tyrosine kinase AXL, which is recognized by YTHDF2 in an m6A‐dependent manner.^129^ Temozolomide (TMZ) induces a SOX4‐mediated increase in chromatin accessibility at METTL3 by promoting H3K27ac levels and recruiting RNA polymerase II to upregulate METTL3 expression, which further promotes m6A deposition on the histone methyltransferase EZH2, inhibits nonsense‐mediated mRNA decay, and maintains GBM stem cell properties. This eventually led to increased TMZ resistance in GBM.^130^

The above studies, including the direct or indirect regulation between RNA m6A and DNA/histone modifications, demonstrate extensive interactions between these epigenetic regulatory events and provide new targets for tumor therapy.

## 5 M6A MODIFICATIONS WITH PCD IN CANCER

PCD is a crucial and well‐organized mechanism that maintains cellular homeostasis in response to internal and external stimuli. Within TMEs, the interplay between pro‐death and pro‐survival pathways shapes the complexity and variability of tumor immunity. Previous research underscores PCD's pivotal role in cancer processes and in anticancer immunity.^131^, ^132^, ^133^, ^134^ Various factors regulate PCD, with recent attention focused on m6A modifications. Here, we explore the interrelationship between m6A and diverse forms of PCD—including autophagy, ferroptosis, pyroptosis, cuproptosis, and disulfidaptosis—in both cancer progression and antitumor immunity. Our findings aim to inform novel strategies for future cancer treatments (Table 3).

**TABLE 3:** m6A modifications with programmed cell death in cancer.

PCD	Cancer	Regulator	Function	References
Autophagy	HCC	YTHDF1	HIF‐1α‐induced YTHDF1 upregulates ATG2A and ATG14 translation to promote HCC autophagy and malignancy.	140, 141
	HCC	IGF2BP1	circMDK modified by IGF2BP1 promotes HCC proliferation and metastasis via the miR‐346/874‐3p‐ATG16L1 axis.	142
	HCC	WTAP	WTAP‐mediated m6A modification regulates LKB1 and decreases phosphorylation of AMPK to restrain cell autophagy and promote HCC proliferation.	144
	HCC	METTL3	M6A‐modified FOXO3 by METTL3 activates autophagy‐associated pathways and promotes sorafenib resistance.	152
	EOC	FTO	circRAB11FIP1 promotes FTO‐associated proteins expression and mediates mRNA expression levels of ATG5 and ATG7 in an m6A dependent manner to facilitate autophagy flux.	143
	ccRCC	FTO	FTO/autophagy/SIK2 axis promotes the progression of ccRCC.	145
	GC	FTO, YTHDF2	FTO and YTHDF2 regulate ULK1 expression to modulate autophagy activation and drug resistance.	149
	LSCC	IGF2BP3	IGF2BP3 regulates TMA7‐mediated autophagy and Cisplatin resistance in LSCC.	151
	SCLC	METTL3	METTL3 targets DCP2 to induce Pink1–Parkin pathway‐mediated mitophagy and mitochondrial damage to promote chemoresistance in SCLC.	150
Ferroptosis	GC	YTHDF2	Hypoxia‐induced lncRNA CBSLR interacts with YTHDF2 to decrease CBS mRNA and therefore reduce methylation of the ACSL4 leading to ferroptosis resistance in GC.	187
	PTC	FTO	FTO downregulates SLC7A11 in an m6A dependent manner through ferroptosis to Inhibit PTC proliferation, migration, and invasion.	158
	BC	METTL14	M6A‐modified FGFR4 reduces ferroptosis in recalcitrant HER2‐positive BC via the β‐catenin/TCF4‐SLC7A11/FPN1 axis.	165
	HB	METTL3	METTL3‐mediated SLC7A11 m6A modification enhances HB ferroptosis resistance.	156
Pyroptosis	LC	METTL3, YTHDF2	M6A modification of lncRNA LINC00969 at posttranscriptional levels inhibits pyroptosis and promotes acquired gefitinib resistance in lung cancer.	172
Cuproptosis	GC	METTL16	Lactylation of METTL16 promotes cuproptosis in GC by upregulating m6A modification of FDX1 mRNA and expression of FDX1 protein.	96

Abbreviations: HCC, hepatocellular carcinoma; EOC, epithelial ovarian cancer; ccRCC, clear cell renal cell carcinoma; GC, gastric cancer; LSCC, laryngeal squamous cell carcinoma; SCLC, small cell lung cancer; PTC, papillary thyroid cancer; BC, breast cancer; HB, hepatoblastoma; LC, lung cancer.

### 5.1 Autophagy

Autophagy is highly conserved in eukaryotes. It maintains cellular homeostasis and metabolism.^135^ When stressed by internal or external stimuli, cells combine autophagosomes with lysosomes to form autolysosomes, membrane structures that engulf and degrade aged or injured organelles, misfolded proteins, and pathogens to regulate cell homeostasis of cells.^136^ Additionally, this process plays a vital role in tumors by promoting and inhibiting tumorigenesis and progression.^137^

m6A modifications are closely associated with autophagy. The upregulation of m6A RNA modification is helpful for autophagosome formation when nutrients are deficient.^138^ The m6A modification can regulate autophagy‐related genes (ATGs), ultimately influencing their function and promoting the onset of various diseases, including tumors.^139^ For instance, Hao et al.^138^ reported that YTHDF3 responds to METTL3‐associated m6A hypermethylation and recruits eIFs to promote FOXO3 translation, consequently activating a subset of ATGs and leading to autophagy. In liver tissues, m6A RNA modification plays a complex role in tumor progression. If not addressed, liver fibrosis can progress to cirrhosis and cancer. Hepatic stellate cells (HSCs) play an important role in myofibroblast matrix production during this physiological and pathological process. Shen et al.^140^ showed that YTHDF1 stabilizes BECN1 mRNA and promotes autophagy activation via m6A modification in HSCs. The hypoxia‐inducible factor‐1α (HIF‐1α) drove YTHDF1 to bind to the m6A‐modified mRNA of ATG2A and ATG14, which can contribute to the translation of ATG2A and ATG14, thereby promoting the survival of HCC under hypoxic conditions and its progression.^141^ One study identified an oncogenic circRNA, circMDK, as a potential biomarker for HCC, because its upregulation with m6A modification upregulates ATG16L1, resulting in the activation of the PI3K/AKT/mTOR signaling pathway to promote cell proliferation, migration, and invasion.^142^ Interestingly, circRNAs have gained considerable attention in recent years. CircRAB11FIP1 mediates the expression of ATG5 and ATG7 via m6A, thus promoting epithelial ovarian cancer.^143^ LKB1 is regulated by WTAP via m6A modification and then phosphorylated AMPK. At the same time, researchers found that knockdown of WTAP could upregulate the level of autophagy and inhibit hepatoblastoma (HB) cell proliferation.^144^ Similarly, the progression of clear cell renal cell carcinoma (ccRCC) can be regulated by FTO because it inhibits autophagy and promotes tumorigenesis through an m6A‐IGF2BP2‐dependent mechanism, indicating that FTO can be a prognostic biomarker and a promising target in ccRCC.^145^

In parallel, m6A‐dependent autophagy plays an important role in antitumor drug resistance. From a pharmacokinetic perspective, m6A modifications influence drug transport and metabolism. This may be related to several membrane transporter proteins such as ATP‐binding cassette proteins. In addition, m6A can alter drug targets to regulate drug response and resistance.^146^ Autophagy promotes anticancer drug resistance to protect tumor cells from survival.^147^ Furthermore, m6A modification can modulate ATGs (ATG5 and ATG7) to regulate the formation and progression of autophagosomes, thus influencing autophagy and promoting the survival and anticancer resistance of tumor cells.^148^ However, the underlying mechanisms remain unclear. FTO‐mediated cisplatin resistance in GC is attributed to the inhibition of Unc‐51‐like kinase (ULK1)‐mediated autophagy.^149^ Sun et al.^150^ found that METTL3 is a marker for poor prognosis of small‐cell lung cancer (SCLC) and is highly expressed in chemoresistant SCLC cells through Pink1–Parkin pathway‐mediated mitophagy. Translation machinery‐associated 7 homolog (TMA7) plays a key role in the carcinogenesis of laryngeal squamous cell carcinoma (LSCC) and cisplatin resistance via the IGF2BP3/TMA7/UBA2 axis.^151^ In liver cancer, sorafenib resistance is induced by m6A‐dependent, FOXO3‐mediated autophagy.^152^ m6A plays a crucial role in the onset, progression, and drug resistance of multiple tumors by regulating autophagy, suggesting a promising breakthrough in future antitumor treatments.

### 5.2 Ferroptosis

Ferroptosis, first described by Dixon et al. in 2012,^153^ is a new nonapoptotic form of PCD. Biochemically, ferroptosis is a ROS‐dependent PCD characterized by iron accumulation and lipid peroxidation.^154^ Emerging evidence has demonstrated its mechanisms, including the suppression of system Xc−, GPX4, mitochondrial voltage‐dependent anion channels, and P53.^155^ System Xc is an amino acid antitransporter composed of two subunits, SLC7A11 and SLC3A2.^153^ GPX4 plays a pivotal role in the induction and regulation of ferroptosis by inhibiting lipid peroxide formation.

Studies have indicated that m6A modification can regulate cancer cell ferroptosis via m6A‐modifying ferroptosis‐associated mRNA to modulate these mechanisms. For instance, SLC7A11 has been widely demonstrated to be m6A‐modified by several m6A regulators. SLC7A11 mRNA stability can be promoted, and its expression can be upregulated in a METTL3 manner, resulting in tumor growth and ferroptosis resistance in HB^156^ and lung adenocarcinoma.^157^ SLC7A11 can be downregulated by FTO to inhibit thyroid cancer progression via ferroptosis.^158^ ALKBH5 can decrease the expression of SLC7A11 by repressing the m6A modification, which promotes ferroptosis in CRC^159^ and thyroid cancer.^160^ NF‐κB activating protein (NKAP) serves as a novel suppressor of ferroptosis. NKAP binds to m6A and promotes SLC7A11 mRNA splicing to protect GBM cells from ferroptosis.^161^ METTL16 increases GPX4 expression by modifying m6A to inhibit ferroptosis and promote BC.^162^ The mature GPX4 mRNA contains three m6A binding motifs. RUNX1 intronic transcript 1 (RUNX1‐IT1) directly binds to IGF2BP1 and promotes LLPS to increase GPX4 mRNA stability, thereby blocking ferroptosis and promoting BC carcinogenesis.^163^ Erianin, a low‐molecular‐weight bibenzyl natural product extracted from Dendrobium chrysotoxum, induces ferroptosis in renal cancer stem cells by promoting the m6A methylation of ALOX12 and P53 mRNA.^164^ m6A‐modified FGFR4 reduces ferroptosis in recalcitrant HER2‐positive BC via the β‐catenin/TCF4‐SLC7A11/FPN1 axis.^165^

Furthermore, m6A‐associated ferroptosis plays an important role in anticancer immunity and immunotherapy. Immunotherapy‐activated CD8+ T cells can regulate tumor ferroptosis to enhance the antitumor effects.^166^ Several studies have aimed to bioinformatically analyze the relationship between m6A modification, ferroptosis, and immunity in cancers. SLC17A9 is associated with tumor immune infiltration, m6A modification, and ferroptosis in HCC.^167^ Li et al.^168^ found that the expression of BTBD10 (an activator of the Akt family) was correlated with some m6A‐associatedgenes, ferroptosis‐related genes, and immune checkpoints in HCC. Wang et al.^169^ showed that YTHDF1 suppresses CD8+ T cell‐related anticancer effects and ferroptosis by stabilizing programmed cell death ligand 1 (PD‐L1) transcripts, which are important for prostate cancer cells to evade effector T cell cytotoxicity and CD8+ T cell‐mediated ferroptosis. In summary, the m6A modification regulates ferroptosis in cancer cells by modulating the expression of ferroptosis‐associated mRNAs. m6A‐modulated ferroptosis also participates in cancer immunity and immunotherapy. However, the underlying mechanisms remain largely unknown. Therefore, further research is required to develop novel cancer treatment strategies.

### 5.3 Pyroptosis

Pyroptosis is a gasdermin‐mediated PCD process mainly activated by caspases. Pyroptotic cells are swollen and their plasma membrane ruptures. METTL3 suppresses pyroptosis in retinal pigment epithelium cells by targeting the miR‐25‐3p/PTEN/Akt signaling cascade.^170^ YTHDF1 inhibits caspase‐1‐dependent pyroptosis by upregulating the WW domain‐containing E3 ubiquitin protein ligase 1.^171^ LINC00969 promotes acquired gefitinib resistance by decreasing NLRP3 levels via m6A modification to inhibit pyroptosis in lung cancer.^172^ Additionally, bladder cancer, GC, BC, and melanoma are associated with pyroptosis‐related lncRNAs and m6A modification.^173^, ^174^, ^175^, ^176^ However, the underlying mechanisms remain to be elucidated.

### 5.4 Cuproptosis

Cuproptosis is a newly identified form of PCD that is dependent on copper (Cu). This process is characterized by Cu‐targeting and binding to lipoylated components within the tricarboxylic acid cycle, which ultimately induces proteotoxic stress and leads to cell death. Current research indicates that cuproptosis may be correlated with various cancer signaling pathways, including EGFR, PDK1, PI3K, MAPK, MYC, and Notch.^177^ Sun et al.^96^ found that tumor tissues had higher Cu and lactate contents than normal tissues in GC. In addition, they demonstrated that under high Cu stress, lactylation of METTL16 at K229 upregulated the m6A methylation levels of FDX1 mRNA and FDX1 protein expression, which triggered cuproptosis.^96^ Nucleophosmin 1 (NPM1) is a biomarker for gastrointestinal cancer. Researchers found that NPM1 is associated with anticancer immunity, m6A modification, and cuproptosis. Cuproptosis‐related genes can be used to predict the prognosis of various cancers, including lung adenocarcinoma,^178^, ^179^ BC,^180^ and HCC^181^, ^182^ in an m6A‐associated manner. However, little is known about the specific role of m6A and further studies are needed to uncover this mystery.

### 5.5 Disulfidptosis

In contrast to other types of PCDs, cells with high SLC7A11 expression can accumulate cellular disulfides such as cystine. This accumulation induces disulfide stress, which leads to an increasing number of disulfide bonds within the actin cytoskeleton, damaging the cytoskeletal structure, and consequently resulting in cell death.^183^, ^184^, ^185^ Disulfidopathy is a novel approach for metabolic cancer therapy. Recent studies have suggested that metabolic therapy using glucose transporter inhibitors can facilitate disulfidptosis and dampen cancer development.^186^ Disulfidopathy is a potential target for cancer treatment. However, current studies require bioinformatics analyses. Further research is required to explore this mechanism, and m6A modifications may provide an important perspective.

m6A modification plays a pivotal role in regulating the survival and death of cancer cells. PCD serves as a critical modulator of cancer immunity, influencing the function of immune cells and leading to diverse outcomes. Consequently, targeting m6A could be considered for cancer immunotherapy.

## 6 M6A MODIFICATIONS IN CANCER‐ASSOCIATED IMMUNE CELLS

**FIGURE 4:**
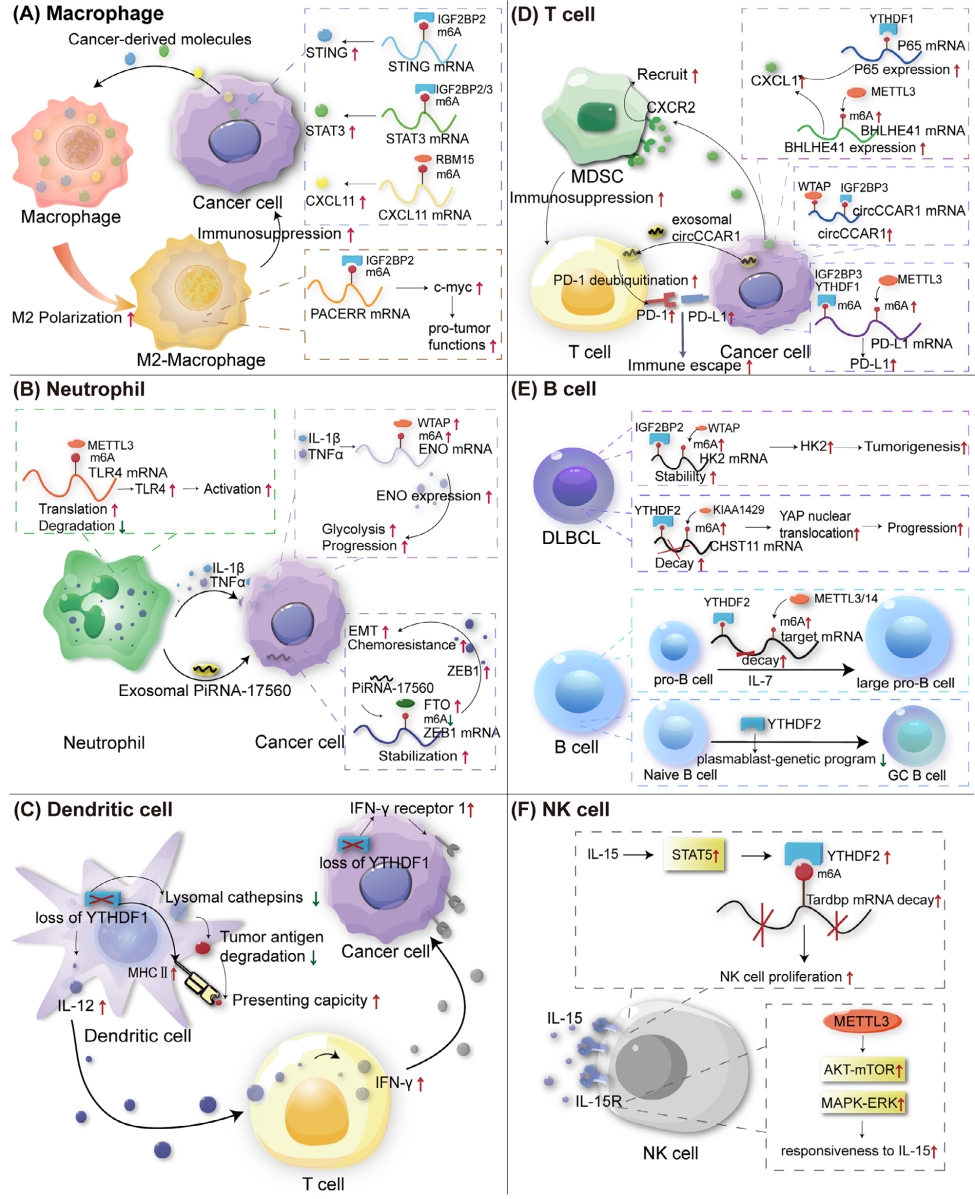
m6A modifications in cancer‐associated immune cells. (A) Macrophages. Within cancer cells, m6A modification affects the expression and secretion of several crucial cytokines and signaling pathways such as IGFE2BP2–STING, IGE2BP2/3–STAT3, and RBM15–CXC11. These cancer‐derived molecules can promote M2‐macrophage polarization. Specifically, in M2‐macrophage, IGF2BP2 promotes PACERR expression and upregulates c‐myc via m6A modification, leading to pro‐tumor functions. (B) Neutrophils. Neutrophil activation is induced by METLL3, leading to increased TLR4 expression. Meanwhile, neutrophil‐derived factors can regulate cancer cell via m6A. Neutrophil‐derived IL‐1 β and TNFα upregulate WTAP expression and ENO expression, promoting glycolysis and cancer cell progression. Neutrophil‐derived exosomal PiRNA‐17560 upregulates FTO and downregulates m6A methylation of ZEB1, which promotes cancer cell EMT and chemoresistance. (C) Dendritic cells. Loss of YTHDF1 in dendritic cells enhances MHCIIexpression and promotes tumor antigen‐presenting ability. At the same time, the absence of YTHDF1 upregulates IL‐12 expression, which in turn boosts INF‐γ expression of T cell. In cancer cells, the deficiency of YTHDF1 also leads to increased expression of INF‐γ receptor 1. The binding of INF‐γ and its receptor triggers an immune response against cancer. (D) T cells. METTL3 enhances BHLHE41 expression in cancer cells, while YTHDF1 upregulates P65 expression. Subsequently, BHLHE41 and P65 can promote CXCL1 expression, which then binds to CXCR2 on MDSC to suppress T cell function. WTAP and IGF2BP3 regulate cancer cell‐derived exosomal circCCAR1 formation, which upregulates PD‐1 expression of T cells by facilitating PD‐1 deubiquitination. Meanwhile, METTL3, IGF2BP3, and YTHDF1 upregulate PD‐L1 expression in cancer cell. Binding of PD‐1 and PD‐L1 promotes immune escape. (E) B cells. METTL3/14 and YTHDF2 destabilize target mRNA to promote IL‐7 dependent transition from pro‐B cell to large pro‐B cell. YTHDF2 also affects transition from naïve B cell to germinal center B cell. Additionally, elevated WTAP stabilizes its target mRNA of hexokinase 2 (HK2), which promotes tumorigenesis of DLBCL. KIAA1429 and YTHDF2 promote YAP nuclear translocation to induce DLBCL progression. (F) NK cells. IL‐15 is a key factor of NK cells. Within NK cells, METTL3 enhances the responsiveness of NK cells to IL‐15. The binding of IL‐15 and its receptor triggers STAT5 activation, which upregulates YTHDF2 and promotes Tardbp mRNA decay to promote NK cell proliferation.

Previously, we analyzed multiple PTMs of m6A regulators and m6A modifications in cancer cells. However, the TME is dynamic and complex, including not only cancer cells but also noncancer cells, including various immune cells that play an essential and frontline role in fighting against viruses, bacteria, and cancer cells by triggering innate and adoptive immune responses.^188^ Dysregulation and dysfunction of immune cells participate in cancer initiation, progression, invasion, and metastasis, as well as immunotherapy resistance.^189^, ^190^, ^191^ Emerging evidence has shown that m6A plays an important role in the growth, differentiation, polarization, migration, and activation of immune cells. Here, we summarized the role of m6A in several cancer‐related immune cells, including macrophages, neutrophils, DCs, T cells, B cells, and NK cells (Figure 4 and Table 4).

**TABLE 4:** m6A modifications in cancer‐associated immune cells.

Cell	Cancer	Function	References
Mø	Glioma	ALKBH5 expression recruits M2 macrophages in glioma to promote cancer proliferation, migration, and invasion.	198
	PC	LncRNA–PACERR interacts with miR‐671‐3p and IGF2BP2 to promote M2 polarization to promote cancer proliferation, migration, and invasion.	199
	HCC	ALKBH5 promotes JNK and ERK pathways via upregulating MAP3K8 to recruit PD‐1+ macrophages by expressing IL‐8 to promote cancer growth and metastasis.	202
	CRC	CircASPH interacts with IGF2BP2 and promotes its stability to activates STING signaling pathway, leading to M2‐TAMs infiltration in CRC.	201
	ccRCC	RBM15 enhances the stability of CXCL11 mRNA and upregulates its expression in an m6A‐dependent manner to promote M2 polarization and infiltration, leading to cancer growth and metastasis.	203
	BC	LINC00657 activates TGF‐β signaling pathway and induces M2‐TAMs infiltration by m6A modification in BC.	204
NE	PTC	METTL3 alters IL‐8 expression to recruit TANs via c‐Rel and RelA inactivation of NF‐κB to suppress cancer progression.	207
	BC	IL‐1β and TNFα secreted from C5aR1+ neutrophils upregulate WTAP expression and m6A levels via ERK1/2 to increase ENO1, leading to promote cancer glycolysis.	119
	BC	Senescent neutrophils‐derived exosomal piRNA‐17560 enhances FTO and decrease m6A levels to strengthen ZEB1 transcripts stability and expression leading to chemoresistance and EMT in cancer cells.	208
DC	cancers	YTHDF1 promotes lysosomal proteases and suppress cross‐presentation of engulfed tumor neoantigens, inducing immune escape.	224
	GC	YTHDF1 loss in GC promotes recruitment of mature DCs with increased MHCII expression and IL‐12 secretion and upregulates expression of IFN‐γ receptor 1 and JAK/STAT1 signaling pathway to restore sensitivity to antitumor immunity.	225
T	NSCLC	METTL3‐modified circIGF2BP3 upregulates PKP3 expression and induce PD‐L1 expression by deubiquitination to inhibit CD8+ T‐cell anticancer efficacy.	234
	BC	METTL3/IGF2BP3 axis upregulates m6A levels of PD‐L1 mRNA to increase its expression, dampening CD8+ T cell response.	235
	CRC	METTL14 loss of TAMs promotes CD8+ T cell dysfunction and promote cancer progression.	236
	CRC	METTL3 activates the m6A–BHLHE41–CXCL1 axis to increase MDSC accumulation and decrease CD8+ T cells.	237
	CRC	YTHDF1 suppress CD8+ T cell infiltration and dampens anticancer immunity via an m6A‐p65–CXCL1/CXCR2 axis to promote CRC.	238
	CRC	KRT17 promotes T cell infiltration via YTHDF2–CXCL10 axis in CRC to control cancer growth.	102
	HCC	WTAP‐mediated circCCAR1 promotes CD8 + T‐cell dysfunction and anti‐PD1 resistance to promote cancer growth and metastasis.	239
	CRC	m6A modification levels increase translation of immune checkpoints and restrain CD8+ T cell function and infiltration to promote cancer growth and immune escape.	240
	BLCA	YTHDF2 regulates RIG‐I‐mediated innate immune and decrease CD8+ T cell recruitment to promote cancer progression and immune evasion.	241
B	DLBCL	METTL3 upregulates pigment epithelium‐derived factor (PEDF) expression to promote proliferation of DLBCL.	253
	DLBCL	NCBP1‐enhanced METTL3 regulates c‐MYC expression via NCBP1/METTL3/m6A/c‐MYC axis to promote DLBCL progression.	254
	DLBCL	KIAA1429 regulates the m6A modification of its downstream CHST11 and Hippo‐YAP pathway to promote DLBCL progression.	255
NK	Cancers	YTHDF2 regulates NK cell function and proliferation by forming a STAT5–YTHDF2 positive feedback loop.	266
	Cancers	METTL3 modifies m6A methylation of SHP‐2 and regulates NK cell response to IL‐15 associated with AKT and MAPK signaling pathway in order to affect anticancer immunity.	267, 268

*Abbreviatios*: Mø, macrophage; NE, neutrophil; DC, dendritic cell; NK, natural killer cell; PC, pancreatic cancer; BC, breast cancer; GC, gastric cancer; NSCLC, non‐small cell lung cancer; CRC, colorectal cancer; HCC, hepatocellular carcinoma; BLCA, bladder carcinoma; DLBCL, diffuse large B‐cell lymphoma.

### 6.1 Macrophages

Macrophages, derived from the mononuclear phagocyte system, play essential roles in various biological and pathological processes.^192^ Macrophages are commonly divided into M1 and M2 types. M1 macrophages are usually considered to have antitumor functions, whereas M2 macrophages are thought to have protumor functions. Additionally, tumor‐associated macrophages (TAMs) significantly influence various stages of cancer progression, including angiogenesis, tumorigenesis, metastasis, invasion, and hypoxia induction, by modulating the tumor tissues.^193^, ^194^, ^195^ m6A RNA modification is a regulator of macrophage activation.^196^ METTL3‐deficient TAMs eventually lead to tumorigenesis by increasing the infiltration of regulatory T cells (Tregs) and reducing the number of Th1 and CD8+ cells through the NF‐κB and STAT3 signaling pathways, resulting in the reduced therapeutic efficacy of anti‐PD‐1 therapy.^197^ A previous study showed that silencing ALKBH5 significantly reduces the infiltration of M2 macrophages in gliomas, suggesting that ALKBH5 may be a potent predictor of sensitivity to immunotherapy in some cancers, especially gliomas.^198^ Liu et al.^199^ found that lncRNA–PACERR increases the number of M2 TAMs in pancreatic cancer (PC) cells in an IGF2BP2/m6A manner. IGF2BP3 upregulates and activates the STAT3 pathway to promote M2‐TAM polarization and immunosuppression in gallbladder cancer.^200^ CircASPH interacts with IGF2BP2, stabilizes it to activate STING signaling, and promotes M2‐TAM infiltration in CRC.^201^ In hepatoma tissues, M2 macrophages exhibit high PD‐L1 expression. ALKBH5 recruits PD‐L1+ TAMs by regulating MAP3K8 expression and activating the ERK/JNK and IL‐8 pathways to promote HCC progression.^202^ RBM15, an oncogene in a number of tumors, enhances the stability of CXCL11 mRNA via m6A RNA modification to facilitate M2 macrophage polarization and infiltration, boosting the progression of ccRCC.^203^ LINC00657 activates the transforming growth factor‐β (TGF‐β) signaling pathway and induces M2‐TAM infiltration by m6A modification in BC.^204^ In conclusion, m6A plays a crucial role in the activation, polarization, migration, and infiltration. Targeting m6A in TAMs may alter the suppressive immune microenvironment to promote anticancer immunity.

### 6.2 Neutrophils

Neutrophils, which account for the majority of granular leukocytes, play essential roles in tumorigenesis, progression, and invasion. Tumor‐associated neutrophils (TANs) influence the activation and function of other immune cells.^205^ The m6A modification plays a crucial role in neutrophil activation. METTL3 enhances m6A methylation, thereby increasing the translation of toll‐like receptor 4 (TLR4) mRNA, a key factor in neutrophil activation.^206^ The m6A modification also influences neutrophil infiltration and migration. In papillary thyroid cancer (PTC), METTL3 deficiency leads to increased IL‐8 expression, which recruits TANs and promotes PTC progression through c‐Rel and Rel A inactivation of the NF‐κB pathway, highlighting the anticancer function of METTL3 in PTC.^207^ piRNA‐17560 increases FTO levels in BC cells by enhancing its stability. Senescent neutrophils are abundant in therapy‐treated tissues and can produce exosomal piRNA‐17560, contributing to chemoresistance and epithelial–mesenchymal transition (EMT) in BC cells in an m6A‐dependent manner.^208^ In addition, Ou et al.^119^ revealed a correlation between m6A and TANs in BC, identifying a novel subset of C5aR1‐positive neutrophils implicated in promoting BC progression. Mechanistically, C5aR1‐positive neutrophil‐derived IL1β and TNFα can activate ERK1/2 signaling, phosphorylating and stabilizing WTAP to promote BC cells by facilitating glycolysis in BC cells.^119^ Solute carrier family 2 member 1 (SLC2A1) plays an essential role in cellular glycometabolism. A pancancer analysis identified SLC2A1 as a m6A‐related potential biomarker for prognosis and immunotherapy. SLC2A1 positively correlates with neutrophils, providing a new strategy for cancer immunotherapy.^209^ Neutrophil extracellular traps (NETs) are extracellular fibrous structures produced by neutrophils that regulate NETosis.^210^, ^211^, ^212^, ^213^ NETs can impair autophagic flux, resulting in abnormal autophagy, which induces sepsis‐associated acute lung injury via METTL3.^214^ Researchers have found that METTL5 is strongly and positively associated with immune cell infiltration, including neutrophil infiltration, in HCC.^215^ Recent studies have demonstrated that NETs are associated with m6A modifications in sepsis‐associated acute lung injury. NETs mediate m6A modification through METTL3, subsequently inducing ferroptosis inacute lung injury.^216^ NETs also participate in cancer invasion, evasion, angiogenesis, and metastasis by regulating the TME.^217^, ^218^, ^219^, ^220^ However, the association between m6A and NETs remains unclear. These studies indicate that m6A participates in TAN activation and infiltration of TANs. Understanding the link between m6A and NETs is a potential direction for future research.

### 6.3 Dendritic cells

DCs, known for dendritic pseudopodia, were first discovered by Steinman in 1973.^221^ DCs are regarded as the most professional antigen‐presenting cells (APCs); they play a key role in anticancer immunity and therapy.^222^ For instance, DCs are necessary for T cell‐mediated antitumor immunity by activating T cells and presenting tumor antigens.^223^ Recently, m6A modification‐mediated DCs and cancer cells have been subjected to new trials. YTHDF1 can increase the expression of lysosomal cathepsins in DCs. Downregulation of cathepsins enhances the cross‐presenting ability of wild‐type DCs. Moreover, the efficacy of PD‐L1 therapy is promoted in Ythdf1^−/−^ mice, indicating that m6A modification and YTHDF1 can modulate anticancer immunity in DCs.^224^ Similarly, in gastric tumors, the loss of YTHDF1 can recruit mature DCs, which subsequently promote sensitivity to antitumor effect.^225^ In HCC, Wang et al.^215^ showed that METTL5 expression was positively correlated with the infiltration of immune cells, including DCs. Gong et al.^226^ demonstrated that the expression levels of METTL14, ZC3H13, and APC (an antagonist of the Wnt signaling pathway) positively correlated with DCs in BC. Downregulation of METTL14 and ZC3H13 correlated with a poor prognosis. Endothelin‐converting enzyme 2 is a prognostic biomarker associated with m6A modifications in lung adenocarcinoma. Endothelial‐converting enzyme 2 expression was significantly negatively correlated with DC infiltration.^227^ Glycolipid transfer protein expression is associated with m6A‐related genes and DCs in cervical cancer (CC).^228^ YTHDC2 is correlated with immune infiltration levels of DCs in head and neck squamous cell carcinoma (HNSCC).^229^ Briefly, m6A modification influences DC activation and infiltration. More importantly, as the strongest APC, targeting the m6A modification of DCs may shed new light on DC‐based immunotherapy.

### 6.4 T cells

T lymphocytes (T cells) play a major role in adaptive cellular immunity and participate in humoral immunity induced by thymus‐dependent antigens.^230^ Functionally, T cells are divided into helper T cells (Ths), cytotoxic T lymphocytes (CTLs), Tregs, and exhausted T cells (Texs).^230^, ^231^ T cells are important for anticancer immunity. Recent studies have shown that the growth and activation of T cells are modulated by m6A RNA modification.^232^, ^233^ For instance, m6A‐modified circIGF2BP3 can inhibit the CD8+ T cell response and induce cancer escape via PD‐L1 deubiquitination in NSCLC.^234^ In BC, the upregulation of the m6A modification of PD‐L1 mRNA via the METTL3/IGF2BP3 axis can downregulate T cell antitumor immune activation to inhibit tumor immune surveillance.^235^ Dong et al.^236^ demonstrated a negative relationship between m6A levels and dysfunctional CD8+ T cells in CRC. METTL3 methylates BHLHE41 mRNA and upregulates its expression, leading to increased CXCL1 expression, which, in turn, recruits immunosuppressive myeloid‐derived suppressor cells (MDSCs) to dampen T cells. Additionally, silencing METTL3 in CRC sustains the activation and proliferation of both CD4+ and CD8+ T cells, thereby suppressing tumor progression.^237^ YTHDF1 in CRC also recruits MDSCs by activating the m6A–p65–CXCL1 axis to inhibit T‐cells, subsequently promoting CRC. This implies that targeting FTHDF1 is a good strategy for boosting anti‐PD1 therapy.^238^ Keratin 17 (KRT17) plays a protective role in CRC by promoting T cell infiltration in a YTHDF2 dependent manner.^102^ In HCC, CD8+ T cell dysfunction can lead to immune evasion. HCC cell‐derived exosomal circCCAR1 is stabilized by WTAP and IGF2BP3, which can be taken up by CD8+ T‐cells. It stabilizes PD‐1 by promoting its deubiquitination. At the same time, it facilitates the dysfunction of CD8+ T cells, resulting in immunosuppression.^239^ Intriguingly, Li et al. found that methionine restriction reduces tumor growth and enhances antitumor immunity by increasing the number and cytotoxicity of tumor‐infiltrating CD8+ T cells in a YTHDF1‐dependent manner, suggesting that targeting methionine metabolism or YTHDF1 is a potential target for tumor immunotherapy.^240^ Zhang et al.^241^ showed that YTHDF2 inhibits its downstream target RIG‐I, thereby facilitating immune evasion in bladder carcinoma (BLCA). YTHDF2‐deficient BLCA cells implanted in recipient mice activated innate immune responses and recruited CD8+ T cells.^241^ HNRNPC interacts with m6A modifications during the immune processes. Cheng et al.^242^ suggested that this gene could enhance the activation of Tregs, leading to immune escape and poor prognosis in prostate cancer. Additionally, some studies have revealed a crosstalk between m6A modification and T‐cell exhaustion in anticancer immunity and immunotherapy. The inhibition of METTL3 or IGF2BP3 can enhance antitumor immunity through PD‐L1‐mediated T‐cell exhaustion in BC as well.^235^ A previous study reported that lncRNA–AC026356.1, a downstream target of METTL14/IGF2BP2, is positively correlated with T cell exhaustion in lung adenocarcinoma.^243^ CCL8 andIL‐1b can make hypoxia zones to recruit TAMs and cytotoxic T cells. The recruited immune cells can then be reprogrammed for immunosuppression in GBM.^244^ This study illustrates a new mechanism of hypoxia in tumors. Previous studies have also reported a correlation between hypoxia and m6A modification.^187^, ^245^ In conclusion, m6A participates in T cell‐mediated antitumor immunity mostly by regulating T cell activation and PD‐1/PD‐L1. Therefore, Tregs and Texs are potential targets for future immunotherapy.

### 6.5 B cells

B lymphocytes (B cells), a type of lymphocyte differentiated from mammalian bone marrow lymphoid stem cells, participate in B‐cell‐mediated humoral immunity.^246^, ^247^ m6A is a key factor in the development, differentiation, and function of B cells. METTL3–METTL14 complex‐mediated m6A modification is crucial for IL‐7‐induced pro‐B cell proliferation via YTHDF2.^248^ Loss of METTL14 eventually represses the transition of pre‐B cells from large to small.^248^, ^249^ Interestingly, another study showed that the loss of METTL3 in the pro‐B stage slightly influences B cells in liver fibrosis.^250^ These results indicate that the link between m6A modification and B cell development may be associated with different stages. B‐cell differentiation is important for antibody‐mediated immunity and is determined by transcription factors. YTHDF2 promotes the formation of germinal centers by suppressing the plasmablast genetic program.^251^ Several B cell lymphomas are associated with m6A modifications. piRNA‐30473 upregulates WTAP expression. Increased WTAP stabilizes its target mRNA, hexokinase 2 (HK2), which promotes tumorigenesis in diffuse large B‐cell lymphoma (DLBCL).^252^ METTL3 is also functionally upregulated in DLBCL tissues.^253^ Furthermore, Meng et al.^254^ verified that NCBP1 enhances METTL3 by maintaining METTL3 mRNA stabilization and mediating c‐MYC to promote DLBCL proliferation. Chen et al.^255^ found that KIAA1429/YTHDF2 suppression of carbohydrate sulfotransferase 11 (CHST11) inactivates the Hippo– yes associated protein (YAP) pathway via m6A RNA modification in DLBCL. Overall, m6A plays key roles in the development, differentiation, and function of B cells. In addition, m6A modification occurs in B cell lymphomas, especially DLBCL, thus providing an avenue to better understand and treat these malignancies.

### 6.6 NK cells

NK cells are cytotoxic lymphocytes derived from bone marrow lymphoid progenitor cells that play a vital role in nonspecific and specific immunity.^256^, ^257^ Unlike T and B cells, NK cells can kill pathogens or cancer cells without prior sensitization.^258^ NK cells have gained attention owing to their heterogeneous characteristics and functions in antitumor immunity and immunotherapy.^259^ Studies have reported alterations in NK cell levels in various tumors, including lung adenocarcinoma,^260^ HCC,^261^, ^262^ bladder cancer,^263^ GC,^264^ and HNSCC.^265^ IL‐15 is an important factor in the proliferation, development, and function of NK cells. Interactions between IL‐15 and m6A regulate the anticancer immunity of NK cells. A previous study reported that the expression level of YTHDF2 is regulated in NK cells. Upregulated YTHDF2 forms a STAT5–YTHDF2 loop that promotes the proliferation and antitumor immunity of NK cells.^266^ METTL3 enhances the NK cell response to IL‐15, which is dependent on the activation of AKT–mTOR and MAPK–ERK. Src homology 2‐containing protein tyrosine phosphatase 2 (SHP‐2), encoded by Ptpn11, is an essential factor in IL‐15‐induced ERK activation. METTL3 deficiency reduces SHP‐2 expression.^267^, ^268^ The relationship between m6A and NK cells is unclear, and further studies are needed to reveal the role of m6A in tumor‐infiltrating NK cells.

TAM heterogeneity is closely associated with tumorigenesis and immune evasion. m6A modification plays an important role in the regulation of immune cell infiltration in the TMEs. Therefore, targeting m6A‐regulated immune cells may be an attractive therapeutic strategy for restoring antitumor immunity.

## 7 M6A MODIFICATIONS IN MICROORGANISM‐ASSOCIATED CANCERS

Recently, microorganisms have been studied for their roles in carcinogenesis and therapeutic responses. Previous studies have shown that microorganisms increase mutagenesis, regulate oncogenic genes, and modulate immunity.^269^, ^270^ Microorganisms may influence tumor onset and progression by modulating m6A modification.^271^ A previous study reported that microbial pathogens can disrupt pulmonary immune homeostasis by altering host m6A modifications, thereby influencing NSCLC development and outcomes.^271^ Therefore, we reviewed the interplay between m6A modifications and specific cancer‐related microorganisms, such as Hp, Fn, hepatitis B virus, Epstein–Barr virus, and human papilloma virus, to provide insights into the epigenetic mechanisms and treatment strategies of microorganism‐associated cancers (Table 5).

**TABLE 5:** m6A modifications in microorganism‐associated cancers.

Pathogen	Cancer	Regulator	Function	References
Hp	GC	FTO	CagA+ Hp increases the expression of FTO, which downregulates the m6A level of CD44 mRNA to promote tumorigenesis.	275
Fn	CRC	METTL3	Fn decreases METTL3 expression and m6A levels of KIF26B to promote CRC progression.	281
	ESCC	METTL3	Fn upregulates the expression of METTL3 to promote ESCC proliferation and metastasis by promoting c‐Myc mRNA methylation.	283
HBV	HCC	YTHDF2	YTHDF2 can promote the expression of MMP2 and MMP5 to promote tumorigenesis and progression of HBV‐associated HCC.	123, 187
	HCC	IGF2BP3	HBV‐pgRNA can upregulate IGF2BP3 expression to promote HCC.	289
	HCC	YTHDC1	YTHDC1 and FMRP can promote the nuclear export of HBV‐related transcripts to influence the life cycle of HBV.	291
	HCC	ALKBH5 WTAP	ALKBH5 interacting with macrophages and WTAP interacting with natural killer T cells may influence the progression of liver fibrosis in HBV infection.	293
EBV	cancers	METTL14	Viral‐encoded latent oncoprotein EBNA3C can bind to METTL14 and promote tumorigenesis.	299
	GC	WTAP	EBER1 regulates can downregulates WTPA expression to promote the migration of EBV‐associated GC.	300
	GC	METTL3	EBV‐circRPMS1 upregulates METTL3 expression to promote the progression of EBV‐associated GC.	302
HPV	CC	IGF2BP2	E6/E7 proteins regulate the expression of MYC via IGF2BP2 to promote CC.	307
	cancers	IGF2BP1	IGF2BP1 can stabilize E7 transcripts to influence HPV‐associated cancers.	308
	CC	ALKBH5	E7 can increase ALKBH5 expression and enhance PAK5 expression to promote tumorigenesis and metastasis of CC.	309
	CC	METTL3	METTL3 inhibitors combined with anti‐PD1 therapy enhance the efficacy of immunotherapy in CC.	311

Abbreviations: GC, gastric cancer; HCC, hepatocellular carcinoma; CRC, colorectal cancer; ESCC, esophageal squamous cell carcinoma; CC, cervical cancer.

### 7.1 Helicobacter pylori

Hp infection is one of the main causes of GC. Typically, Hp infection first induces nonatrophic gastritis, which can develop into atrophic gastritis, gastric polyps, and ultimately, GC.^272^ Mechanistically, a recent review summarized that Hp may impair gastric epithelial cells through oxidative stress, DNA damage, impairment of DNA repair pathways, and endoplasmic reticulum stress.^273^ Recently, Li et al.^274^ performed a comprehensive analysis of differences in m6A modifications during Hp infection. They found an increasing level of m6A in Hp infection and a significantly different expression of m6A regulators, indicating that m6A modification might correlate with Hp infection.^274^ FTO promotes tumorigenesis in chronic CagA + Hp infection by regulating CD44 mRNA m6A methylations.^275^ Additionally, Hp infection has an undesirable effect on cancer immunotherapy by decreasing the efficacy of anti‐PD1 immunotherapy.^276^ Some researchers have suggested that increasing the expression of PD‐L1 could be an early response to Hp infection.^277^ The relationship between Hp infection and host cell DNA impairment has been studied to some extent. However, the relationship between Hp and m6A modifications in gastric carcinogenesis and disease progression remains largely unknown. Further studies are required to provide additional insights into Hp‐related GC treatment.

### 7.2 Fusobacterium nucleatum

Fn is an opportunistic pathogen in human body.^278^ Emerging research has reported that Fn is associated with CRC.^279^, ^280^ Herein, from the perspective of m6A modification, we present illustrative research on Fn and CRC. Fn decreases METTL3 expression and m6A levels in KIF26B by activating YAP signaling and inhibiting FOXD3 (forkhead Box D3, a transcription factor for METTL3) expression to promote CRC progression.^281^ Xu et al.^282^ indicated that Fn infection‐induced microRNA‐4717‐3p excessive maturation via METTL3‐dependent m6A modification suppressed the expression of mitogen‐activated protein kinase 4 and its anticancer function to promote CRC proliferation. Fn not only facilitates CRC, but also correlates with ESCC. Researchers have found that the upregulated expression of METTL3, which is induced by Fn, could promote ESCC proliferation and metastasis by promoting c‐Myc mRNA methylation in a YTHDF1‐dependent manner.^283^ Additionally, Fn‐related m6A modifications are involved in CRC immunotherapy. Fn promote the expression of PD‐L1 and mediate immune escape in CRC via m6A‐modified IFIT1.^284^ In conclusion, Fn is involved in cancer progression and immunotherapy. Further studies are needed to explore strategies for treating cancers, particularly CRC.

### 7.3 Hepatitis B virus

HBV is a member of the Hepadnaviridae family of enveloped viruses, with a double‐stranded DNA genome of 3200 bp in length.^285^ HBV infection is also associated with HCC. In the present study, we clarified the function of m6A modification during this process. First, HBV RNA is predominantly modified by m6A in the coding region of HBx.^286^, ^287^ Kim discovered an m6A site at nt 1616 of the HBV genome, indicating that m6A modification may regulate HBx protein expression to modulate the HBV life cycle.^288^ Yang et al. found that YTHDF2 stabilizes the transcripts of minichromosome maintenance protein 2 (PPM2) and minichromosome maintenance protein 5 (PPM5) via m6A modification, which promotes the tumorigenesis and progression of HBV‐associated HCC.^123^ HBV‐pgRNA (pregenomic RNA) upregulated IGF2BP3 expression to promote HCC. Furthermore, interferon (IFN)‐α−2a can increase pgRNA m6A modification and degrade its stability.^289^ Kim et al. demonstrated that HBV could increase the m6A modification of phosphatase and tensin homolog (PTEN) RNA, leading to decreased RNA stability and PTEN protein expression. PTEN downregulation can affect nonspecific immunity by inhibiting interferon regulatory factor 3 (IRF‐3) nuclear import. Simultaneously, it activates the PI3K/AKT pathway, which influence HCC development.^290^ The m6A modification can also affect HBV RNA localization. YTHDC1 and FMRP promote nuclear export of HBV transcripts. The loss of YTHDC1 and FMRP can inhibit reverse transcription in HBV, affecting the HBV life cycle.^291^ Additionally, the m6A modification modulates immune cell infiltration in HBV‐HCC. In a recent study, patients were divided into two clusters: cluster A and B. Relatively, the overall survival rate of cluster A was higher than that of addition, and immune cell infiltration of the two clusters was significantly different.^292^ Another study pointed out that ALKBH5 interacting with macrophages and WTAP interacting with NK T cells may be key factors in the progression of liver fibrosis during HBV infection.^293^ In summary, the m6A modification regulates HBx RNA and protein levels to modulate the HBV life cycle. HBV infection influences both oncogene expression and immune cell infiltration in an m6A‐dependent manner to regulate HCC progression.

### 7.4 Epstein‒Barr virus

EBV is an oncogenic herpes virus linked to various cancers, including Burkitt's lymphoma, nasopharyngeal carcinoma (NPC), and EBV‐associated GC.^294^ Recent studies have indicated that the m6A modification may be involved in this process. First, m6A modification mediates mRNA decay during EBV lytic reactivation to regulate its life cycle.^295^ The m6A modification can promote EBV lytic reactivation by attenuating IFN signaling.^296^ Additionally, WTAP deposits m6A marks on EBV transcripts and recruits YTHDF reader proteins to activate the CNOT1 RNA decay pathway.^297^ The tumorigenic function of EBV may be related to virus‐encoded latent oncoproteins such as EBNA2.^298^ One study examined the role of EBNA3C, which binds METTL14 to promote tumorigenesis.^299^ In addition, EBER1 can activate the NF‐κB signaling pathway, which downregulates WTPA expression to promote the migration of EBV‐associated GC.^300^ Liu et al.^301^ divided patients with NPC into two subgroups: an m6A high‐score group and an m6A low‐score group. They found that the m6A high‐score group was related to immune suppression and poorer survival, while the m6A low‐score group was related to a better response to immunotherapy; therefore, m6A modification is likely related to NPC progression and immunotherapy effects.^301^ Zhang et al.^302^ demonstrated that EBV‐circRPMS1 promotes EBV‐associated GC progression by recruiting Sam68 to METTL3 and upregulating METTL3 expression. Overall, m6A participates in the lytic reactivation of EBV and its life cycle. The m6A modification may regulate oncoproteins to influence EBV‐associated cancers; however, it remains unclear whether this modification can be applied in EBV‐associated cancer treatment.

### 7.5 Human papillomavirus

HPV is a small double‐stranded DNA virus that infects the squamous epithelia and promotes tumorigenesis.^303^ Globally, 4.5% of cancers are caused by HPV, of which squamous epithelium‐associated cancers account for a large proportion, including CC.^303^, ^304^ HPV is associated with several cancer processes, including initiation, progression, invasion, and metastasis, with unclear mechanisms.^305^ In the present study, we focused on m6A modifications. Sustained expression of HPV E6/E7 oncogenes alters cancer cell growth.^306^ The m6A modification can modulate E6/E7 expression to influence cancer cells. E6/E7 proteins regulate the m6A methylation levels of MYC mRNA in an IGF2BP2‐dependent manner, which promotes several biological and pathological processes in CC, including aerobic glycolysis, proliferation, and metastasis.^307^ Wang et al.^308^ showed that E7 transcripts are stabilized by the m6A reader IGF2BP1. Interestingly, upon heat stress, the m6A‐modified E7 reversed the fate of IGF2BP1. Based on this, they provided a treatment strategy for HPV‐associated cancers that depend on heat.^308^ Huo et al.^309^ found that E7 increases ALKBH5 expression and enhances PAK5 expression by downregulating m6A modification levels, which promotes the tumorigenesis and metastasis of CC. Additionally, the m6A modification influences the antiviral treatment of HPV‐associated cancers. IFN‐ε is a key cytokine that helps the human body defend against viral infection, especially in epithelial cells. HPV can influence the m6A RNA modification of IFNE via WTAP to regulate IFN‐ε, subsequently influencing the nonspecific immune responses to HPV in condyloma acuminata.^310^ Importantly, METTL3 has been identified as a mediator of the immunosuppressive TME in HPV‐associated cancers. In vivo cell‐derived xenograft models showed that METTL3 inhibitors combined with anti‐PD1 therapy enhanced the efficacy of immunotherapy in CC.^311^ Collectively, m6A modifications interact with HPV infections and influence immunotherapy.

Microorganisms regulate the expression of host tumor‐related genes through m6A modifications. However, even after the microorganisms are eradicated, these epigenetic changes can persist and continue to drive tumorigenesis, which is called “the hit‐and‐run model.” Understanding this interaction provides crucial insights into the mechanisms underlying microorganism‐related cancers and highlights potential targets for therapeutic intervention in m6A modification pathways.

## 8 M6A MODIFICATIONS IN CANCER IMMUNOTHERAPY

**FIGURE 5:**
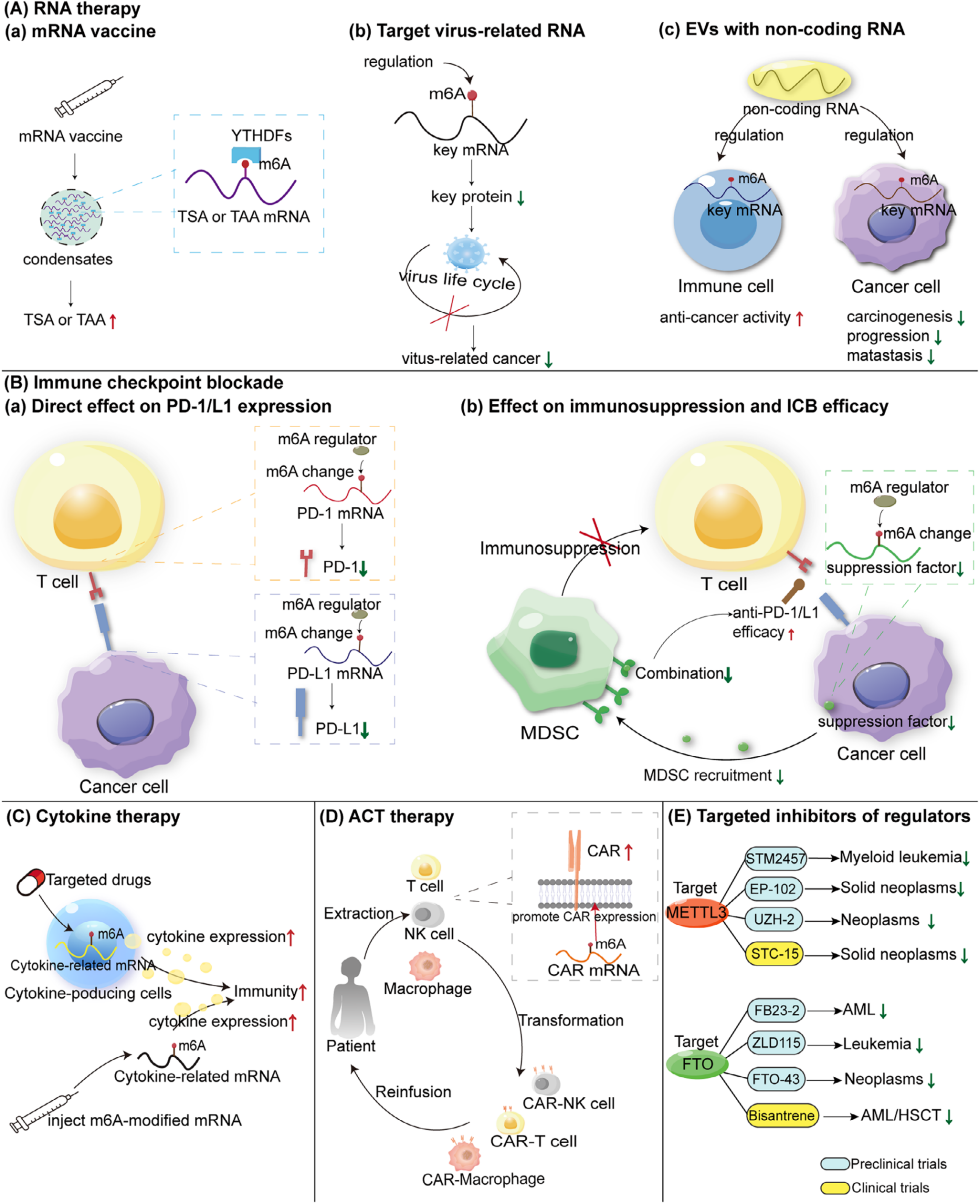
m6A modifications in cancer immunotherapy. (A) RNA therapy. (a) YTHDF family protein can form condensates with m6A‐modified mRNA via LLPS, which may promote TAA or TSA expression of mRNA vaccine. (b) m6A modification can suppress virus‐related cancer by blocking its life style. (c) Noncoding RNA‐loaded EVs can regulate both immune cell and cancer cell m6A modification by regulating key mRNA expression, which promotes anticancer immunity. (B) Immune checkpoint blockade. (a) m6A modification can downregulate PD‐1/PD‐L1 expression to suppress immune escape. (b) m6A modification has the potential to decrease MDSCs recruitment and dampen their function on T cell dysfunction, which relieves immunosuppression and enhances anti‐PD‐1/PD‐L1 therapy efficacy. (C) Cytokine therapy. Both targeting cellular m6A modification and injecting m6A‐modified cytokine‐related mRNA can improve its expression to promote anticancer immunity. (D) Adoptive cell transfer (ACT) therapy. m6A modification plays an important role in improving CAR expression to enhance CAR‐immune cell function. (E) Targeted inhibitors of m6A regulators. For METTL3‐targeted inhibitors, STM2457, EP‐102, UZH‐2 are in preclinical trials and are beneficial in myeloid leukemia, solid neoplasms, and neoplasms, respectively. STC‐15 is in clinical trials and has the potential to treat solid neoplasms. For FTO‐targeted inhibitors, FB23‐2, ZLD115, and FTO‐43 are in preclinical trials for AML, leukemia, and neoplasms, respectively. Bisantrene is in clinical trials for the treatment of AML/HSCT. TAA, tumor‐associated antigens; TSA, tumor‐specific antigens; MDSC, myeloid‐derived suppressor cell; CAR, chimeric antigen receptor; AML, acute myeloid leukemia; HSCT, hematopoietic stem cell transplantation.

Cancer immunotherapy has attracted increasing attention in recent years. Distinct from therapies that directly influence cancer cells or tissues, such as surgery and chemotherapy, immunotherapy aims to enhance the interaction between cancer and immune cells, boosting the immune response and suppress cancer progression.^20^ Additionally, clinical data have demonstrated that patients with high sensitivity to immunotherapy have longer survival rates and decreased recurrence rates, highlighting the significance of immunotherapy.^312^ However, numerous factors, including the limited efficacy of immunotherapy, complicated TME, immune escape, and tumor heterogeneity, have made it difficult for every cancer patient to benefit. Interestingly, the m6A modification may play a role in improving these effects. For instance, the m6A‐associated TME can be analyzed to aid immunotherapy.^313^ Therapeutic resistance is also associated with m6A modification.^314^ Herein, we expound on several advances in cancer immunotherapy from an m6A modification perspective with the hope of providing novel insights into immunotherapy enhancement. RNA therapy, immune checkpoint inhibitors (ICIs), cytokine therapy, ACT therapy, and direct targeting of m6A regulator therapy are mainly included (Figure 5).

### 8.1 RNA therapy

As a carrier of genetic information, RNA plays an essential role in diverse biological processes, as well as in disease processes such as cancer. Specific chemical modifications of RNA directly influence its molecular function.^315^ m6A, the most abundant RNA modification, regulates both mRNA and ncRNA stability, translocation, and translation efficacy to modulate anticancer immunity.^316^, ^317^ Moreover, targeting m6A modifications of both mRNAs and ncRNAs may have potential value in cancer immunotherapy. Thus, we have clarified several recent advances and prospects.

#### 8.1.1 Prospect of cancer mRNA vaccines with m6A modification

With the recent application of COVID‐19 mRNA vaccines, such as the BNT162b2 vaccine, mRNA vaccines have been extensively studied over the last 5 years.^318^ In addition to infectious diseases, the potent preventive and therapeutic value of mRNA vaccines in oncology is unclear and attractive. Several clinical trials are ongoing to test its efficacy and safety, including NCT04534205 and NCT03313778.^319^ In contrast to other types of cancer vaccines, including peptide, protein, and cellular vaccines, mRNA vaccines have a more persistent expression of tumor‐associated antigens or tumor‐specific antigens (TAAs or TSAs) and a lower risk of human genome alteration caused by injected nucleotide sequences than DNA vaccines.^320^, ^321^ However, a few challenges restrict the development of mRNA vaccines, including the balance of antigen expression and adjuvant effects, stability and safety, appropriate delivery methods for vaccines, and targeted tumor antigen mutation.^322^ Here, we provide insights into the m6A modifications.

The m6A modification may enhance mRNA vaccine expression, which is a crucial aspect of mRNA vaccine function. Katalin Karikó and Drew Weissman, who were awarded the 2023 Nobel Prize in Medicine or Physiology, discovered in 2005 that modified nucleosides such as m5C, m6A, m5U, s2U, and pseudouridine can modulate mRNA activity. In particular, pseudouridine has been shown to reduce mRNA degradation and increase protein expression, forming the basis for COVID‐19 mRNA vaccines.^323^ These results highlighted the crucial role of RNA modification in the development of mRNA vaccines. Modification of the 3ʹ‐UTR improves mRNA vaccine stability and translation efficiency.^324^ Recent findings by He et al.^325^ suggested that m6A modification was predominantly found in the 3′‐UTR and coding sequences (CDS). Future research should explore how altering m6A modifications affects mRNA vaccine stability and translation efficacy. In addition, m6A modification may facilitate mRNA vaccine activation via LLPS, a process that forms cellular membraneless components (e.g., biomolecular condensates, including the P‐body, ribosomes, stress granules, and autophagosomes).^326^ LLPS is involved in the processes of cancer.^327^ Emerging evidence has shown that m6A promotes the formation of transcriptional condensates, thereby enhancing gene expression.^328^ This relationship may offer a novel approach for mRNA vaccine development. Although the mechanisms of LLPS remain largely unknown, IDRs are widely regarded to correlate with condensate formation.^329^ Lee et al.^328^ found that arginine residues in YTHDC1 IDR2 are important for condensate formation, which promotes gene activation. Additionally, Chen et al.^330^ reported that YTHDC1 undergoes LLPS and forms nuclear YTHDC1–m6A condensates, which maintain mRNA stability and control myeloid leukemic differentiation. YTHDF1, another YTH domain‐containing protein, targets mRNAs for degradation by promoting P‐body formation via LLPS.^331^ These studies indicate that m6A modifications may affect mRNA translation, stability, and activation via LLPS. The potential of modulating condensate formation to enhance mRNA vaccine efficacy, particularly in stabilizing and activating tumor antigen‐related mRNA, remains an intriguing area for future research.

#### 8.1.2 Targeting viral RNA m6A modification to treat virus‐associated cancer

Viral infection is the first step in virus‐induced cancer. Persistent and chronic infections cause inflammation, eventually leading to cancer initiation. Therefore, the inhibition of viral action and stimuli is important. Recent studies have shown that m6A modifications may shed new light on antiviral treatments at the epigenetic level. For example, the HBV vaccine has been widely used to prevent HBV infection, whereas therapeutic vaccines exhibit limited efficacy and weaken immune responses, especially in chronic HBV infection, which may progress to HBV‐associated HCC.^332^ Moreover, HBV is often reactivated after discontinuation of nucleoside analogs that target HBV DNA replication.^333^ Future studies should focus on interrupting HBV RNA expression. Kim et al. identified m6A at nt 1616 in the coding region of HBx, which regulates HBx protein expression. After silencing YTHDF2 and methyltransferases, both HBx RNA and HBx protein expression levels were notably increased.^288^ This study suggests that m6A modification could be a novel target for treating HBV‐associated HCC. During HPV infection, the HPV E7 oncotranscripts confer thermal vulnerability through IGF2BP1‐dependent m6A modifications. Heat stress induces the formation of distinct m6A‐modified E7 mRNA–IGF2BP1 granules, which can be resolved by the ubiquitin–proteasome system. This provides a potential heat treatment strategy for HPV‐associated cancers.^308^ In summary, targeting viral RNA and inhibiting viral morbigenous protein expression by m6A modification provides some clues. However, several issues remain unresolved. First, its application in treatment remains elusive, and clinical trials should be performed to determine whether altering m6A modifications can efficiently alter viral RNA expression in patients. Second, appropriate drugs targeting viral RNA modifications are expensive. Third, the mechanisms of targeting RNA m6A modifications in other viruses, including bacteria, fungi, and other pathogenic microorganisms, which may lead to cancer, need to be explored.

#### 8.1.3 m6A and extracellular vesicles loaded with ncRNA

Thus, m6A has the potential to be a target for tumor immunotherapy. Extracellular vesicles (EVs) have been engineered as carriers of ncRNA, which play a vital role in both immune cells and cancer cells.^334^ These ncRNAs target m6A regulators and regulate the m6A modification of specific mRNAs to modulate several physiological or pathological processes. Cigarette smoking induces M2‐TAMs to secrete EVs carrying circEML4. These EVs are transported into NSCLC cells, where circEML4 reduces ALKBH5 levels and increases the suppression of cytokine signaling 2 (SOCS2) mRNA m6A modification in the nucleus, resulting in decreased SOCS2 expression and an activated JAK–STAT signaling pathway. Consequently, EVs promote the progression, migration, invasion and metastasis.^335^ This study explains the mechanism by which cigarette smoking induces NSCLC from epigenetic and immunological perspectives. Importantly, we have developed a new hypothesis regarding NSCLC treatment by cutting off the transportation of circEML4‐loaded vesicles from M2‐TAMs to NSCLC cells. You et al.^336^ constructed EVs with high CD47 expression, which were derived from macrophages and loaded with short interfering RNA against YTHDF1 to treat GC by self‐presentation of immunogenic tumors and blockade of CD47. Notably, these particles can preferably interact with regulatory protein α, which can help TAMs to kill cancer cells with more effective delivery and lower toxicity.^336^ Recently, exosomes with a diameter of approximately 100 nm have attracted increasing amount of attention.^337^ The lncRNA MiR4458HG derived from HCC can be packaged within exosomes to promote M2‐TAM polarization by increasing ARG1 expression. Additionally, these EVs increase IGF2BP2 expression in HCC cells, which stabilizes SLC2A1 and HK2 mRNA to promote HCC progression.^338^ Exosomal circVMP1 upregulates METTL3 expression and m6A modification of SOX2 to facilitate NSCLC progression.^339^ Conversely, m6A modifications mediate the formation of ncRNA‐loaded EVs. In HCC cells, WTAP and IGF2BP3 stabilized circCCAR1 and circCCAR1‐loaded exosome formation. These exosomes can be taken up by CD8+ T cells, where circCCAR1 promotes PD‐1 deubiquitination and stability, ultimately promoting CD8+ T cell dysfunction and HCC immune escape.^239^ Similar results have been previously reported. For instance, the formation of adipocyte exosomes carrying lncRNAs LOC606724 and SNHG1 is promoted by METTL7A in an m6A‐dependent manner.^28^ In addition, ncRNA vaccines have been studied in recent years. Li et al.^340^ developed a circRNA vaccine that drives immunity in hard‐to‐treat malignancies. Despite the promise of circRNA vaccines, their low immunogenicity and insufficient pro‐inflammatory microenvironment remain significant challenges. From an m6A perspective, we propose two solutions: first, m6A modifications may stabilize circRNAs and enhance their functionality; second, EVs with m6A modifications could serve as effective delivery carriers, facilitating the entry of circRNA vaccines into cells. These hypotheses warrant further investigation. In conclusion, EVs loaded with ncRNAs play crucial roles in cancer progression, anticancer immunity, and immunotherapy. Future research may focus on the application of m6A modifications and EVs. Specifically engineered EVs can be constructed to modulate the m6A modification of key target mRNAs, thereby regulating cancer cell processes and immune cell functions. Additionally, m6A modification of ncRNAs can enhance the formation and function of critical EVs in target cells. The potential use of EVs with m6A modifications as carriers for ncRNA vaccines is also worth exploring.

### 8.2 Immune checkpoint blockade

ICB has been widely applied in diverse diseases, especially cancer, wherein ICIs block the interaction between receptors on the surfaces of immune and tumor cells, thereby inhibiting the dysfunction of immune cells and enhancing their anticancer immunity.^341^ The use of ICIs has significantly prolonged the survival of many cancer patients. Current evidence suggests that m6A modifications closely influence immune checkpoint‐blocking therapy by directly regulating immune checkpoint expression and modulating immune cell suppression. This reduces drug resistance and enhances treatment efficacy.

#### 8.2.1 The role of m6A in anti‐PD‐1/PD‐L1 therapy

Anti‐PD‐1/PD‐L1 therapy is the most prevalent immune checkpoint‐blocking therapy. PD‐1 (programmed cell death protein‐1, also called CD279) is an important immunosuppressive molecule that is mainly expressed on the surface of immune cells such as macrophages. PD‐1 can downregulate functions of the human immune system.^342^, ^343^ PD‐L1, also regarded as a ligand of PD‐1, also called CD274. It is mainly expressed in tumors and tumor‐related cells.^344^ The PD‐1/PD‐L1 axis inhibits cytotoxic T cell‐mediated tumor responses to induce immune escape.^345^ Recent studies have reported the function of m6A in PD‐1/PD‐L1 immunotherapy.

m6A modification regulates PD‐1/PD‐L1 expression, thereby regulating PD‐1/PD‐L1‐related immune evasion by directly affecting related mRNA stability or influencing its modification. Researchers have found that circIGF2BP3 can upregulate the expression of PKP3, which stabilizes OTUB1 mRNA and thus promotes PD‐L1 deubiquitination, ultimately increasing the expression of PD‐L1 and simultaneously promoting immune evasion in NSCLC.^234^ METTL14 upregulates lncRNA MIR155HG m6A modification and its stability, which promotes the expression of PD‐L1, leading to immune escape in HCC.^346^ ALKBH5 stabilizes its target ZDHHC3 mRNA, which in turn stabilizes PD‐L1 to promote immune escape from glioma.^347^ METTL3 upregulates PD‐L1 expression by modifying lncRNA MALAT1, which promotes the progression and immune evasion of PC.^348^ METTL16 can increase the expression of PD‐L1 in CRC.^349^ ALKBH5 orchestrates PD‐1 expression in intrahepatic cholangiocarcinoma (ICC) and colon adenocarcinoma, with strong expression of ALKBH5 leading to high sensitivity to anti‐PD1 immunity in tumors.^350^, ^351^ These studies demonstrated that m6A modification promotes PD‐1/PD‐L1‐related cancer immune evasion by regulating its expression. Targeting the functions of m6A regulators or m6A sites is a potential therapeutic strategy for cancer immunotherapy. Regulation of m6A modification may alter cancer immune evasion and improve the treatment efficacy of the traditional anti‐PD‐1/PD‐L1 strategy by regulating PD‐1/PD‐L1 expression. For instance, IOX1, a specific ALKBH5 inhibitor, significantly decreases PD‐L1 expression and prolongs survival in mice.^347^ In summary, targeting m6A modifications to regulate PD‐1/PD‐L1 expression to reduce immune evasion and improve immunotherapy is valuable.

Similar to chemotherapy, drug resistance is a significant challenge in immunotherapy, driven by multiple underlying mechanisms.^352^ One such mechanism is the link between epigenetics and immune cell dysfunction, which is the focus of our discussion. Targeting m6A modifications can reduce the immunosuppressive state of immune cells and promote their infiltration, thereby alleviating drug resistance and enhancing anti‐PD‐1/PD‐L1 therapy. Wang et al.^353^ found that the depletion of METTL3 and METTL14 enhances responses to anti‐PD‐1 therapy in colorectal cancer by stabilizing Stat1 and Irf1 mRNA and promoting IFN‐γ–Stat1–Irf1 signaling via YTHDF2. Metabolic stress and starvation can increase FTO expression in melanoma cells, which eventually promotes proliferation, migration, and evasion of melanoma cells, as well as increases anti‐PD‐1 resistance.^354^ YTHDF1, which is of great significance, promotes colorectal cancer via an m6A–p65–CXCL1/CXCR2 axis.^238^ Interestingly, researchers have also reported that high YTHDF1 expression is related to a better prognostic outcome in patients with NSCLC because of better infiltration of lymphocytes and downregulation of PD‐L1.^51^ This study provides researchers with a new approach to enhance anti‐PD‐1 efficacy in NSCLC. The METTL3 inhibitor STM2743 downregulates the m6A methylation of BHLHE41 and the expression of CXCL1, thereby decreasing MDSC migration. Downregulated MDSC migration dampens their suppressive function in CD8+ T cells; as a result, it improves anti‐PD‐1 therapy in CRC.^237^ Similarly, Bao et al.^238^ found that targeting YTHDF1 with siYTHDF1 suppresses its binding to m6A‐modified p65 mRNA and decreased CXCL1 expression, leading to reduced MDSC migration and better anti‐PD‐1 efficacy. KRT17 promotes YTHDF2 degradation. Degradation of YTHDF2 decreases the decay of m6A‐modified CXCL10 mRNA and increases its expression, promoting cytotoxic T lymphocyte (CTL) infiltration into the tumor tissue. KRT17 synergized with anti‐PD‐1 and showed satisfactory efficacy in CRC.^102^ Inhibition of ALKBH5 downregulates Dickkopf‐related protein 1 (DKK1) expression via the Wnt/β‐catenin pathway, subsequently leading to less MDSC recruitment and better anti‐PD‐1 outcomes in CRC.^355^ In nonalcoholic fatty liver disease‐related HCC (NAFLD‐HCC), METTL3 increases SCAP mRNA translation in an m6A‐dependent manner, which induces cholesterol production and inactivates CD8+ T cells. Inhibition of METTL3, including the small‐molecule inhibitor STM2457 and the nanoparticle siMETTL3, can effectively restore CD8+ T‐cell function and boost anti‐PD‐1 treatment.^356^ In conclusion, the m6A modification is a potent target for the reversal of immunosuppression and restoration of immune cell function, leading to a decrease in resistance and improvement in the efficacy of anti‐PD‐1/PD‐L1 therapy.

#### 8.2.2 Other immune checkpoints

In addition to PD‐1/PD‐L1, other immune checkpoints, such as CTL‐associated antigen‐4 (CTLA‐4), mitogen‐activated protein kinase, TIM family, and T‐cell Ig and ITIM domains, are of great significance and have potential therapeutic effects.^357^, ^358^, ^359^, ^360^ Therefore, the role of m6A modifications in these immune checkpoints is worth exploring. For example, m6A‐modified circQSOX1 promotes the expression of PGAM1, which induces immune evasion by activating glycolysis and inactivating anti‐CTLA‐4 therapy in CRC.^361^ Thus, Sh‐circQSOX1 synergized with anti‐CTLA‐4 therapy may overcome resistance to anti‐CTLA‐4 treatment. Additional immune checkpoints may play a role in immunotherapy via m6A modification.

### 8.3 m6A modification and cytokine therapy

Cytokines are secreted by both immune and nonimmune cells. Cytokines play a key role in anticancer immunity.^362^, ^363^ Over the past 30 years, cytokines and cytokine receptors have gained increasing attention because of their vital roles in several physiological and pathological processes. Cytokine therapy is a novel cancer immunotherapy. Diverse cytokines have been considered immunotherapeutic targets, such as IL‐1,^364^ IL‐2,^365^ IL‐6,^366^ IL‐8,^367^ and IL‐15.^368^ Here, we reviewed the role of m6A in cancer cytokine immunotherapy. The m6A modification modulates cytokine expression to recruit immune cells. For instance, METTL3 deficiency improves IL‐8 production by PTC cells, which recruit TANs and promotes PTC progression.^207^ Targeting the upregulation of METTL3 and mRNA m6A modifications may reduce IL‐8 secretion and decrease TAN infiltration, thereby enhancing the efficacy of immunotherapy in PTC. YTHDF1 promotes HCC progression by increasing IL‐6 secretion and recruiting MDSCs. Reducing YTHDF1, for instance with lipid nanoparticle‐encapsulated siRNA against YTHDF1 (LNP‐siYTHDF1), can alleviate MDSC‐induced CD8+ T cell exhaustion and improve anticancer immunity.^369^ YTHDF1 loss in GC increases IL‐12 expression and DC recruitment, which restores sensitivity to anticancer immunity.^225^ Cytokines are multifunctional and important biomolecules that participate in anticancer immunity. Cytokine therapy alters the TME and improves interactions between immune and cancer cells. Based on previous studies, the m6A modification may provide a new strategy for cytokine therapy. m6A plays a vital role in cytokine secretion by recruiting tumor‐associated immune cells to influence cancer progression. Future research should target cytokine‐related mRNA m6A modifications in cancer and immune cells to change specific cytokine expression, thus enhancing the efficacy of immunotherapy. In addition, we suggest that constructing and delivering m6A‐modified cytokine‐related mRNAs into the TME to persistently increase or decrease cytokine expression may be helpful in cancer immunotherapy. However, further studies are needed to confirm this hypothesis.

### 8.4 ACT therapy

ACT therapy refers to the modification of immune cells to express a chimeric antigen receptor (CAR) for the treatment of different types of cancers, especially leukemia and lymphoma.^370^, ^371^, ^372^ The CAR consists of an antigen‐binding region, a transmembrane region, and a signal transmembrane region. Typically, CAR immune cells are derived from a patient's peripheral blood, engineered to express CARs in vitro, and reinjected into the patient after expansion. Therefore, CAR immune cells can specifically recognize and interact with cancer cells without antigen processing or presentation.^372^ CAR‐T, CAR‐NK, and CAR‐macrophage therapies have been studied, with CAR‐T therapy being the most abundant.^373^ Recently, scientists reported that super CAR‐T cells target multiple tumor‐associated antigens and exhibit improved antitumor ability.^374^ However, challenges such as primary resistance, relapse, and adverse effects are unsolved.^375^ Although CAR‐T therapy has been successful in treating leukemia and lymphoma, a number of solid tumors cannot obtain satisfactory results, which is probably related to a lack of tumor‐specific targets, immunosuppressive TME, problems with homing and access to the tumor site, and lack of CAR‐T cell expansion.^376^ Interestingly, non‐m6A‐related neoantigen‐coding lncRNAs are regarded as vital factors in glioma progression and may provide an important direction for CAR‐T therapy.^377^ The m6A modification is still worth considering for applications in CAR‐T therapy. First, m6A modification regulates immunosuppressive TME.^378^ Targeting m6A may relieve the immunosuppressive TME and enhance the efficacy of CAR immune cell therapy. METTL3 plays a role in T cell dysfunction via MDSC recruitment.^237^ METTL14 is also associated with T‐cell dysfunction.^236^ The expansion of CAR immune cells is essential. Targeting m6A modifications promotes immune cell proliferation. For example, YTHDF2 regulates NK cell function and proliferation.^266^ These results may aid future CAR‐NK cell therapy. In addition, it is worth exploring whether targeting the m6A modification of immune cells derived from patients can promote CAR expression, which may promote CAR immune cell therapy efficacy.

### 8.5 Direct‐targeted treatment of m6A regulators

m6A modifications play various roles in the occurrence and development of tumors, and therapies targeting m6A‐related molecules are also diverse.^379^, ^380^ Multiple studies have shown that m6A modifications affect the sensitivity of chemotherapy drugs as a direct target and thus affect the treatment of cancer.^381^, ^382^, ^383^, ^384^ Therefore, an increasing number of therapies that directly target m6A regulators have been reported. The two most widely regulated proteins are METTL3 and FTO, which coordinate during the dynamic reversible m6A modification process. At present, a small‐molecule inhibitor targeting METTL3 has entered clinical research, and the other four are in preclinical research, all of which are used to treat cancer or leukemia. The same is true for small‐molecule inhibitors that target FTO.

#### 8.5.1 METTL3‐targeted drugs

In 2017, METTL3 was shown to promote the development of AML.^385^ In 2021, STM2457, a small‐molecule inhibitor of METTL3 with in vivo activity, was first identified and further demonstrated to be effective against AML progression.^386^ STM2457 is a potent and highly specific inhibitor of the METTL3–METTL14 catalytic activity, without affecting other RNA methyltransferases. By binding to METTL3, STM2457 inhibits the translation of m6A‐positive genes, thereby impeding the progression of AML while sparing normal hematopoietic stem cells and other normal cells. In vivo assays have confirmed that STM2457 inhibits the proliferation and expansion of AML cells and significantly prolongs the lifespan of mice, without causing significant toxic side effects or affecting body weight. In April 2021, STM2457 entered preclinical research trials for the treatment of myeloid leukemia, conducted by The Wellcome Trust Sanger Institute, the University of Cambridge, and Storm Therapeutics Ltd. In addition to AML, STM2457 alone or in combination with other drugs has shown therapeutic effects in a variety of tumors, including HCC,^356^, ^387^ ICC,^388^ neuroblastoma,^389^, ^390^ colorectal cancer,^391^ renal cell carcinoma,^392^ and NSCLC.^393^, ^394^

With the continuous progress of research, a new generation of METTL3 inhibitors on STC‐15 was discovered, which is a derivative of STM2457, the first molecule specifically targeting an RNA methyltransferase enzyme to enter clinical development. In November 2022, STORM Therapeutics Ltd. began a phase I clinical trial for advanced solid tumors. In June 2024, the research team presented interim phase 1 clinical data for STC‐15 at the American Society of Clinical Oncology 2024, STC‐15 was well tolerated, and clinical activity was observed across the pharmacologically active dose range in patients with advanced cancer. They will also study STC‐15 in combination with checkpoint inhibitors.

Two other drugs targeting METTL3 in preclinical studies are EP‐102 and UZH2. EP‐102 inhibits AML cell proliferation and acts synergistically with venetoclax, a selective BCL‐2 inhibitor.^395^ Preclinical research trials on EP‐102 indications for solid tumors have been conducted by EPICS Therapeutics, Ltd. in December 2023. The other UZH2 showed target engagement in cells and reduced the m6A/A levels of polyadenylated RNA in AML and prostate cancer cell lines.^396^ It is undergoing preclinical studies for cancer by the University of Zürich.

#### 8.5.2 FTO‐targeted drugs

FTO promoted cancer cell growth, self‐renewal, metastasis, and immune escape. FB23‐2 is an analog of FTO that selectively inhibits meclofenamic acid (MA); however, its activity is significantly higher than that of MA and FB23.^397^ It promoted AML cell differentiation/apoptosis and inhibited the progression of primary cells in xenotransplanted mice.^398^ In April 2019, a preclinical study of FB23‐2 for the treatment of AML was conducted by Shanghai Institute of Materia Medica, Chinese Academy of Sciences. ZLD115 is a flexible alkaline side‐chain‐substituted benzoic acid FTO inhibitor derived from FB23. It showed a better drug similarity than FB23. ZLD115 exhibited significant antiproliferative activity in leukemic NB4 and MOLM13 cell lines and antileukemic activity in xenograft mice without substantial side effects.^399^ In July 2023, the Shanghai Institute of Materia Medica, Chinese Academy of Sciences, and Hangzhou Institute of Advanced Research, University of Chinese Academy of Sciences, began a preclinical trial of ZLD115 for the treatment of leukemia.

FTO‐04, a derivative of FB23 created through rational design, effectively hinders the formation of neurospheres by CSCs in GBM patients, while leaving nonmalignant neural stem cells unaffected.^400^ Through further rational structure‐based enhancements, FTO‐04 was refined to FTO‐43, an exceptionally selective oxetanyl‐class inhibitor of FTO. FTO‐43 elevates the m6A levels in GC cells to a degree comparable to that of FTO knockdown, impeding the in vitro growth of GC, GBM, and AML cells.^401^ In August 2022, FTO‐43 was used to treat tumors in preclinical studies conducted at the University of California in San Diego.

CS1 (bisantrene) and CS2 (brequinar) have been identified as specific inhibitors of FTO, which can inhibit the self‐renewal and immune evasion of cancer stem cells and exhibit potent antitumor effects in many types of cancer. They can significantly attenuate the self‐renewal and reprogramming of leukemia stem/initiating cells by inhibiting the expression of immune checkpoint genes, especially LILRB4.^402^ CS1 and CS2 showed higher efficacy than FB23‐2 at inhibiting AML cell viability. Among the FTO‐targeted drugs, bisanthrene was the first inhibitor to enter clinical trials. In this phase II study, the clinical safety and efficacy of bisantrene were evaluated in an initial cohort of patients with relapsed/refractory AML.^403^ Of the 10 patients enrolled in the study, four showed a clinical response to bisantrene with acceptable cardiac toxicity. Given the observed low toxicity, a follow‐up study is planned to combine bisantrene with a complementary antileukemia therapy. These findings suggest that bisantrene has a promising antileukemic activity and an acceptable safety profile. Small‐molecule drugs targeting m6A regulators are gradually being developed and optimized. In the future, more of these drugs are expected to enter clinical research, either alone or in combination with chemotherapeutic agents or checkpoint inhibitors, to treat cancer. Thus, m6A can be leveraged in cancer immunotherapy through indirect RNA therapy, ICIs, cytokine therapy, and direct targeting of m6A regulators.

## 9 CONCLUSION AND PROSPECTS

m6A, the most prevalent form of RNA modification, has garnered significant attention for its pivotal role in human diseases, particularly cancer. Writers, erasers, and readers collectively regulate the dynamic m6A processes. Importantly, the expression and activity of these regulatory proteins can be modulated by various chemical modifications such as lactylation, acetylation, ubiquitination, phosphorylation, SUMOylation, and O‐GlcNAcylation. Furthermore, m6A modification closely intertwines with chromatin accessibility, thereby influencing transcriptional activity. Drugs targeting PTMs of m6A regulators offer promising avenues for novel cancer treatment strategies.

PCD exhibits dual roles in tumorigenesis, influenced in part by the substances released into the intracellular environment during the process. PCD plays a crucial role in modulating tumor immunity. Targeting m6A modifications directly affects cancer cell death by influencing PCD‐related pathways. Conversely, m6A modifications impact immune cell function through their effects on PCD. Moreover, m6A is involved in various processes within tumor‐associated immune cells, including proliferation, differentiation, polarization, recruitment, and activation. Therefore, further investigation into the role of m6A in cancer cell‐immune cell interactions holds promise for developing novel strategies to enhance cancer immunotherapy.

m6A modifications play a pivotal role in microorganism‐associated cancers. On one hand, m6A influences the RNA expression and life cycle of specific pathogens, affecting their infection and oncogenic potential. Conversely, m6A modifications can impact the efficacy of anti‐infection and anticancer treatments. Additionally, microorganisms utilize a “hit‐and‐run” mechanism to induce lasting epigenetic changes in host cells through modulation of m6A modifications. Together, the interplay between m6A modifications and microorganisms in microorganism‐associated cancers represents a promising avenue for future prevention and treatment strategies.

Our focus centers on the role of m6A modifications in immunotherapy, with potential future benefits in enhancing the expression of TAAs or TSAs to improve mRNA vaccine efficacy. The process may involve m6A‐related LLPS. Notably, targeting m6A modifications in virus‐related cancers holds therapeutic promise. Engineered EVs can specifically modulate m6A modifications on key target mRNAs, influencing cancer cell–immune cell interactions. m6A modifications may enhance ICB by regulating the expression of immunosuppressive molecules and immune cell infiltration, thereby alleviating immunosuppression and drug resistance.Moreover, constructing and delivering m6A‐modified cytokine‐related mRNAs into the TME could persistently alter cytokine expression, potentially aiding cancer immunotherapy. Despite successful ACT outcomes in specific leukemias and lymphomas, challenges such as primary resistance, relapse, and adverse events persist, particularly in solid tumors. The development of drugs targeting m6A regulators shows promise, with several in clinical trials and numerous others in preclinical stages, highlighting significant potential for future clinical applications. In the future, there will be more drugs, alone or in combination, for the treatment of advanced cancers.

## AUTHOR CONTRIBUTIONS

Q. Y., G. N. N., Z. S. W., and G. L. M. wrote the manuscript and generated figures and tables. H. B., W. C., and G. C. L. revised the manuscript. S. Q. Y. and L. Z. B. projected and edited the manuscript. Y. S. M. and X. Y. F. reviewed the manuscript. All authors read and approved the final manuscript.

## CONFLICT OF INTEREST STATEMENT

The authors declare that they have no conflict of interest.

## ETHICS STATEMENT

Not applicable.

## Data Availability

Not applicable.

## References

[1] Siegel RL , Miller KD , Wagle NS , Jemal A . Cancer statistics, 2023. CA Cancer J Clin. 2023;73(1):17‐48. 36633525 10.3322/caac.21763

[2] Peixoto P , Cartron PF , Serandour AA , Hervouet E . From 1957 to nowadays: a brief history of epigenetics. Int J Mol Sci. 2020;21(20):7571. 33066397 10.3390/ijms21207571 PMC7588895

[3] Fitz‐James MH , Cavalli G . Molecular mechanisms of transgenerational epigenetic inheritance. Nat Rev Genet. 2022;23(6):325‐341. 34983971 10.1038/s41576-021-00438-5 PMC7619059

[4] R Desrosiers KF , Rottman F . Identification of methylated nucleosides in messenger RNA from Novikoff hepatoma cells. Proc Natl Acad Sci USA. 1974;71(10):3971‐3975. 4372599 10.1073/pnas.71.10.3971 PMC434308

[5] Gan L , Zhao Y , Fu Y , Chen Q . The potential role of m6A modifications on immune cells and immunotherapy. Biomed Pharmacother. 2023;160:114343. 36758318 10.1016/j.biopha.2023.114343

[6] Liu J , Dou X , Chen C , et al. N 6‐methyladenosine of chromosome‐associated regulatory RNA regulates chromatin state and transcription. Science (New York, NY). 2020;367(6477):580‐586. 10.1126/science.aay6018 PMC7213019 31949099

[7] Chen X‐Y , Zhang J , Zhu J‐S . The role of m6A RNA methylation in human cancer. Mol Cancer. 2019;18(1):103. 31142332 10.1186/s12943-019-1033-z PMC6540575

[8] Wu S , Zhang S , Wu X , Zhou X . m6A RNA methylation in cardiovascular diseases. Mol Ther. 2020;28(10):2111‐2119. 32910911 10.1016/j.ymthe.2020.08.010 PMC7544996

[9] Wu S , Li XF , Wu YY , Yin SQ , Huang C , Li J . N(6) ‐Methyladenosine and rheumatoid arthritis: a comprehensive review. Front Immunol. 2021;12:731842. 34630412 10.3389/fimmu.2021.731842 PMC8498590

[10] Lv J , Xing L , Zhong X , Li K , Liu M , Du K . Role of N6‐methyladenosine modification in central nervous system diseases and related therapeutic agents. Biomed Pharmacother. 2023;162:114583. 36989722 10.1016/j.biopha.2023.114583

[11] Chen J , Fang Y , Xu Y , Sun H . Role of m6A modification in female infertility and reproductive system diseases. Int J Biol Sci. 2022;18(9):3592‐3604. 35813486 10.7150/ijbs.69771 PMC9254474

[12] Zhang Y , Chen W , Zheng X , et al. Regulatory role and mechanism of m(6)A RNA modification in human metabolic diseases. Mol Ther Oncolytics. 2021;22:52‐63. 34485686 10.1016/j.omto.2021.05.003 PMC8399361

[13] Lu Y , Yuan X , Wang M , et al. Gut microbiota influence immunotherapy responses: mechanisms and therapeutic strategies. J Hematol Oncol. 2022;15(1):47. 35488243 10.1186/s13045-022-01273-9 PMC9052532

[14] Chi C , Du Y , Ye J , et al. Intraoperative imaging‐guided cancer surgery: from current fluorescence molecular imaging methods to future multi‐modality imaging technology. Theranostics. 2014;4(11):1072‐1084. 25250092 10.7150/thno.9899 PMC4165775

[15] Wei G , Wang Y , Yang G , Wang Y , Ju R . Recent progress in nanomedicine for enhanced cancer chemotherapy. Theranostics. 2021;11(13):6370‐6392. 33995663 10.7150/thno.57828 PMC8120226

[16] Schaue D , McBride WH . Opportunities and challenges of radiotherapy for treating cancer. Nat Rev Clin Oncol. 2015;12(9):527‐540. 26122185 10.1038/nrclinonc.2015.120 PMC8396062

[17] Robert HE , Markus S . Cell‐free DNA as a biomarker in cancer. Extracell Vesicles Circ Nucl Acids. 2022;3(3):195‐215. 10.20517/evcna.2022.20 PMC11648514 39697490

[18] Park W , Chawla A , O'Reilly EM . Pancreatic cancer: a review. JAMA. 2021;326(9):851‐862. 34547082 10.1001/jama.2021.13027 PMC9363152

[19] Kennedy LB , Salama AKS . A review of cancer immunotherapy toxicity. CA Cancer J Clin. 2020;70(2):86‐104. 31944278 10.3322/caac.21596

[20] Zhang Y , Zhang Z . The history and advances in cancer immunotherapy: understanding the characteristics of tumor‐infiltrating immune cells and their therapeutic implications. Cell Mol Immunol. 2020;17(8):807‐821. 32612154 10.1038/s41423-020-0488-6 PMC7395159

[21] Swamydas M , Murphy EV , Ignatz‐Hoover JJ , Malek E , Driscoll JJ . Deciphering mechanisms of immune escape to inform immunotherapeutic strategies in multiple myeloma. J Hematol Oncol. 2022;15(1):17. 35172851 10.1186/s13045-022-01234-2 PMC8848665

[22] O'Donnell JS , Teng MWL , Smyth MJ . Cancer immunoediting and resistance to T cell‐based immunotherapy. Nat Rev Clin Oncol. 2019;16(3):151‐167. 30523282 10.1038/s41571-018-0142-8

[23] Oerum S , Meynier V , Catala M , Tisne C . A comprehensive review of m6A/m6Am RNA methyltransferase structures. Nucleic Acids Res. 2021;49(13):7239‐7255. 34023900 10.1093/nar/gkab378 PMC8287941

[24] Liu J , Yue Y , Han D , et al. A METTL3‐METTL14 complex mediates mammalian nuclear RNA N6‐adenosine methylation. Nat Chem Biol. 2014;10(2):93‐95. 24316715 10.1038/nchembio.1432 PMC3911877

[25] Zhou KI , Pan T . Structures of the m(6)A methyltransferase complex: two subunits with distinct but coordinated roles. Mol Cell. 2016;63(2):183‐185. 27447983 10.1016/j.molcel.2016.07.005 PMC5109923

[26] Ping XL , Sun BF , Wang L , et al. Mammalian WTAP is a regulatory subunit of the RNA N6‐methyladenosine methyltransferase. Cell Res. 2014;24(2):177‐189. 24407421 10.1038/cr.2014.3 PMC3915904

[27] van Tran N , Ernst FGM , Hawley BR , et al. The human 18S rRNA m6A methyltransferase METTL5 is stabilized by TRMT112. Nucleic Acids Res. 2019;47(15):7719‐7733. 31328227 10.1093/nar/gkz619 PMC6735865

[28] Wang Z , He J , Bach DH , et al. Induction of m(6)A methylation in adipocyte exosomal LncRNAs mediates myeloma drug resistance. J Exp Clin Cancer Res. 2022;41(1):4. 34980213 10.1186/s13046-021-02209-w PMC8722039

[29] Liu Z , Chen Y , Shen T . Evidence based on an integrative analysis of multi‐omics data on METTL7A as a molecular marker in pan‐cancer. Biomolecules. 2023;13(2):195. 36830565 10.3390/biom13020195 PMC9952925

[30] Song H , Liu D , Wang L , et al. Methyltransferase like 7B is a potential therapeutic target for reversing EGFR‐TKIs resistance in lung adenocarcinoma. Mol Cancer. 2022;21(1):43. 35144642 10.1186/s12943-022-01519-7 PMC8830004

[31] Luo C , Wang S , Shan W , et al. A whole exon screening‐based score model predicts prognosis and immune checkpoint inhibitor therapy effects in low‐grade glioma. Front Immunol. 2022;13:909189. 35769464 10.3389/fimmu.2022.909189 PMC9234137

[32] Ruszkowska A . METTL16, methyltransferase‐like protein 16: current insights into structure and function. Int J Mol Sci. 2021;22(4):2176. 33671635 10.3390/ijms22042176 PMC7927073

[33] Satterwhite ER , Mansfield KD . RNA methyltransferase METTL16: targets and function. Wiley Interdiscip Rev RNA. 2022;13(2):e1681. 34227247 10.1002/wrna.1681 PMC9286414

[34] Zhang X , Li MJ , Xia L , Zhang H . The biological function of m6A methyltransferase KIAA1429 and its role in human disease. PeerJ. 2022;10:e14334. 36389416 10.7717/peerj.14334 PMC9657180

[35] Zhang C , Sun Q , Zhang X , et al. Gene amplification‐driven RNA methyltransferase KIAA1429 promotes tumorigenesis by regulating BTG2 via m6A‐YTHDF2‐dependent in lung adenocarcinoma. Cancer Commun (Lond). 2022;42(7):609‐626. 35730068 10.1002/cac2.12325 PMC9257983

[36] Wang X , Tian L , Li Y , et al. RBM15 facilitates laryngeal squamous cell carcinoma progression by regulating TMBIM6 stability through IGF2BP3 dependent. J Exp Clin Cancer Res. 2021;40(1):80. 33637103 10.1186/s13046-021-01871-4 PMC7912894

[37] Ma H , Wang X , Cai J , et al. N(6‐)Methyladenosine methyltransferase ZCCHC4 mediates ribosomal RNA methylation. Nat Chem Biol. 2019;15(1):88‐94. 30531910 10.1038/s41589-018-0184-3 PMC6463480

[38] Wen J , Lv R , Ma H , et al. Zc3h13 Regulates Nuclear RNA m(6)A Methylation and Mouse Embryonic Stem Cell Self‐Renewal. Mol Cell. 2018;69(6):1028‐1038. 29547716 10.1016/j.molcel.2018.02.015 PMC5858226

[39] Zhao W , Li J , Ma Q , et al. N6‐methyladenosine modification participates in neoplastic immunoregulation and tumorigenesis. J Cell Physiol. 2022;237(7):2729‐2739. 35342948 10.1002/jcp.30730

[40] Yue B , Song C , Yang L , et al. METTL3‐mediated N6‐methyladenosine modification is critical for epithelial‐mesenchymal transition and metastasis of gastric cancer. Mol Cancer. 2019;18(1):142. 31607270 10.1186/s12943-019-1065-4 PMC6790244

[41] Li J , Gregory RI . Mining for METTL3 inhibitors to suppress cancer. Nat Struct Mol Biol. 2021;28(6):460‐462. 34040230 10.1038/s41594-021-00606-5 PMC8197751

[42] Wang N , Huo X , Zhang B , et al. METTL3‐mediated ADAMTS9 suppression facilitates angiogenesis and carcinogenesis in gastric cancer. Front Oncol. 2022;12:861807. 35574388 10.3389/fonc.2022.861807 PMC9097454

[43] Zhou D , Tang W , Xu Y , et al. METTL3/YTHDF2 m6A axis accelerates colorectal carcinogenesis through epigenetically suppressing YPEL5. Mol Oncol. 2021;15(8):2172‐2184. 33411363 10.1002/1878-0261.12898 PMC8333777

[44] Jia G , Fu Y , Zhao X , et al. N6‐methyladenosine in nuclear RNA is a major substrate of the obesity‐associated FTO. Nat Chem Biol. 2011;7(12):885‐887. 22002720 10.1038/nchembio.687 PMC3218240

[45] Lan N , Lu Y , Zhang Y , et al. FTO—a common genetic basis for obesity and cancer. Front Genet. 2020;11:559138. 33304380 10.3389/fgene.2020.559138 PMC7701174

[46] Azzam SK , Alsafar H , Sajini AA . FTO m6A demethylase in obesity and cancer: implications and underlying molecular mechanisms. Int J Mol Sci. 2022;23(7):3800. 35409166 10.3390/ijms23073800 PMC8998816

[47] Qu J , Yan H , Hou Y , et al. RNA demethylase ALKBH5 in cancer: from mechanisms to therapeutic potential. J Hematol Oncol. 2022;15(1):8. 35063010 10.1186/s13045-022-01224-4 PMC8780705

[48] Trewick SC , Henshaw TF , Hausinger RP , Lindahl T , Sedgwick B . Oxidative demethylation by Escherichia coli AlkB directly reverts DNA base damage. Nature. 2002;419(6903):174‐178. 12226667 10.1038/nature00908

[49] Wang J , Wang J , Gu Q , et al. The biological function of m6A demethylase ALKBH5 and its role in human disease. Cancer Cell Int. 2020;20:347. 32742194 10.1186/s12935-020-01450-1 PMC7388453

[50] Wu Y , Wang Z , Han L , et al. PRMT5 regulates RNA m6A demethylation for doxorubicin sensitivity in breast cancer. Mol Ther. 2022;30(7):2603‐2617. 35278676 10.1016/j.ymthe.2022.03.003 PMC9263239

[51] Tsuchiya K , Yoshimura K , Inoue Y , et al. YTHDF1 and YTHDF2 are associated with better patient survival and an inflamed tumor‐immune microenvironment in non‐small‐cell lung cancer. Oncoimmunology. 2021;10(1):1962656. 34408926 10.1080/2162402X.2021.1962656 PMC8366544

[52] Liu T , Wei Q , Jin J , et al. The m6A reader YTHDF1 promotes ovarian cancer progression via augmenting EIF3C translation. Nucleic Acids Res. 2020;48(7):3816‐3831. 31996915 10.1093/nar/gkaa048 PMC7144925

[53] Chang G , Shi L , Ye Y , et al. YTHDF3 induces the translation of m(6)A‐enriched gene transcripts to promote breast cancer brain metastasis. Cancer Cell. 2020;38(6):857‐871. 33125861 10.1016/j.ccell.2020.10.004 PMC7738369

[54] Chen D , Cheung H , Lau HC , Yu J , Wong CC . N(6)‐Methyladenosine RNA‐binding protein YTHDF1 in gastrointestinal cancers: function, molecular mechanism and clinical implication. Cancers (Basel). 2022;14(14):3489. 35884552 10.3390/cancers14143489 PMC9320224

[55] Xiao W , Adhikari S , Dahal U , et al. Nuclear m(6)A reader YTHDC1 regulates mRNA splicing. Mol Cell. 2016;61(4):507‐519. 26876937 10.1016/j.molcel.2016.01.012

[56] Hsu PJ , Zhu Y , Ma H , et al. Ythdc2 is an N(6)‐methyladenosine binding protein that regulates mammalian spermatogenesis. Cell Res. 2017;27(9):1115‐1127. 28809393 10.1038/cr.2017.99 PMC5587856

[57] Wang J , Chen L , Qiang P . The role of IGF2BP2, an m6A reader gene, in human metabolic diseases and cancers. Cancer Cell Int. 2021;21(1):99. 33568150 10.1186/s12935-021-01799-x PMC7876817

[58] Zhang N , Shen Y , Li H , et al. The m6A reader IGF2BP3 promotes acute myeloid leukemia progression by enhancing RCC2 stability. Exp Mol Med. 2022;54(2):194‐205. 35217832 10.1038/s12276-022-00735-x PMC8894383

[59] Huang H , Weng H , Sun W , et al. Recognition of RNA N(6)‐methyladenosine by IGF2BP proteins enhances mRNA stability and translation. Nat Cell Biol. 2018;20(3):285‐295. 29476152 10.1038/s41556-018-0045-z PMC5826585

[60] Jiang F , Tang X , Tang C , et al. HNRNPA2B1 promotes multiple myeloma progression by increasing AKT3 expression via m6A‐dependent stabilization of ILF3 mRNA. J Hematol Oncol. 2021;14(1):54. 33794982 10.1186/s13045-021-01066-6 PMC8017865

[61] Liu H , Li D , Sun L , et al. Interaction of lncRNA MIR100HG with hnRNPA2B1 facilitates m(6)A‐dependent stabilization of TCF7L2 mRNA and colorectal cancer progression. Mol Cancer. 2022;21(1):74. 35279145 10.1186/s12943-022-01555-3 PMC8917698

[62] Wu R , Li A , Sun B , et al. A novel m(6)A reader Prrc2a controls oligodendroglial specification and myelination. Cell Res. 2019;29(1):23‐41. 30514900 10.1038/s41422-018-0113-8 PMC6318280

[63] Cai Z , Xu H , Bai G , et al. ELAVL1 promotes prostate cancer progression by interacting with other m6A regulators. Front Oncol. 2022;12:939784. 35978821 10.3389/fonc.2022.939784 PMC9376624

[64] Zhang F , Kang Y , Wang M , et al. Fragile X mental retardation protein modulates the stability of its m6A‐marked messenger RNA targets. Hum Mol Genet. 2018;27(22):3936‐3950. 30107516 10.1093/hmg/ddy292 PMC6216232

[65] Chen H , Yu Y , Yang M , et al. YTHDF1 promotes breast cancer progression by facilitating FOXM1 translation in an m6A‐dependent manner. Cell Biosci. 2022;12(1):19. 35197112 10.1186/s13578-022-00759-w PMC8867832

[66] Zaccara S , Jaffrey SR . A unified model for the function of YTHDF proteins in regulating m(6)A‐modified mRNA. Cell. 2020;181(7):1582‐1595. 32492408 10.1016/j.cell.2020.05.012 PMC7508256

[67] Fu Y , Zhuang X . m(6)A‐binding YTHDF proteins promote stress granule formation. Nat Chem Biol. 2020;16(9):955‐963. 32451507 10.1038/s41589-020-0524-y PMC7442727

[68] Zou Z , Sepich‐Poore C , Zhou X , Wei J , He C . The mechanism underlying redundant functions of the YTHDF proteins. Genome Biol. 2023;24(1):17. 36694229 10.1186/s13059-023-02862-8 PMC9872407

[69] Riggs CL , Kedersha N , Ivanov P , Anderson P . Mammalian stress granules and P bodies at a glance. J Cell Sci. 2020;133(16):jcs242487. 32873715 10.1242/jcs.242487 PMC10679417

[70] Roundtree IA , Luo GZ , Zhang Z , et al. YTHDC1 mediates nuclear export of N(6)‐methyladenosine methylated mRNAs. Elife. 2017:6. 10.7554/eLife.31311 PMC5648532 28984244

[71] Widagdo J , Anggono V , Wong JJ . The multifaceted effects of YTHDC1‐mediated nuclear m(6)A recognition. Trends Genet. 2022;38(4):325‐332. 34920906 10.1016/j.tig.2021.11.005

[72] Alarcón CR , Goodarzi H , Lee H , Liu X , Tavazoie S , Tavazoie SF . HNRNPA2B1 is a mediator of m(6)A‐dependent nuclear RNA processing events. Cell. 2015;162(6):1299‐1308. 26321680 10.1016/j.cell.2015.08.011 PMC4673968

[73] Wang L , Wen M , Cao X . Nuclear hnRNPA2B1 initiates and amplifies the innate immune response to DNA viruses. Science. 2019;365(6454):eaav0758. 31320558 10.1126/science.aav0758

[74] Liu N , Dai Q , Zheng G , He C , Parisien M , Pan T . N(6)‐methyladenosine‐dependent RNA structural switches regulate RNA‐protein interactions. Nature. 2015;518(7540):560‐564. 25719671 10.1038/nature14234 PMC4355918

[75] Zhou KI , Shi H , Lyu R , et al. Regulation of co‐transcriptional Pre‐mRNA splicing by m(6)A through the low‐complexity protein hnRNPG. Mol Cell. 2019;76(1):70‐81. 31445886 10.1016/j.molcel.2019.07.005 PMC6778029

[76] Chen Y , Peng C , Chen J , et al. WTAP facilitates progression of hepatocellular carcinoma via m6A‐HuR‐dependent epigenetic silencing of ETS1. Mol Cancer. 2019;18(1):127. 31438961 10.1186/s12943-019-1053-8 PMC6704583

[77] Hu Y , Gao Q , Ma S , et al. FMR1 promotes the progression of colorectal cancer cell by stabilizing EGFR mRNA in an m(6)A‐dependent manner. Cell Death Dis. 2022;13(11):941. 36347844 10.1038/s41419-022-05391-7 PMC9643526

[78] Wang J , Wang Z , Inuzuka H , Wei W , Liu J . PRMT1 methylates METTL14 to modulate its oncogenic function. Neoplasia. 2023;42:100912. 37269817 10.1016/j.neo.2023.100912 PMC10248872

[79] Wang Y , Wang C , Guan X , et al. PRMT3‐mediated arginine methylation of METTL14 promotes malignant progression and treatment resistance in endometrial carcinoma. Adv Sci (Weinh). 2023;10(36):e2303812. 37973560 10.1002/advs.202303812 PMC10754120

[80] Jia Y , Yu X , Liu R , et al. PRMT1 methylation of WTAP promotes multiple myeloma tumorigenesis by activating oxidative phosphorylation via m6A modification of NDUFS6. Cell Death Dis. 2023;14(8):512. 37558663 10.1038/s41419-023-06036-z PMC10412649

[81] Han X , Ren C , Jiang A , et al. Arginine methylation of ALKBH5 by PRMT6 promotes breast tumorigenesis via LDHA‐mediated glycolysis. Front Med. 2024;18(2):344‐356. 38466502 10.1007/s11684-023-1028-4

[82] Gil J , Ramírez‐Torres A , Encarnación‐Guevara S . Lysine acetylation and cancer: a proteomics perspective. J Proteomics. 2017;150:297‐309. 27746255 10.1016/j.jprot.2016.10.003

[83] Li Y , He X , Lu X , et al. METTL3 acetylation impedes cancer metastasis via fine‐tuning its nuclear and cytosolic functions. Nat Commun. 2022;13(1):6350. 36289222 10.1038/s41467-022-34209-5 PMC9605963

[84] Zhang G , Huang R , Zhao H , et al. ACAT1‐mediated METTL3 acetylation inhibits cell migration and invasion in triple negative breast cancer. Genes Immun. 2023;24(2):99‐107. 36890220 10.1038/s41435-023-00202-1

[85] Yang Y , Qian Cai Q , Sheng Fu L , Wei Dong Y , Fan F , Zhong Wu X . Reduced N6‐methyladenosine mediated by METTL3 acetylation promotes MTF1 expression and hepatocellular carcinoma cell growth. Chem Biodivers. 2022;19(11):e202200333. 36149370 10.1002/cbdv.202200333

[86] Wang X , Ding Y , Li R , et al. N(6)‐methyladenosine of Spi2a attenuates inflammation and sepsis‐associated myocardial dysfunction in mice. Nat Commun. 2023;14(1):1185. 36864027 10.1038/s41467-023-36865-7 PMC9979126

[87] Zhang X‐L , Chen X‐H , Xu B , et al. K235 acetylation couples with PSPC1 to regulate the m6A demethylation activity of ALKBH5 and tumorigenesis. Nat Commun. 2023;14(1):3815. 37369679 10.1038/s41467-023-39414-4 PMC10300122

[88] Latifkar A , Wang F , Mullmann JJ , et al. IGF2BP2 promotes cancer progression by degrading the RNA transcript encoding a v‐ATPase subunit. Proc Natl Acad Sci USA. 2022;119(45):e2200477119. 36322753 10.1073/pnas.2200477119 PMC9659396

[89] Icard P , Shulman S , Farhat D , Steyaert JM , Alifano M , Lincet H . How the Warburg effect supports aggressiveness and drug resistance of cancer cells? Drug Resist Updat. 2018;38:1‐11. 29857814 10.1016/j.drup.2018.03.001

[90] Zhou Y , Lin F , Wan T , et al. ZEB1 enhances Warburg effect to facilitate tumorigenesis and metastasis of HCC by transcriptionally activating PFKM. Theranostics. 2021;11(12):5926‐5938. 33897890 10.7150/thno.56490 PMC8058737

[91] Sun Y , Chen Y , Peng T . A bioorthogonal chemical reporter for the detection and identification of protein lactylation. Chem Sci. 2022;13(20):6019‐6027. 35685793 10.1039/d2sc00918h PMC9132054

[92] Zhang D , Tang Z , Huang H , et al. Metabolic regulation of gene expression by histone lactylation. Nature. 2019;574(7779):575‐580. 31645732 10.1038/s41586-019-1678-1 PMC6818755

[93] Yu J , Chai P , Xie M , et al. Histone lactylation drives oncogenesis by facilitating m6A reader protein YTHDF2 expression in ocular melanoma. Genome Biol. 2021;22(1):85. 33726814 10.1186/s13059-021-02308-z PMC7962360

[94] Wang P , Xie D , Xiao T , et al. H3K18 lactylation promotes the progression of arsenite‐related idiopathic pulmonary fibrosis via YTHDF1/m6A/NREP. J Hazard Mater. 2023;461:132582. 37742376 10.1016/j.jhazmat.2023.132582

[95] Xiong J , He J , Zhu J , et al. Lactylation‐driven METTL3‐mediated RNA m(6)A modification promotes immunosuppression of tumor‐infiltrating myeloid cells. Mol Cell. 2022;82(9):1660‐1677. 35320754 10.1016/j.molcel.2022.02.033

[96] Sun L , Zhang Y , Yang B , et al. Lactylation of METTL16 promotes cuproptosis via m(6)A‐modification on FDX1 mRNA in gastric cancer. Nat Commun. 2023;14(1):6523. 37863889 10.1038/s41467-023-42025-8 PMC10589265

[97] Leestemaker Y , Ovaa H . Tools to investigate the ubiquitin proteasome system. Drug Discov Today Technol. 2017;26:25‐31. 29249239 10.1016/j.ddtec.2017.11.006

[98] Swatek KN , Usher JL , Kueck AF , et al. Insights into ubiquitin chain architecture using Ub‐clipping. Nature. 2019;572(7770):533‐537. 31413367 10.1038/s41586-019-1482-y PMC6823057

[99] Martinez‐Ferriz A , Ferrando A , Fathinajafabadi A , Farras R . Ubiquitin‐mediated mechanisms of translational control. Semin Cell Dev Biol. 2022;132:146‐154. 34952788 10.1016/j.semcdb.2021.12.009

[100] Wei J , Harada BT , Lu D , et al. HRD1‐mediated METTL14 degradation regulates m(6)A mRNA modification to suppress ER proteotoxic liver disease. Mol Cell. 2021;81(24):5052‐5065. 34847358 10.1016/j.molcel.2021.10.028 PMC8751812

[101] Ruan DY , Li T , Wang YN , et al. FTO downregulation mediated by hypoxia facilitates colorectal cancer metastasis. Oncogene. 2021;40(33):5168‐5181. 34218271 10.1038/s41388-021-01916-0 PMC8376648

[102] Liang W , Liu H , Zeng Z , et al. KRT17 promotes T‐lymphocyte infiltration through the YTHDF2‐CXCL10 axis in colorectal cancer. Cancer Immunol Res. 2023;11(7):875‐894. 37129929 10.1158/2326-6066.CIR-22-0814 PMC10320689

[103] Li B , Zhu L , Lu C , et al. circNDUFB2 inhibits non‐small cell lung cancer progression via destabilizing IGF2BPs and activating anti‐tumor immunity. Nat Commun. 2021;12(1):295. 33436560 10.1038/s41467-020-20527-z PMC7804955

[104] Lin XT , Yu HQ , Fang L , et al. Elevated FBXO45 promotes liver tumorigenesis through enhancing IGF2BP1 ubiquitination and subsequent PLK1 upregulation. Elife. 2021:10. 10.7554/eLife.70715 PMC8641947 34779401

[105] Yao B , Zhang Q , Yang Z , et al. CircEZH2/miR‐133b/IGF2BP2 aggravates colorectal cancer progression via enhancing the stability of m(6)A‐modified CREB1 mRNA. Mol Cancer. 2022;21(1):140. 35773744 10.1186/s12943-022-01608-7 PMC9245290

[106] Wang Y , Lu JH , Wu QN , et al. LncRNA LINRIS stabilizes IGF2BP2 and promotes the aerobic glycolysis in colorectal cancer. Mol Cancer. 2019;18(1):174. 31791342 10.1186/s12943-019-1105-0 PMC6886219

[107] Shi J , Zhang Q , Yin X , et al. Stabilization of IGF2BP1 by USP10 promotes breast cancer metastasis via CPT1A in an m6A‐dependent manner. Int J Biol Sci. 2023;19(2):449‐464. 36632454 10.7150/ijbs.76798 PMC9830507

[108] Han ZJ , Feng YH , Gu BH , Li YM , Chen H . The post‐translational modification, SUMOylation, and cancer (Review). Int J Oncol. 2018;52(4):1081‐1094. 29484374 10.3892/ijo.2018.4280 PMC5843405

[109] Yu F , Wei J , Cui X , et al. Post‐translational modification of RNA m6A demethylase ALKBH5 regulates ROS‐induced DNA damage response. Nucleic Acids Res. 2021;49(10):5779‐5797. 34048572 10.1093/nar/gkab415 PMC8191756

[110] Sugiokto FG , Saiada F , Zhang K , Li R . SUMOylation of the m6A reader YTHDF2 by PIAS1 promotes viral RNA decay to restrict EBV replication. bioRxiv. 2023. 10.1128/mbio.03168-23 PMC10865817 38236021

[111] Hou G , Zhao X , Li L , et al. SUMOylation of YTHDF2 promotes mRNA degradation and cancer progression by increasing its binding affinity with m6A‐modified mRNAs. Nucleic Acids Res. 2021;49(5):2859‐2877. 33577677 10.1093/nar/gkab065 PMC7969013

[112] Liu X , Liu J , Xiao W , et al. SIRT1 regulates N(6) ‐methyladenosine RNA modification in hepatocarcinogenesis by inducing RANBP2‐dependent FTO SUMOylation. Hepatology. 2020;72(6):2029‐2050. 32154934 10.1002/hep.31222

[113] Du Y , Hou G , Zhang H , et al. SUMOylation of the m6A‐RNA methyltransferase METTL3 modulates its function. Nucleic Acids Res. 2018;46(10):5195‐5208. 29506078 10.1093/nar/gky156 PMC6007514

[114] Singh V , Ram M , Kumar R , Prasad R , Roy BK , Singh KK . Phosphorylation: implications in cancer. Protein J. 2017;36(1):1‐6. 28108801 10.1007/s10930-017-9696-z

[115] Hunter T . A journey from phosphotyrosine to phosphohistidine and beyond. Mol Cell. 2022;82(12):2190‐2200. 35654043 10.1016/j.molcel.2022.05.007 PMC9219344

[116] Bilbrough T , Piemontese E , Seitz O . Dissecting the role of protein phosphorylation: a chemical biology toolbox. Chem Soc Rev. 2022;51(13):5691‐5730. 35726784 10.1039/d1cs00991e

[117] Sun HL , Zhu AC , Gao Y , et al. Stabilization of ERK‐Phosphorylated METTL3 by USP5 increases m(6)A methylation. Mol Cell. 2020;80(4):633‐647. 33217317 10.1016/j.molcel.2020.10.026 PMC7720844

[118] Perez‐Pepe M , Desotell AW , Li H , et al. 7SK methylation by METTL3 promotes transcriptional activity. Sci Adv. 2023;9(19):eade7500. 37163588 10.1126/sciadv.ade7500 PMC10171809

[119] Ou B , Liu Y , Yang X , Xu X , Yan Y , Zhang J . C5aR1‐positive neutrophils promote breast cancer glycolysis through WTAP‐dependent m6A methylation of ENO1. Cell Death Dis. 2021;12(8):737. 34312368 10.1038/s41419-021-04028-5 PMC8313695

[120] Chen J , Wei X , Wang X , et al. TBK1‐METTL3 axis facilitates antiviral immunity. Cell Rep. 2022;38(7):110373. 35172162 10.1016/j.celrep.2022.110373

[121] Fang R , Chen X , Zhang S , et al. EGFR/SRC/ERK‐stabilized YTHDF2 promotes cholesterol dysregulation and invasive growth of glioblastoma. Nat Commun. 2021;12(1):177. 33420027 10.1038/s41467-020-20379-7 PMC7794382

[122] Chang YH , Weng CL , Lin KI . O‐GlcNAcylation and its role in the immune system. J Biomed Sci. 2020;27(1):57. 32349769 10.1186/s12929-020-00648-9 PMC7189445

[123] Yang Y , Yan Y , Yin J , et al. O‐GlcNAcylation of YTHDF2 promotes HBV‐related hepatocellular carcinoma progression in an N(6)‐methyladenosine‐dependent manner. Signal Transduct Target Ther. 2023;8(1):63. 36765030 10.1038/s41392-023-01316-8 PMC9918532

[124] Li J , Ahmad M , Sang L , et al. O‐GlcNAcylation promotes the cytosolic localization of the m(6)A reader YTHDF1 and colorectal cancer tumorigenesis. J Biol Chem. 2023;299(6):104738. 37086786 10.1016/j.jbc.2023.104738 PMC10208891

[125] Deng S , Zhang J , Su J , et al. RNA m6A regulates transcription via DNA demethylation and chromatin accessibility. Nat Genet. 2022;54(9):1427‐1437. 36071173 10.1038/s41588-022-01173-1

[126] Li R , Zhao H , Huang X , et al. Super‐enhancer RNA m6A promotes local chromatin accessibility and oncogene transcription in pancreatic ductal adenocarcinoma. Nat Genet. 2023;55(12):2224‐2234. 37957340 10.1038/s41588-023-01568-8

[127] Yao L , Li T , Teng Y , et al. ALKHB5‐demethylated lncRNA SNHG15 promotes myeloma tumorigenicity by increasing chromatin accessibility and recruiting H3K36me3 modifier SETD2. Am J Physiol Cell Physiol. 2024;326(3):C684‐C697. 38145297 10.1152/ajpcell.00348.2023 PMC11193452

[128] Sun X , Bai C , Li H , et al. PARP1 modulates METTL3 promoter chromatin accessibility and associated LPAR5 RNA m6A methylation to control cancer cell radiosensitivity. Mol Ther. 2023;31(9):2633‐2650. 37482682 10.1016/j.ymthe.2023.07.018 PMC10492194

[129] Wang J , Li Y , Wang P , et al. Leukemogenic chromatin alterations promote AML leukemia stem cells via a KDM4C‐ALKBH5‐AXL signaling axis. Cell Stem Cell. 2020;27(1):81‐97. 32402251 10.1016/j.stem.2020.04.001

[130] Li F , Chen S , Yu J , et al. Interplay of m6 A and histone modifications contributes to temozolomide resistance in glioblastoma. Clin Transl Med. 2021;11(9):e553. 34586728 10.1002/ctm2.553 PMC8441140

[131] Tang B , Yan R , Zhu J , et al. Integrative analysis of the molecular mechanisms, immunological features and immunotherapy response of ferroptosis regulators across 33 cancer types. Int J Biol Sci. 2022;18(1):180‐198. 34975326 10.7150/ijbs.64654 PMC8692154

[132] Zhang Q , Tan Y , Zhang J , et al. Pyroptosis‐related signature predicts prognosis and immunotherapy efficacy in muscle‐invasive bladder cancer. Front Immunol. 2022;13:782982. 35479097 10.3389/fimmu.2022.782982 PMC9035667

[133] Hu B , Gao J , Shi J , et al. Necroptosis throws novel insights on patient classification and treatment strategies for hepatocellular carcinoma. Front Immunol. 2022;13:970117. 35967375 10.3389/fimmu.2022.970117 PMC9363630

[134] Woodle ES , Kulkarni S . Programmed cell death. Transplantation. 1998;66(6):681‐691. 9771830 10.1097/00007890-199809270-00001

[135] Klionsky DJ , Petroni G , Amaravadi RK , et al. Autophagy in major human diseases. EMBO J. 2021;40(19):e108863. 34459017 10.15252/embj.2021108863 PMC8488577

[136] Lorincz P , Juhasz G . Autophagosome‐lysosome fusion. J Mol Biol. 2020;432(8):2462‐2482. 31682838 10.1016/j.jmb.2019.10.028

[137] Miller DR , Thorburn A . Autophagy and organelle homeostasis in cancer. Dev Cell. 2021;56(7):906‐918. 33689692 10.1016/j.devcel.2021.02.010 PMC8026727

[138] Hao W , Dian M , Zhou Y , et al. Autophagy induction promoted by m6A reader YTHDF3 through translation upregulation of FOXO3 mRNA. Nat Commun. 2022;13(1):5845. 36195598 10.1038/s41467-022-32963-0 PMC9532426

[139] Chen X , Wang J , Tahir M , et al. Current insights into the implications of m6A RNA methylation and autophagy interaction in human diseases. Cell Biosci. 2021;11(1):147. 34315538 10.1186/s13578-021-00661-x PMC8314498

[140] Shen M , Li Y , Wang Y , et al. N(6)‐methyladenosine modification regulates ferroptosis through autophagy signaling pathway in hepatic stellate cells. Redox Biol. 2021;47:102151. 34607160 10.1016/j.redox.2021.102151 PMC8495178

[141] Li Q , Ni Y , Zhang L , et al. HIF‐1α‐induced expression of m6A reader YTHDF1 drives hypoxia‐induced autophagy and malignancy of hepatocellular carcinoma by promoting ATG2A and ATG14 translation. Signal Transduct Target Ther. 2021;6(1):76. 33619246 10.1038/s41392-020-00453-8 PMC7900110

[142] Du A , Li S , Zhou Y , et al. M6A‐mediated upregulation of circMDK promotes tumorigenesis and acts as a nanotherapeutic target in hepatocellular carcinoma. Mol Cancer. 2022;21(1):109. 35524319 10.1186/s12943-022-01575-z PMC9074191

[143] Zhang Z , Zhu H , Hu J . CircRAB11FIP1 promoted autophagy flux of ovarian cancer through DSC1 and miR‐129. Cell Death Dis. 2021;12(2):219. 33637694 10.1038/s41419-021-03486-1 PMC7910449

[144] Li G , Deng L , Huang N , et al. m6A mRNA methylation regulates LKB1 to promote autophagy of hepatoblastoma cells through upregulated phosphorylation of AMPK. Genes. 2021;12(11):1747. 34828353 10.3390/genes12111747 PMC8621998

[145] Xu Y , Zhou J , Li L , et al. FTO‐mediated autophagy promotes progression of clear cell renal cell carcinoma via regulating SIK2 mRNA stability. Int J Biol Sci. 2022;18(15):5943‐5962. 36263177 10.7150/ijbs.77774 PMC9576516

[146] Liu Z , Zou H , Dang Q , et al. Biological and pharmacological roles of m(6)A modifications in cancer drug resistance. Mol Cancer. 2022;21(1):220. 36517820 10.1186/s12943-022-01680-z PMC9749187

[147] Jo H , Shim K , Jeoung D . Roles of RNA methylations in cancer progression, autophagy, and anticancer drug resistance. Int J Mol Sci. 2023;24(4):4225. 36835633 10.3390/ijms24044225 PMC9959100

[148] Paramasivam A , Priyadharsini JV . RNA N6‐methyladenosine: a new player in autophagy‐mediated anti‐cancer drug resistance. Br J Cancer. 2021;124(10):1621‐1622. 33723389 10.1038/s41416-021-01314-z PMC8110764

[149] Zhang Y , Gao LX , Wang W , Zhang T , Dong FY , Ding WP . M(6) A demethylase fat mass and obesity‐associated protein regulates cisplatin resistance of gastric cancer by modulating autophagy activation through ULK1. Cancer Sci. 2022;113(9):3085‐3096. 35730319 10.1111/cas.15469 PMC9459343

[150] Sun Y , Shen W , Hu S , et al. METTL3 promotes chemoresistance in small cell lung cancer by inducing mitophagy. J Exp Clin Cancer Res. 2023;42(1):65. 36932427 10.1186/s13046-023-02638-9 PMC10022264

[151] Yang L , Yan B , Qu L , et al. IGF2BP3 regulates TMA7‐mediated autophagy and cisplatin resistance in laryngeal cancer via m6A RNA methylation. Int J Biol Sci. 2023;19(5):1382‐1400. 37056932 10.7150/ijbs.80921 PMC10086756

[152] Lin Z , Niu Y , Wan A , et al. RNA m(6) A methylation regulates sorafenib resistance in liver cancer through FOXO3‐mediated autophagy. EMBO J. 2020;39(12):e103181. 32368828 10.15252/embj.2019103181 PMC7298296

[153] Dixon SJ , Lemberg KM , Lamprecht MR , et al. Ferroptosis: an iron‐dependent form of nonapoptotic cell death. Cell. 2012;149(5):1060‐1072. 22632970 10.1016/j.cell.2012.03.042 PMC3367386

[154] Tang D , Chen X , Kang R , Kroemer G . Ferroptosis: molecular mechanisms and health implications. Cell Res. 2021;31(2):107‐125. 33268902 10.1038/s41422-020-00441-1 PMC8026611

[155] Li J , Cao F , Yin H‐l , et al. Ferroptosis: past, present and future. Cell Death Dis. 2020;11(2):88. 32015325 10.1038/s41419-020-2298-2 PMC6997353

[156] Liu L , He J , Sun G , et al. The N6‐methyladenosine modification enhances ferroptosis resistance through inhibiting SLC7A11 mRNA deadenylation in hepatoblastoma. Clin Transl Med. 2022;12(5):e778. 35522946 10.1002/ctm2.778 PMC9076012

[157] Xu Y , Lv D , Yan C , et al. METTL3 promotes lung adenocarcinoma tumor growth and inhibits ferroptosis by stabilizing SLC7A11 m(6)A modification. Cancer Cell Int. 2022;22(1):11. 34996469 10.1186/s12935-021-02433-6 PMC8742440

[158] Ji FH , Fu XH , Li GQ , He Q , Qiu XG . FTO prevents thyroid cancer progression by SLC7A11 m6A methylation in a ferroptosis‐dependent manner. Front Endocrinol (Lausanne). 2022;13:857765. 35721711 10.3389/fendo.2022.857765 PMC9205202

[159] Luo J , Yu H , Yuan Z , Ye T , Hu B . ALKBH5 decreases SLC7A11 expression by erasing m6A modification and promotes the ferroptosis of colorectal cancer cells. Clin Transl Oncol. 2023;25(7):2265‐2276. 36820954 10.1007/s12094-023-03116-6

[160] Li W , Huang G , Wei J , Cao H , Jiang G . ALKBH5 inhibits thyroid cancer progression by promoting ferroptosis through TIAM1‐Nrf2/HO‐1 axis. Mol Cell Biochem. 2023;478(4):729‐741. 36070054 10.1007/s11010-022-04541-x

[161] Sun S , Gao T , Pang B , et al. RNA binding protein NKAP protects glioblastoma cells from ferroptosis by promoting SLC7A11 mRNA splicing in an m(6)A‐dependent manner. Cell Death Dis. 2022;13(1):73. 35064112 10.1038/s41419-022-04524-2 PMC8783023

[162] Ye F , Wu J , Zhang F . METTL16 epigenetically enhances GPX4 expression via m6A modification to promote breast cancer progression by inhibiting ferroptosis. Biochem Biophys Res Commun. 2023;638:1‐6. 36434904 10.1016/j.bbrc.2022.10.065

[163] Wang S , Wang Y , Li Q , Zeng K , Li X , Feng X . RUNX1‐IT1 favors breast cancer carcinogenesis through regulation of IGF2BP1/GPX4 axis. Discov Oncol. 2023;14(1):42. 37036576 10.1007/s12672-023-00652-z PMC10086083

[164] Shen H , Geng Z , Nie X , Liu T . Erianin induces ferroptosis of renal cancer stem cells via promoting ALOX12/P53 mRNA N6‐methyladenosine modification. J Cancer. 2023;14(3):367‐378. 36860916 10.7150/jca.81027 PMC9969579

[165] Zou Y , Zheng S , Xie X , et al. N6‐methyladenosine regulated FGFR4 attenuates ferroptotic cell death in recalcitrant HER2‐positive breast cancer. Nat Commun. 2022;13(1):2672. 35562334 10.1038/s41467-022-30217-7 PMC9106694

[166] Wang W , Green M , Choi JE , et al. CD8+ T cells regulate tumour ferroptosis during cancer immunotherapy. Nature. 2019;569(7755):270‐274. 31043744 10.1038/s41586-019-1170-y PMC6533917

[167] Kui XY , Gao Y , Liu XS , et al. Comprehensive analysis of SLC17A9 and its prognostic value in hepatocellular carcinoma. Front Oncol. 2022;12:809847. 35957868 10.3389/fonc.2022.809847 PMC9357942

[168] Li J , Tian X , Nie Y , et al. BTBD10 is a prognostic biomarker correlated with immune infiltration in hepatocellular carcinoma. Front Mol Biosci. 2021;8:762541. 35059434 10.3389/fmolb.2021.762541 PMC8764259

[169] Wang Y , Jin P , Wang X . N(6)‐methyladenosine regulator YTHDF1 represses the CD8 + T cell‐mediated antitumor immunity and ferroptosis in prostate cancer via m(6)A/PD‐L1 manner. Apoptosis. 2023. 10.1007/s10495-023-01885-7 37698736

[170] Zha X , Xi X , Fan X , Ma M , Zhang Y , Yang Y . Overexpression of METTL3 attenuates high‐glucose induced RPE cell pyroptosis by regulating miR‐25‐3p/PTEN/Akt signaling cascade through DGCR8. Aging (Albany N Y). 2020;12(9):8137‐8150. 10.18632/aging.103130 PMC7244028 32365051

[171] Zhang S , Guan X , Liu W , et al. YTHDF1 alleviates sepsis by upregulating WWP1 to induce NLRP3 ubiquitination and inhibit caspase‐1‐dependent pyroptosis. Cell Death Discov. 2022;8(1):244. 35508474 10.1038/s41420-022-00872-2 PMC9068740

[172] Dai J , Qu T , Yin D , et al. LncRNA LINC00969 promotes acquired gefitinib resistance by epigenetically suppressing of NLRP3 at transcriptional and posttranscriptional levels to inhibit pyroptosis in lung cancer. Cell Death Dis. 2023;14(5):312. 37156816 10.1038/s41419-023-05840-x PMC10167249

[173] Wu L , Liu G , He YW , Chen R , Wu ZY . Identification of a pyroptosis‐associated long non‐coding RNA signature for predicting the immune status and prognosis in skin cutaneous melanoma. Eur Rev Med Pharmacol Sci. 2021;25(18):5597‐5609. 34604952 10.26355/eurrev_202109_26779

[174] Zhang L , Chu XF , Xu JW , Yao XY , Zhang HQ , Guo YW . Identification and exploration of the pyroptosis‐related molecular subtypes of breast cancer by bioinformatics and machine learning. Am J Transl Res. 2022;14(9):6521‐6535. 36247248 PMC9556502

[175] Lu Z , Tang F , Li Z , et al. Prognosis risk model based on pyroptosis‐related lncRNAs for bladder cancer. Dis Markers. 2022;2022:7931393. 35154513 10.1155/2022/7931393 PMC8828356

[176] Yang P , Yang W , Wei Z , Li Y , Yang Y , Wang J . Novel targets for gastric cancer: the tumor microenvironment (TME), N6‐methyladenosine (m6A), pyroptosis, autophagy, ferroptosis and cuproptosis. Biomed Pharmacother. 2023;163:114883. 37196545 10.1016/j.biopha.2023.114883

[177] Xie J , Yang Y , Gao Y , He J . Cuproptosis: mechanisms and links with cancers. Mol Cancer. 2023;22(1):46. 36882769 10.1186/s12943-023-01732-y PMC9990368

[178] Liu XS , Zeng J , Zhang YH , et al. DARS2 is a prognostic biomarker and correlated with immune infiltrates and cuproptosis in lung adenocarcinoma. Am J Cancer Res. 2023;13(3):818‐834. 37034224 PMC10077054

[179] Gao C , Kong N , Zhang F , Zhou L , Xu M , Wu L . Development and validation of the potential biomarkers based on m6A‐related lncRNAs for the predictions of overall survival in the lung adenocarcinoma and differential analysis with cuproptosis. BMC Bioinformatics. 2022;23(1):327. 35941550 10.1186/s12859-022-04869-7 PMC9358839

[180] Shen L , He Y , Fang C , et al. Cuproptosis‐associated genes and immune microenvironment characterization in breast cancer. Medicine (Baltimore). 2022;101(50):e32301. 36550822 10.1097/MD.0000000000032301 PMC9771175

[181] Peng X , Zhu J , Liu S , et al. Signature construction and molecular subtype identification based on cuproptosis‐related genes to predict the prognosis and immune activity of patients with hepatocellular carcinoma. Front Immunol. 2022;13:990790. 36248822 10.3389/fimmu.2022.990790 PMC9555242

[182] Qin H , Sheng W , Zhang G , et al. Comprehensive analysis of cuproptosis‐related prognostic gene signature and tumor immune microenvironment in HCC. Front Genet. 2023;14:1094793. 36891150 10.3389/fgene.2023.1094793 PMC9986498

[183] Liu X , Nie L , Zhang Y , et al. Actin cytoskeleton vulnerability to disulfide stress mediates disulfidptosis. Nat Cell Biol. 2023;25(3):404‐414. 36747082 10.1038/s41556-023-01091-2 PMC10027392

[184] Zheng T , Liu Q , Xing F , Zeng C , Wang W . Disulfidptosis: a new form of programmed cell death. J Exp Clin Cancer Res. 2023;42(1):137. 37259067 10.1186/s13046-023-02712-2 PMC10230712

[185] Yan Y , Teng H , Hang Q , et al. SLC7A11 expression level dictates differential responses to oxidative stress in cancer cells. Nat Commun. 2023;14(1):3673. 37339981 10.1038/s41467-023-39401-9 PMC10281978

[186] Zheng P , Zhou C , Ding Y , Duan S . Disulfidptosis: a new target for metabolic cancer therapy. J Exp Clin Cancer Res. 2023;42(1):18. 37101248 10.1186/s13046-023-02675-4 PMC10134647

[187] Yang H , Hu Y , Weng M , et al. Hypoxia inducible lncRNA‐CBSLR modulates ferroptosis through m6A‐YTHDF2‐dependent modulation of CBS in gastric cancer. J Adv Res. 2022;37:91‐106. 35499052 10.1016/j.jare.2021.10.001 PMC9039740

[188] Parkin J , Cohen B . An overview of the immune system. Lancet. 2001;357(9270):1777‐1789. 11403834 10.1016/S0140-6736(00)04904-7

[189] Sadighi Akha AA . Aging and the immune system: an overview. J Immunol Methods. 2018;463:21‐26. 30114401 10.1016/j.jim.2018.08.005

[190] Philip M , Schietinger A . CD8(+) T cell differentiation and dysfunction in cancer. Nat Rev Immunol. 2022;22(4):209‐223. 34253904 10.1038/s41577-021-00574-3 PMC9792152

[191] Bi J , Tian Z . NK cell dysfunction and checkpoint immunotherapy. Front Immunol. 2019;10:1999. 31552017 10.3389/fimmu.2019.01999 PMC6736636

[192] Wen JH , Li DY , Liang S , Yang C , Tang JX , Liu HF . Macrophage autophagy in macrophage polarization, chronic inflammation and organ fibrosis. Front Immunol. 2022;13:946832. 36275654 10.3389/fimmu.2022.946832 PMC9583253

[193] Cassetta L , Pollard JW . A timeline of tumour‐associated macrophage biology. Nat Rev Cancer. 2023;23(4):238‐257. 36792751 10.1038/s41568-022-00547-1

[194] Boutilier AJ , Elsawa SF . Macrophage polarization states in the tumor microenvironment. Int J Mol Sci. 2021;22(13):6995. 34209703 10.3390/ijms22136995 PMC8268869

[195] Mantovani A , Allavena P , Marchesi F , Garlanda C . Macrophages as tools and targets in cancer therapy. Nat Rev Drug Discov. 2022;21(11):799‐820. 35974096 10.1038/s41573-022-00520-5 PMC9380983

[196] Tong J , Wang X , Liu Y , et al. Pooled CRISPR screening identifies m(6)A as a positive regulator of macrophage activation. Sci Adv. 2021;7(18):eabd4742. 33910903 10.1126/sciadv.abd4742 PMC8081357

[197] Yin H , Zhang X , Yang P , et al. RNA m6A methylation orchestrates cancer growth and metastasis via macrophage reprogramming. Nat Commun. 2021;12(1):1394. 33654093 10.1038/s41467-021-21514-8 PMC7925544

[198] Wei C , Wang B , Peng D , et al. Pan‐cancer analysis shows that ALKBH5 is a potential prognostic and immunotherapeutic biomarker for multiple cancer types including gliomas. Front Immunol. 2022;13:849592. 35444654 10.3389/fimmu.2022.849592 PMC9013910

[199] Liu Y , Shi M , He X , et al. LncRNA‐PACERR induces pro‐tumour macrophages via interacting with miR‐671‐3p and m6A‐reader IGF2BP2 in pancreatic ductal adenocarcinoma. J Hematol Oncol. 2022;15(1):52. 35526050 10.1186/s13045-022-01272-w PMC9077921

[200] Qin J , Cui Z , Zhou J , et al. IGF2BP3 drives gallbladder cancer progression by m6A‐modified CLDN4 and inducing macrophage immunosuppressive polarization. Transl Oncol. 2023;37:101764. 37643553 10.1016/j.tranon.2023.101764 PMC10472310

[201] Zhang Y , Guo J , Zhang L , et al. CircASPH enhances exosomal STING to facilitate M2 macrophage polarization in colorectal cancer. Inflamm Bowel Dis. 2023. 10.1093/ibd/izad113 37624989

[202] You Y , Wen D , Zeng L , et al. ALKBH5/MAP3K8 axis regulates PD‐L1+ macrophage infiltration and promotes hepatocellular carcinoma progression. Int J Biol Sci. 2022;18(13):5001‐5018. 35982895 10.7150/ijbs.70149 PMC9379398

[203] Zeng X , Chen K , Li L , et al. Epigenetic activation of RBM15 promotes clear cell renal cell carcinoma growth, metastasis and macrophage infiltration by regulating the m6A modification of CXCL11. Free Radic Biol Med. 2022;184:135‐147. 35381326 10.1016/j.freeradbiomed.2022.03.031

[204] Chen J , Zhou Y , Wu M , Yuan Y , Wu W . m6A modification mediates exosomal LINC00657 to trigger breast cancer progression via inducing macrophage M2 polarization. Clin Breast Cancer. 2023;23(5):546‐560. 37198028 10.1016/j.clbc.2023.04.007

[205] McFarlane AJ , Fercoq F , Coffelt SB , Carlin LM . Neutrophil dynamics in the tumor microenvironment. J Clin Invest. 2021;131(6):e143759. 33720040 10.1172/JCI143759 PMC7954585

[206] Luo S , Liao C , Zhang L , et al. METTL3‐mediated m6A mRNA methylation regulates neutrophil activation through targeting TLR4 signaling. Cell Rep. 2023;42(3):112259. 36920907 10.1016/j.celrep.2023.112259

[207] He J , Zhou M , Yin J , et al. METTL3 restrains papillary thyroid cancer progression via m6A/c‐Rel/IL‐8‐mediated neutrophil infiltration. Mol Ther. 2021;29(5):1821‐1837. 33484966 10.1016/j.ymthe.2021.01.019 PMC8116572

[208] Ou B , Liu Y , Gao Z , et al. Senescent neutrophils‐derived exosomal piRNA‐17560 promotes chemoresistance and EMT of breast cancer via FTO‐mediated m6A demethylation. Cell Death Dis. 2022;13(10):905. 36302751 10.1038/s41419-022-05317-3 PMC9613690

[209] Zheng H , Long G , Zheng Y , et al. Glycolysis‐related SLC2A1 is a potential pan‐cancer biomarker for prognosis and immunotherapy. Cancers (Basel). 2022;14(21):5344. 36358765 10.3390/cancers14215344 PMC9657346

[210] Mutua V , Gershwin LJ . A review of neutrophil extracellular traps (NETs) in disease: potential anti‐NETs therapeutics. Clinical Reviews in Allergy (Immunology). 2021;61(2):194‐211. 32740860 10.1007/s12016-020-08804-7 PMC7395212

[211] Masucci MT , Minopoli M , Del Vecchio S , Carriero MV . The emerging role of neutrophil extracellular traps (NETs) in tumor progression and metastasis. Front Immunol. 2020;11:1749. 33042107 10.3389/fimmu.2020.01749 PMC7524869

[212] Lodge KM , Cowburn AS , Li W , Condliffe AM . The impact of hypoxia on neutrophil degranulation and consequences for the host. Int J Mol Sci. 2020;21(4):1183. 32053993 10.3390/ijms21041183 PMC7072819

[213] Burn GL , Foti A , Marsman G , Patel DF , Zychlinsky A . The neutrophil. Immunity. 2021;54(7):1377‐1391. 34260886 10.1016/j.immuni.2021.06.006

[214] Qu M , Chen Z , Qiu Z , et al. Neutrophil extracellular traps‐triggered impaired autophagic flux via METTL3 underlies sepsis‐associated acute lung injury. Cell Death Discov. 2022;8(1):375. 36030287 10.1038/s41420-022-01166-3 PMC9420153

[215] Wang L , Peng J‐L . METTL5 serves as a diagnostic and prognostic biomarker in hepatocellular carcinoma by influencing the immune microenvironment. Sci Rep. 2023;13(1):10755. 37400463 10.1038/s41598-023-37807-5 PMC10318095

[216] Zhang H , Liu J , Zhou Y , et al. Neutrophil extracellular traps mediate m(6)A modification and regulates sepsis‐associated acute lung injury by activating ferroptosis in alveolar epithelial cells. Int J Biol Sci. 2022;18(8):3337‐3357. 35637949 10.7150/ijbs.69141 PMC9134924

[217] Demkow U . Neutrophil extracellular traps (NETs) in cancer invasion, evasion and metastasis. Cancers (Basel). 2021;13(17):4495. 34503307 10.3390/cancers13174495 PMC8431228

[218] Ronchetti L , Boubaker NS , Barba M , Vici P , Gurtner A , Piaggio G . Neutrophil extracellular traps in cancer: not only catching microbes. J Exp Clin Cancer Res. 2021;40(1):231. 34261496 10.1186/s13046-021-02036-z PMC8281578

[219] Kaltenmeier C , Simmons RL , Tohme S , Yazdani HO . Neutrophil extracellular traps (NETs) in cancer metastasis. Cancers (Basel). 2021;13(23):6131. 34885240 10.3390/cancers13236131 PMC8657162

[220] Poto R , Cristinziano L , Modestino L , et al. Neutrophil extracellular traps, angiogenesis and cancer. Biomedicines. 2022;10(2):431. 35203640 10.3390/biomedicines10020431 PMC8962440

[221] Steinman RM , Cohn ZA . Identification of a novel cell type in peripheral lymphoid organs of mice. I. Morphology, quantitation, tissue distribution. J Exp Med. 1973;137(5):1142‐1162. 4573839 10.1084/jem.137.5.1142 PMC2139237

[222] Galati D , Zanotta S . Dendritic cell and cancer therapy. Int J Mol Sci. 2023;24(4):4253. 36835665 10.3390/ijms24044253 PMC9968100

[223] Gardner A , Ruffell B . Dendritic cells and cancer immunity. Trends Immunol. 2016;37(12):855‐865. 27793569 10.1016/j.it.2016.09.006 PMC5135568

[224] Han D , Liu J , Chen C , et al. Anti‐tumour immunity controlled through mRNA m6A methylation and YTHDF1 in dendritic cells. Nature. 2019;566(7743):270‐274. 30728504 10.1038/s41586-019-0916-x PMC6522227

[225] Bai X , Wong CC , Pan Y , et al. Loss of YTHDF1 in gastric tumors restores sensitivity to antitumor immunity by recruiting mature dendritic cells. J Immunother Cancer. 2022;10(2):e003663. 35193930 10.1136/jitc-2021-003663 PMC9066370

[226] Gong PJ , Shao YC , Yang Y , et al. Analysis of N6‐methyladenosine methyltransferase reveals METTL14 and ZC3H13 as tumor suppressor genes in breast cancer. Front Oncol. 2020;10:578963. 33363011 10.3389/fonc.2020.578963 PMC7757663

[227] Zhang YH , Zeng J , Liu XS , et al. ECE2 is a prognostic biomarker associated with m6A modification and involved in immune infiltration of lung adenocarcinoma. Front Endocrinol (Lausanne). 2022;13:1013238. 36299451 10.3389/fendo.2022.1013238 PMC9588963

[228] Shi YL , Liu MB , Wu HT , Han Y , He X . GLTP is a potential prognostic biomarker and correlates with immunotherapy efficacy in cervical cancer. Dis Markers. 2022;2022:9109365. 35845139 10.1155/2022/9109365 PMC9282991

[229] Li Y , Zheng JN , Wang EH , Gong CJ , Lan KF , Ding X . The m6A reader protein YTHDC2 is a potential biomarker and associated with immune infiltration in head and neck squamous cell carcinoma. PeerJ. 2020;8:e10385. 33304653 10.7717/peerj.10385 PMC7700739

[230] Kumar BV , Connors TJ , Farber DL . Human T cell development, localization, and function throughout life. Immunity. 2018;48(2):202‐213. 29466753 10.1016/j.immuni.2018.01.007 PMC5826622

[231] Wherry EJ , Kurachi M . Molecular and cellular insights into T cell exhaustion. Nat Rev Immunol. 2015;15(8):486‐499. 26205583 10.1038/nri3862 PMC4889009

[232] Henning AN , Roychoudhuri R , Restifo NP . Epigenetic control of CD8(+) T cell differentiation. Nat Rev Immunol. 2018;18(5):340‐356. 29379213 10.1038/nri.2017.146 PMC6327307

[233] Chao Y , Li HB , Zhou J . Multiple functions of RNA methylation in T cells: a review. Front Immunol. 2021;12:627455. 33912158 10.3389/fimmu.2021.627455 PMC8071866

[234] Liu Z , Wang T , She Y , et al. N(6)‐methyladenosine‐modified circIGF2BP3 inhibits CD8(+) T‐cell responses to facilitate tumor immune evasion by promoting the deubiquitination of PD‐L1 in non‐small cell lung cancer. Mol Cancer. 2021;20(1):105. 34416901 10.1186/s12943-021-01398-4 PMC8377850

[235] Wan W , Ao X , Chen Q , et al. METTL3/IGF2BP3 axis inhibits tumor immune surveillance by upregulating N(6)‐methyladenosine modification of PD‐L1 mRNA in breast cancer. Mol Cancer. 2022;21(1):60. 35197058 10.1186/s12943-021-01447-y PMC8864846

[236] Dong L , Chen C , Zhang Y , et al. The loss of RNA N(6)‐adenosine methyltransferase Mettl14 in tumor‐associated macrophages promotes CD8(+) T cell dysfunction and tumor growth. Cancer Cell. 2021;39(7):945‐957. e10. 34019807 10.1016/j.ccell.2021.04.016

[237] Chen H , Pan Y , Zhou Q , et al. METTL3 inhibits antitumor immunity by targeting m(6)A‐BHLHE41‐CXCL1/CXCR2 axis to promote colorectal cancer. Gastroenterology. 2022;163(4):891‐907. 35700773 10.1053/j.gastro.2022.06.024

[238] Bao Y , Zhai J , Chen H , et al. Targeting m(6)A reader YTHDF1 augments antitumour immunity and boosts anti‐PD‐1 efficacy in colorectal cancer. Gut. 2023;72(8):1497‐1509. 36717220 10.1136/gutjnl-2022-328845 PMC10359538

[239] Hu Z , Chen G , Zhao Y , et al. Exosome‐derived circCCAR1 promotes CD8 + T‐cell dysfunction and anti‐PD1 resistance in hepatocellular carcinoma. Mol Cancer. 2023;22(1):55. 36932387 10.1186/s12943-023-01759-1 PMC10024440

[240] Li T , Tan YT , Chen YX , et al. Methionine deficiency facilitates antitumour immunity by altering m(6)A methylation of immune checkpoint transcripts. Gut. 2023;72(3):501‐511. 35803704 10.1136/gutjnl-2022-326928 PMC9933173

[241] Zhang L , Li Y , Zhou L , et al. The m6A reader YTHDF2 promotes bladder cancer progression by suppressing RIG‐I‐mediated immune response. Cancer Res. 2023;83(11):1834‐1850. 36939388 10.1158/0008-5472.CAN-22-2485 PMC10236158

[242] Cheng Y , Li L , Wei X , et al. HNRNPC suppresses tumor immune microenvironment by activating Treg cells promoting the progression of prostate cancer. Cancer Sci. 2023;114(5):1830‐1845. 36718950 10.1111/cas.15745 PMC10154801

[243] Zhang Z , Tan X , Wu R , et al. m6A‐mediated upregulation of lncRNA‐AC026356.1 promotes cancer stem cell maintenance in lung adenocarcinoma via activating Wnt signaling pathway. Aging. 2023;15(9):3538‐3548. 37142269 10.18632/aging.204689 PMC10449284

[244] Sattiraju A , Kang S , Giotti B , et al. Hypoxic niches attract and sequester tumor‐associated macrophages and cytotoxic T cells and reprogram them for immunosuppression. Immunity. 2023. 10.1016/j.immuni.2023.06.017 PMC10527169 37451265

[245] Shen X , Zhong J , He J , Han J , Chen N . Identification of m6A modification patterns and development of m6A‐hypoxia prognostic signature to characterize tumor microenvironment in triple‐negative breast cancer. Front Immunol. 2022;13:978092. 36105819 10.3389/fimmu.2022.978092 PMC9465332

[246] Cyster JG , Allen CDC . B cell responses: cell interaction dynamics and decisions. Cell. 2019;177(3):524‐540. 31002794 10.1016/j.cell.2019.03.016 PMC6538279

[247] LeBien TW , Tedder TF . B lymphocytes: how they develop and function. Blood. 2008;112(5):1570‐1580. 18725575 10.1182/blood-2008-02-078071 PMC2518873

[248] Zheng Z , Zhang L , Cui X‐L , et al. Control of early B cell development by the RNA N6‐methyladenosine methylation. Cell Rep. 2020;31(13):107819. 32610122 10.1016/j.celrep.2020.107819 PMC7371152

[249] Zhao C , Xu G , Zhang X , Ye Y , Cai W , Shao Q . RNA m(6)A modification orchestrates the rhythm of immune cell development from hematopoietic stem cells to T and B cells. Front Immunol. 2022;13:839291. 35935968 10.3389/fimmu.2022.839291 PMC9354743

[250] Kang X , Chen S , Pan L , et al. Deletion of Mettl3 at the Pro‐B stage marginally affects B cell development and profibrogenic activity of B cells in liver fibrosis. J Immunol Res. 2022;2022:1‐17. 10.1155/2022/8118577 PMC9213183 35747688

[251] Grenov A , Hezroni H , Lasman L , Hanna JH , Shulman Z . YTHDF2 suppresses the plasmablast genetic program and promotes germinal center formation. Cell Rep. 2022;39(5):110778. 35508130 10.1016/j.celrep.2022.110778 PMC9108551

[252] Han H , Fan G , Song S , et al. piRNA‐30473 contributes to tumorigenesis and poor prognosis by regulating m6A RNA methylation in DLBCL. Blood. 2021;137(12):1603‐1614. 32967010 10.1182/blood.2019003764

[253] Cheng Y , Fu Y , Wang Y , Wang J . The m6A methyltransferase METTL3 is functionally implicated in DLBCL development by regulating m6A modification in PEDF. Front Genet. 2020;11:955. 33061938 10.3389/fgene.2020.00955 PMC7481464

[254] Meng S , Xia Y , Li M , et al. NCBP1 enhanced proliferation of DLBCL cells via METTL3‐mediated m6A modification of c‐Myc. Sci Rep. 2023;13(1):8606. 37244946 10.1038/s41598-023-35777-2 PMC10224985

[255] Chen X , Lu T , Cai Y , et al. KIAA1429‐mediated m6A modification of CHST11 promotes progression of diffuse large B‐cell lymphoma by regulating Hippo‐YAP pathway. Cell Mol Biol Lett. 2023;28(1):32. 37076815 10.1186/s11658-023-00445-w PMC10114474

[256] Laskowski TJ , Biederstädt A , Rezvani K . Natural killer cells in antitumour adoptive cell immunotherapy. Nat Rev Cancer. 2022;22(10):557‐575. 35879429 10.1038/s41568-022-00491-0 PMC9309992

[257] Myers JA , Miller JS . Exploring the NK cell platform for cancer immunotherapy. Nat Rev Clin Oncol. 2021;18(2):85‐100. 32934330 10.1038/s41571-020-0426-7 PMC8316981

[258] Liu S , Galat V , Galat Y , Lee YKA , Wainwright D , Wu J . NK cell‐based cancer immunotherapy: from basic biology to clinical development. J Hematol Oncol. 2021;14(1):7. 33407739 10.1186/s13045-020-01014-w PMC7788999

[259] Wu SY , Fu T , Jiang YZ , Shao ZM . Natural killer cells in cancer biology and therapy. Mol Cancer. 2020;19(1):120. 32762681 10.1186/s12943-020-01238-x PMC7409673

[260] Liu XS , Zhou LM , Yuan LL , et al. NPM1 is a prognostic biomarker involved in immune infiltration of lung adenocarcinoma and associated with m6A modification and glycolysis. Front Immunol. 2021;12:724741. 34335635 10.3389/fimmu.2021.724741 PMC8324208

[261] Xie H , Shi M , Liu Y , et al. Identification of m6A‐ and ferroptosis‐related lncRNA signature for predicting immune efficacy in hepatocellular carcinoma. Front Immunol. 2022;13:914977. 36032107 10.3389/fimmu.2022.914977 PMC9402990

[262] Yu H , Wang C , Ke S , et al. Identification of CFHR4 as a potential prognosis biomarker associated with lmmune infiltrates in hepatocellular carcinoma. Front Immunol. 2022;13:892750. 35812416 10.3389/fimmu.2022.892750 PMC9257081

[263] Li Z , Li Y , Zhong W , Huang P . m6A‐related lncRNA to develop prognostic signature and predict the immune landscape in bladder cancer. J Oncol. 2021;2021:7488188. 34349798 10.1155/2021/7488188 PMC8328735

[264] Xu Z , Chen Q , Shu L , Zhang C , Liu W , Wang P . Expression profiles of m6A RNA methylation regulators, PD‐L1 and immune infiltrates in gastric cancer. Front Oncol. 2022;12:970367. 36003776 10.3389/fonc.2022.970367 PMC9393729

[265] Wang E , Li Y , Ming R , et al. The prognostic value and immune landscapes of a m(6)A/m(5)C/m(1)A‐related LncRNAs signature in head and neck squamous cell carcinoma. Front Cell Dev Biol. 2021;9:718974. 34917609 10.3389/fcell.2021.718974 PMC8670092

[266] Ma S , Yan J , Barr T , et al. The RNA m6A reader YTHDF2 controls NK cell antitumor and antiviral immunity. J Exp Med. 2021;218(8):e20210279. 34160549 10.1084/jem.20210279 PMC8225680

[267] Zhu G , Xie J , Kong W , et al. Phase separation of disease‐associated SHP2 mutants underlies MAPK hyperactivation. Cell. 2020;183(2):490‐502. 33002410 10.1016/j.cell.2020.09.002 PMC7572904

[268] Song H , Song J , Cheng M , et al. METTL3‐mediated m(6)A RNA methylation promotes the anti‐tumour immunity of natural killer cells. Nat Commun. 2021;12(1):5522. 34535671 10.1038/s41467-021-25803-0 PMC8448775

[269] Sholl J , Sepich‐Poore GD , Knight R , Pradeu T . Redrawing therapeutic boundaries: microbiota and cancer. Trends Cancer. 2022;8(2):87‐97. 34844910 10.1016/j.trecan.2021.10.008 PMC8770609

[270] Wong‐Rolle A , Wei HK , Zhao C , Jin C . Unexpected guests in the tumor microenvironment: microbiome in cancer. Protein Cell. 2021;12(5):426‐435. 33296049 10.1007/s13238-020-00813-8 PMC8106554

[271] Qiu FS , He JQ , Zhong YS , Guo MY , Yu CH . Implications of m6A methylation and microbiota interaction in non‐small cell lung cancer: from basics to therapeutics. Front Cell Infect Microbiol. 2022;12:972655. 36118041 10.3389/fcimb.2022.972655 PMC9478539

[272] Waldum H , Gastritis FossmarkR . Gastric polyps and gastric cancer. Int J Mol Sci. 2021;22(12):6548. 34207192 10.3390/ijms22126548 PMC8234857

[273] Salvatori S , Marafini I , Laudisi F , Monteleone G , Stolfi C . Helicobacter pylori and gastric cancer: pathogenetic mechanisms. Int J Mol Sci. 2023;24(3):2895. 36769214 10.3390/ijms24032895 PMC9917787

[274] Li H , Lin J , Cheng S , et al. Comprehensive analysis of differences in N6‐methyladenosine RNA methylomes in Helicobacter pylori infection. Front Cell Dev Biol. 2023;11:1136096. 37363723 10.3389/fcell.2023.1136096 PMC10289286

[275] Cheng S , Li H , Chi J , et al. FTO‐mediated m(6)A modification promotes malignant transformation of gastric mucosal epithelial cells in chronic Cag A(+) Helicobacter pylori infection. J Cancer Res Clin Oncol. 2023;149(10):7327‐7340. 36918410 10.1007/s00432-023-04684-4 PMC10374804

[276] Oster P , Vaillant L , Riva E , et al. Helicobacter pylori infection has a detrimental impact on the efficacy of cancer immunotherapies. Gut. 2022;71(3):457‐466. 34253574 10.1136/gutjnl-2020-323392 PMC8862014

[277] Holokai L , Chakrabarti J , Broda T , et al. Increased programmed death‐ligand 1 is an early epithelial cell response to helicobacter pylori infection. PLOS Pathogens. 2019;15(1):e1007468. 30703170 10.1371/journal.ppat.1007468 PMC6380601

[278] Brennan CA , Garrett WS . Fusobacterium nucleatum—symbiont, opportunist and oncobacterium. Nat Rev Microbiol. 2019;17(3):156‐166. 30546113 10.1038/s41579-018-0129-6 PMC6589823

[279] Alon‐Maimon T , Mandelboim O , Bachrach G . Fusobacterium nucleatum and cancer. Periodontol 2000. 2022;89(1):166‐180. 35244982 10.1111/prd.12426 PMC9315032

[280] Kostic AD , Chun E , Robertson L , et al. Fusobacterium nucleatum potentiates intestinal tumorigenesis and modulates the tumor‐immune microenvironment. Cell Host Microbe. 2013;14(2):207‐215. 23954159 10.1016/j.chom.2013.07.007 PMC3772512

[281] Chen S , Zhang L , Li M , et al. Fusobacterium nucleatum reduces METTL3‐mediated m(6)A modification and contributes to colorectal cancer metastasis. Nat Commun. 2022;13(1):1248. 35273176 10.1038/s41467-022-28913-5 PMC8913623

[282] Xu Q , Lu X , Li J , et al. Fusobacterium nucleatum induces excess methyltransferase‐like 3‐mediated microRNA‐4717‐3p maturation to promote colorectal cancer cell proliferation. Cancer Sci. 2022;113(11):3787‐3800. 35984699 10.1111/cas.15536 PMC9633291

[283] Guo S , Chen F , Li L , et al. Intracellular Fusobacterium nucleatum infection increases METTL3‐mediated m6A methylation to promote the metastasis of esophageal squamous cell carcinoma. J Adv Res. 2023. 10.1016/j.jare.2023.08.014 PMC11258656 37619934

[284] Gao Y , Zou T , Xu P , et al. Fusobacterium nucleatum stimulates cell proliferation and promotes PD‐L1 expression via IFIT1‐related signal in colorectal cancer. Neoplasia. 2023;35:100850. 36371909 10.1016/j.neo.2022.100850 PMC9664554

[285] Hatzakis A , Magiorkinis E , Haida C . HBV virological assessment. J Hepatol. 2006;44(1):S71‐S76. Suppl. 16343681 10.1016/j.jhep.2005.11.017

[286] Murata T , Iwahori S , Okuno Y , et al. N6‐methyladenosine modification of hepatitis B virus RNA in the Coding Region of HBx. Int J Mol Sci. 2023;24(3):2265. 36768585 10.3390/ijms24032265 PMC9917364

[287] Imam H , Khan M , Gokhale NS , et al. N6‐methyladenosine modification of hepatitis B virus RNA differentially regulates the viral life cycle. Proc Natl Acad Sci U S A. 2018;115(35):8829‐8834. 30104368 10.1073/pnas.1808319115 PMC6126736

[288] Kim GW , Siddiqui A . Hepatitis B virus X protein expression is tightly regulated by N6‐methyladenosine modification of its mRNA. J Virol. 2022;96(4):e0165521. 34851655 10.1128/jvi.01655-21 PMC8865537

[289] Ding WB , Wang MC , Yu J , et al. HBV/pregenomic RNA increases the stemness and promotes the development of HBV‐related HCC Through reciprocal regulation with insulin‐like growth factor 2 mRNA‐binding protein 3. Hepatology. 2021;74(3):1480‐1495. 33825218 10.1002/hep.31850

[290] Kim GW , Imam H , Khan M , et al. HBV‐induced increased N6 methyladenosine modification of PTEN RNA affects innate immunity and contributes to HCC. Hepatology. 2021;73(2):533‐547. 32394474 10.1002/hep.31313 PMC7655655

[291] Kim GW , Imam H , Siddiqui A . The RNA binding proteins YTHDC1 and FMRP regulate the nuclear export of N(6)‐methyladenosine‐modified hepatitis B Virus Transcripts and Affect the Viral Life Cycle. J Virol. 2021;95(13):e0009721. 33883220 10.1128/JVI.00097-21 PMC8316146

[292] Zhang Z , Gao W , Liu Z , et al. Comprehensive analysis of m6A regulators associated with immune infiltration in Hepatitis B virus‐related hepatocellular carcinoma. BMC Gastroenterol. 2023;23(1):259. 37507670 10.1186/s12876-023-02873-6 PMC10385918

[293] Zhao T , Qi J , Liu T , Wu H , Zhu Q . N6‐methyladenosine modification participates in the progression of hepatitis B virus‐related liver fibrosis by regulating immune cell infiltration. Front Med (Lausanne). 2022;9:821710. 35308519 10.3389/fmed.2022.821710 PMC8924664

[294] Kanda T , Yajima M , Ikuta K . Epstein‐Barr virus strain variation and cancer. Cancer Sci. 2019;110(4):1132‐1139. 30697862 10.1111/cas.13954 PMC6447851

[295] Xia TL , Li X , Wang X , et al. N(6)‐methyladenosine‐binding protein YTHDF1 suppresses EBV replication and promotes EBV RNA decay. EMBO Rep. 2021;22(4):e50128. 33605073 10.15252/embr.202050128 PMC8025027

[296] Bose D , Lin X , Gao L , Wei Z , Pei Y , Robertson ES . Attenuation of IFN signaling due to m(6)A modification of the host epitranscriptome promotes EBV lytic reactivation. J Biomed Sci. 2023;30(1):18. 36918845 10.1186/s12929-023-00911-9 PMC10012557

[297] Guo R , Gewurz BE . Epigenetic control of the Epstein‐Barr lifecycle. Curr Opin Virol. 2022;52:78‐88. 34891084 10.1016/j.coviro.2021.11.013 PMC9112224

[298] Zheng X , Wang J , Zhang X , et al. RNA m(6) A methylation regulates virus‐host interaction and EBNA2 expression during Epstein‐Barr virus infection. Immun Inflamm Dis. 2021;9(2):351‐362. 33434416 10.1002/iid3.396 PMC8127537

[299] Lang F , Singh RK , Pei Y , Zhang S , Sun K , Robertson ES . EBV epitranscriptome reprogramming by METTL14 is critical for viral‐associated tumorigenesis. PLoS Pathog. 2019;15(6):e1007796. 31226160 10.1371/journal.ppat.1007796 PMC6588254

[300] Xiao H , Zhang Y , Sun L , Zhao Z , Liu W , Luo B . EBV downregulates the m(6)A “writer” WTAP in EBV‐associated gastric carcinoma. Virus Res. 2021;304:198510. 34329695 10.1016/j.virusres.2021.198510

[301] Liu Z , He J , Han J , Yang J , Liao W , Chen N . m6A regulators mediated methylation modification patterns and tumor microenvironment infiltration characterization in nasopharyngeal carcinoma. Front Immunol. 2021;12:762243. 35069534 10.3389/fimmu.2021.762243 PMC8776994

[302] Zhang JY , Du Y , Gong LP , et al. ebv‐circRPMS1 promotes the progression of EBV‐associated gastric carcinoma via Sam68‐dependent activation of METTL3. Cancer Lett. 2022;535:215646. 35304258 10.1016/j.canlet.2022.215646

[303] Stanley M . Pathology and epidemiology of HPV infection in females. Gynecol Oncol. 2010;117(2):S5‐S10. Suppl. 20304221 10.1016/j.ygyno.2010.01.024

[304] Roman BR , Aragones A . Epidemiology and incidence of HPV‐related cancers of the head and neck. J Surg Oncol. 2021;124(6):920‐922. 34558067 10.1002/jso.26687 PMC8552291

[305] Araldi RP , Sant'Ana TA , Módolo DG , et al. The human papillomavirus (HPV)‐related cancer biology: an overview. Biomed Pharmacother. 2018;106:1537‐1556. 30119229 10.1016/j.biopha.2018.06.149

[306] Hoppe‐Seyler K , Bossler F , Braun JA , Herrmann AL , Hoppe‐Seyler F . The HPV E6/E7 oncogenes: key factors for viral carcinogenesis and therapeutic targets. Trends Microbiol. 2018;26(2):158‐168. 28823569 10.1016/j.tim.2017.07.007

[307] Hu C , Liu T , Han C , et al. HPV E6/E7 promotes aerobic glycolysis in cervical cancer by regulating IGF2BP2 to stabilize m(6)A‐MYC expression. Int J Biol Sci. 2022;18(2):507‐521. 35002506 10.7150/ijbs.67770 PMC8741847

[308] Wang L , Zhan G , Maimaitiyiming Y , et al. m(6)A modification confers thermal vulnerability to HPV E7 oncotranscripts via reverse regulation of its reader protein IGF2BP1 upon heat stress. Cell Rep. 2022;41(4):111546. 36288717 10.1016/j.celrep.2022.111546

[309] Huo FC , Zhu ZM , Du WQ , et al. HPV E7‐drived ALKBH5 promotes cervical cancer progression by modulating m6A modification of PAK5. Pharmacol Res. 2023;195:106863. 37480971 10.1016/j.phrs.2023.106863

[310] Gu Z , Liu J , Qin L , et al. WTAP‐mediated m6A modification of IFNE is required for antiviral defense in condyloma acuminata. J Dermatol Sci. 2023;111(2):43‐51. 37516644 10.1016/j.jdermsci.2023.07.004

[311] Yu R , Wei Y , He C , et al. Integrative analyses of m6A regulators identify that METTL3 is associated with HPV status and immunosuppressive microenvironment in HPV‐related cancers. Int J Biol Sci. 2022;18(9):3874‐3887. 35813476 10.7150/ijbs.70674 PMC9254478

[312] Grossmann KF , Margolin K . Long‐term survival as a treatment benchmark in melanoma: latest results and clinical implications. Ther Adv Med Oncol. 2015;7(3):181‐191. 26673806 10.1177/1758834015572284 PMC4406913

[313] Liu Z , Zhong J , Zeng J , et al. Characterization of the m6A‐associated tumor immune microenvironment in prostate cancer to aid immunotherapy. Front Immunol. 2021;12:735170. 34531875 10.3389/fimmu.2021.735170 PMC8438522

[314] Shriwas O , Mohapatra P , Mohanty S , Dash R . The impact of m6A RNA modification in therapy resistance of cancer: implication in chemotherapy, radiotherapy, and immunotherapy. Front Oncol. 2020;10:612337. 33718113 10.3389/fonc.2020.612337 PMC7947626

[315] Barbieri I , Kouzarides T . Role of RNA modifications in cancer. Nat Rev Cancer. 2020;20(6):303‐322. 32300195 10.1038/s41568-020-0253-2

[316] Wang Z , Zhou J , Zhang H , Ge L , Li J , Wang H . RNA m(6) A methylation in cancer. Mol Oncol. 2023;17(2):195‐229. 36260366 10.1002/1878-0261.13326 PMC9892831

[317] Mehdi A , Rabbani SA . Role of methylation in pro‐ and anti‐cancer immunity. Cancers (Basel). 2021;13(3):545. 33535484 10.3390/cancers13030545 PMC7867049

[318] Polack FP , Thomas SJ , Kitchin N , et al. Safety and efficacy of the BNT162b2 mRNA Covid‐19 vaccine. N Engl J Med. 2020;383(27):2603‐2615. 33301246 10.1056/NEJMoa2034577 PMC7745181

[319] Lorentzen CL , Haanen JB , Met Ö , Svane IM . Clinical advances and ongoing trials on mRNA vaccines for cancer treatment. Lancet Oncol. 2022;23(10):e450‐e458. 36174631 10.1016/S1470-2045(22)00372-2 PMC9512276

[320] Miao L , Zhang Y , Huang L . mRNA vaccine for cancer immunotherapy. Mol Cancer. 2021;20(1):41. 33632261 10.1186/s12943-021-01335-5 PMC7905014

[321] Morse MA , Gwin WR 3rd , Mitchell DA . Vaccine therapies for cancer: then and now. Target Oncol. 2021;16(2):121‐152. 33512679 10.1007/s11523-020-00788-w PMC7845582

[322] Chen J , Chen J , Xu Q . Current developments and challenges of mRNA vaccines. Annu Rev Biomed Eng. 2022;24:85‐109. 35231177 10.1146/annurev-bioeng-110220-031722

[323] Karikó K , Buckstein M , Ni H , Weissman D . Suppression of RNA recognition by Toll‐like receptors: the impact of nucleoside modification and the evolutionary origin of RNA. Immunity. 2005;23(2):165‐175. 16111635 10.1016/j.immuni.2005.06.008

[324] Kim SC , Sekhon SS , Shin WR , et al. Modifications of mRNA vaccine structural elements for improving mRNA stability and translation efficiency. Mol Cell Toxicol. 2022;18(1):1‐8. 34567201 10.1007/s13273-021-00171-4 PMC8450916

[325] He PC , Wei J , Dou X , et al. Exon architecture controls mRNA m(6)A suppression and gene expression. Science. 2023;379(6633):677‐682. 36705538 10.1126/science.abj9090 PMC9990141

[326] Peng PH , Hsu KW , Wu KJ . Liquid‐liquid phase separation (LLPS) in cellular physiology and tumor biology. Am J Cancer Res. 2021;11(8):3766‐3776. 34522448 PMC8414392

[327] Mehta S , Zhang J . Liquid‐liquid phase separation drives cellular function and dysfunction in cancer. Nat Rev Cancer. 2022;22(4):239‐252. 35149762 10.1038/s41568-022-00444-7 PMC10036213

[328] Lee JH , Wang R , Xiong F , et al. Enhancer RNA m6A methylation facilitates transcriptional condensate formation and gene activation. Mol Cell. 2021;81(16):3368‐3385. 34375583 10.1016/j.molcel.2021.07.024 PMC8383322

[329] Patil A , Strom AR , Paulo JA , et al. A disordered region controls cBAF activity via condensation and partner recruitment. Cell. 2023. 10.1016/j.cell.2023.08.032 PMC10792396 37788668

[330] Cheng Y , Xie W , Pickering BF , et al. N(6)‐Methyladenosine on mRNA facilitates a phase‐separated nuclear body that suppresses myeloid leukemic differentiation. Cancer Cell. 2021;39(7):958‐972. 34048709 10.1016/j.ccell.2021.04.017 PMC8282764

[331] Li J , Chen K , Dong X , et al. YTHDF1 promotes mRNA degradation via YTHDF1‐AGO2 interaction and phase separation. Cell Prolif. 2022;55(1):e13157. 34821414 10.1111/cpr.13157 PMC8780909

[332] Cargill T , Barnes E . Therapeutic vaccination for treatment of chronic hepatitis B. Clin Exp Immunol. 2021;205(2):106‐118. 33969474 10.1111/cei.13614 PMC8274149

[333] Lee HW , Lee JS , Ahn SH . Hepatitis B Virus Cure: targets and Future Therapies. Int J Mol Sci. 2020;22(1):213. 33379331 10.3390/ijms22010213 PMC7795643

[334] Patrizia G , Ilaria P , Orazio F , Francesca P . Immunomodulatory role of EV‐derived non‐coding RNA in lung cancer. Extracell Vesicles Circ Nucl Acids. 2023;4(1):59‐71. 10.20517/evcna.2022.42 PMC11648522 39698297

[335] Cheng C , Wang P , Yang Y , et al. Smoking‐Induced M2‐TAMs, via circEML4 in EVs, Promote the progression of NSCLC through ALKBH5‐regulated m6A modification of SOCS2 in NSCLC cells. Adv Sci (Weinh). 2023:e2300953. 37246269 10.1002/advs.202300953 PMC10401136

[336] You Q , Wang F , Du R , et al. m(6) A reader YTHDF1‐targeting engineered small extracellular vesicles for gastric cancer therapy via epigenetic and immune regulation. Adv Mater. 2023;35(8):e2204910. 36484103 10.1002/adma.202204910

[337] Kalluri R , LeBleu VS . The biology, function, and biomedical applications of exosomes. Science. 2020;367(6478):eaau6977. 32029601 10.1126/science.aau6977 PMC7717626

[338] Ye Y , Wang M , Wang G , et al. lncRNA miR4458HG modulates hepatocellular carcinoma progression by activating m6A‐dependent glycolysis and promoting the polarization of tumor‐associated macrophages. Cell Mol Life Sci. 2023;80(4):99. 36933158 10.1007/s00018-023-04741-8 PMC11072995

[339] Xie H , Yao J , Wang Y , Ni B . Exosome‐transmitted circVMP1 facilitates the progression and cisplatin resistance of non‐small cell lung cancer by targeting miR‐524‐5p‐METTL3/SOX2 axis. Drug Deliv. 2022;29(1):1257‐1271. 35467477 10.1080/10717544.2022.2057617 PMC9045767

[340] Li H , Peng K , Yang K , et al. Circular RNA cancer vaccines drive immunity in hard‐to‐treat malignancies. Theranostics. 2022;12(14):6422‐6436. 36168634 10.7150/thno.77350 PMC9475446

[341] Johnson DB , Nebhan CA , Moslehi JJ , Balko JM . Immune‐checkpoint inhibitors: long‐term implications of toxicity. Nat Rev Clin Oncol. 2022;19(4):254‐267. 35082367 10.1038/s41571-022-00600-w PMC8790946

[342] Kornepati AVR , Vadlamudi RK , Curiel TJ . Programmed death ligand 1 signals in cancer cells. Nat Rev Cancer. 2022;22(3):174‐189. 35031777 10.1038/s41568-021-00431-4 PMC9989967

[343] Schadendorf D , Fisher DE , Garbe C , et al. Melanoma. Nat Rev Dis Primers. 2015;1:15003. 27188223 10.1038/nrdp.2015.3

[344] Tang Q , Chen Y , Li X , et al. The role of PD‐1/PD‐L1 and application of immune‐checkpoint inhibitors in human cancers. Front Immunol. 2022;13:964442. 36177034 10.3389/fimmu.2022.964442 PMC9513184

[345] Liu L , Liang L , Li H , et al. The role of m6A‐mediated PD‐1/PD‐L1 in antitumor immunity. Biochem Pharmacol. 2023;210:115460. 36822438 10.1016/j.bcp.2023.115460

[346] Peng L , Pan B , Zhang X , et al. Lipopolysaccharide facilitates immune escape of hepatocellular carcinoma cells via m6A modification of lncRNA MIR155HG to upregulate PD‐L1 expression. Cell Biol Toxicol. 2022;38(6):1159‐1173. 35438468 10.1007/s10565-022-09718-0

[347] Tang W , Xu N , Zhou J , et al. ALKBH5 promotes PD‐L1‐mediated immune escape through m6A modification of ZDHHC3 in glioma. Cell Death Discov. 2022;8(1):497. 36566230 10.1038/s41420-022-01286-w PMC9789960

[348] Song Z , Wang X , Chen F , et al. LncRNA MALAT1 regulates METTL3‐mediated PD‐L1 expression and immune infiltrates in pancreatic cancer. Front Oncol. 2022;12:1004212. 36212476 10.3389/fonc.2022.1004212 PMC9533337

[349] Wang A , Sun Y , Wang X , et al. m(6)A methyltransferase METTL16 mediates immune evasion of colorectal cancer cells via epigenetically regulating PD‐L1 expression. Aging (Albany N Y). 2023;15(16):8444‐8457. 10.18632/aging.204980 PMC10496997 37647025

[350] Qiu X , Yang S , Wang S , et al. M(6)A demethylase ALKBH5 regulates PD‐L1 expression and tumor immunoenvironment in intrahepatic cholangiocarcinoma. Cancer Res. 2021;81(18):4778‐4793. 34301762 10.1158/0008-5472.CAN-21-0468

[351] Yan G , An Y , Xu B , Wang N , Sun X , Sun M . Potential impact of ALKBH5 and YTHDF1 on tumor immunity in colon adenocarcinoma. Front Oncol. 2021;11:670490. 34079761 10.3389/fonc.2021.670490 PMC8165310

[352] Vesely MD , Zhang T , Chen L . Resistance mechanisms to Anti‐PD cancer immunotherapy. Annu Rev Immunol. 2022;40:45‐74. 35471840 10.1146/annurev-immunol-070621-030155

[353] Wang L , Hui H , Agrawal K , et al. m(6) A RNA methyltransferases METTL3/14 regulate immune responses to anti‐PD‐1 therapy. EMBO J. 2020;39(20):e104514. 32964498 10.15252/embj.2020104514 PMC7560214

[354] Yang S , Wei J , Cui YH , et al. m(6)A mRNA demethylase FTO regulates melanoma tumorigenicity and response to anti‐PD‐1 blockade. Nat Commun. 2019;10(1):2782. 31239444 10.1038/s41467-019-10669-0 PMC6592937

[355] Zhai J , Chen H , Wong CC , et al. ALKBH5 drives immune suppression via targeting AXIN2 to promote colorectal cancer and is a target for boosting immunotherapy. Gastroenterology. 2023;165(2):445‐462. 37169182 10.1053/j.gastro.2023.04.032

[356] Pan Y , Chen H , Zhang X , et al. METTL3 drives NAFLD‐related hepatocellular carcinoma and is a therapeutic target for boosting immunotherapy. Cell Rep Med. 2023;4(8):101144. 37586322 10.1016/j.xcrm.2023.101144 PMC10439254

[357] Kubli SP , Berger T , Araujo DV , Siu LL , Mak TW . Beyond immune checkpoint blockade: emerging immunological strategies. Nat Rev Drug Discov. 2021;20(12):899‐919. 33686237 10.1038/s41573-021-00155-y

[358] Han J , Liu Y , Yang S , Wu X , Li H , Wang Q . MEK inhibitors for the treatment of non‐small cell lung cancer. J Hematol Oncol. 2021;14(1):1. 33402199 10.1186/s13045-020-01025-7 PMC7786519

[359] Chauvin JM , Zarour HM . TIGIT in cancer immunotherapy. J Immunother Cancer. 2020;8(2):e000957. 32900861 10.1136/jitc-2020-000957 PMC7477968

[360] Kim D , Lee SA , Moon H , Kim K , Park D . The Tim gene family in efferocytosis. Genes Genomics. 2020;42(9):979‐986. 32648232 10.1007/s13258-020-00969-x

[361] Liu Z , Zheng N , Li J , et al. N6‐methyladenosine‐modified circular RNA QSOX1 promotes colorectal cancer resistance to anti‐CTLA‐4 therapy through induction of intratumoral regulatory T cells. Drug Resist Updat. 2022;65:100886. 36370665 10.1016/j.drup.2022.100886

[362] Propper DJ , Balkwill FR . Harnessing cytokines and chemokines for cancer therapy. Nat Rev Clin Oncol. 2022;19(4):237‐253. 34997230 10.1038/s41571-021-00588-9

[363] Spangler JB , Moraga I , Mendoza JL , Garcia KC . Insights into cytokine‐receptor interactions from cytokine engineering. Annu Rev Immunol. 2015;33:139‐167. 25493332 10.1146/annurev-immunol-032713-120211 PMC4445396

[364] Mantovani A , Barajon I , Garlanda C . IL‐1 and IL‐1 regulatory pathways in cancer progression and therapy. Immunol Rev. 2018;281(1):57‐61. 29247996 10.1111/imr.12614 PMC5922413

[365] Hernandez R , Põder J , LaPorte KM , Malek TR . Engineering IL‐2 for immunotherapy of autoimmunity and cancer. Nat Rev Immunol. 2022;22(10):614‐628. 35217787 10.1038/s41577-022-00680-w

[366] Johnson DE , O'Keefe RA , Grandis JR . Targeting the IL‐6/JAK/STAT3 signalling axis in cancer. Nat Rev Clin Oncol. 2018;15(4):234‐248. 29405201 10.1038/nrclinonc.2018.8 PMC5858971

[367] Liu H , Zhao Q , Tan L , et al. Neutralizing IL‐8 potentiates immune checkpoint blockade efficacy for glioma. Cancer Cell. 2023;41(4):693‐710. 36963400 10.1016/j.ccell.2023.03.004

[368] Kurz E , Hirsch CA , Dalton T , et al. Exercise‐induced engagement of the IL‐15/IL‐15Rα axis promotes anti‐tumor immunity in pancreatic cancer. Cancer Cell. 2022;40(7):720‐737. 35660135 10.1016/j.ccell.2022.05.006 PMC9280705

[369] Wang L , Zhu L , Liang C , et al. Targeting N6‐methyladenosine reader YTHDF1 with siRNA boosts antitumor immunity in NASH‐HCC by inhibiting EZH2‐IL‐6 axis. J Hepatol. 2023. 10.1016/j.jhep.2023.06.021 37459919

[370] Wang Z , Cao YJ . Adoptive cell therapy targeting neoantigens: a frontier for cancer research. Front Immunol. 2020;11:176. 32194541 10.3389/fimmu.2020.00176 PMC7066210

[371] Kalbasi A , Siurala M , Su LL , et al. Potentiating adoptive cell therapy using synthetic IL‐9 receptors. Nature. 2022;607(7918):360‐365. 35676488 10.1038/s41586-022-04801-2 PMC9283313

[372] Zhang X , Zhu L , Zhang H , Chen S , Xiao Y . CAR‐T cell therapy in hematological malignancies: current opportunities and challenges. Front Immunol. 2022;13:927153. 35757715 10.3389/fimmu.2022.927153 PMC9226391

[373] Maalej KM , Merhi M , Inchakalody VP , et al. CAR‐cell therapy in the era of solid tumor treatment: current challenges and emerging therapeutic advances. Mol Cancer. 2023;22(1):20. 36717905 10.1186/s12943-023-01723-z PMC9885707

[374] Dolton G , Rius C , Wall A , et al. Targeting of multiple tumor‐associated antigens by individual T cell receptors during successful cancer immunotherapy. Cell. 2023.10.1016/j.cell.2023.06.02037490916

[375] Huang S , Wang X , Wang Y , et al. Deciphering and advancing CAR T‐cell therapy with single‐cell sequencing technologies. Mol Cancer. 2023;22(1):80. 37149643 10.1186/s12943-023-01783-1 PMC10163813

[376] Depil S , Duchateau P , Grupp SA , Mufti G , Poirot L . Off‐the‐shelf’ allogeneic CAR T cells: development and challenges. Nat Rev Drug Discov. 2020;19(3):185‐199. 31900462 10.1038/s41573-019-0051-2

[377] Zhao W , Wu Y , Zhao F , et al. Scoring model based on the signature of non‐m6A‐related neoantigen‐coding lncRNAs assists in immune microenvironment analysis and TCR‐neoantigen pair selection in gliomas. J Transl Med. 2022;20(1):494. 36309750 10.1186/s12967-022-03713-z PMC9617417

[378] Zhang B , Wu Q , Li B , Wang D , Wang L , Zhou YL . m(6)A regulator‐mediated methylation modification patterns and tumor microenvironment infiltration characterization in gastric cancer. Mol Cancer. 2020;19(1):53. 32164750 10.1186/s12943-020-01170-0 PMC7066851

[379] Feng G , Wu Y , Hu Y , et al. Small molecule inhibitors targeting m6A regulators. J Hematol Oncol. 2024;17(1):30. 38711100 10.1186/s13045-024-01546-5 PMC11075261

[380] Cully M . Chemical inhibitors make their RNA epigenetic mark. Nat Rev Drug Discov. 2019;18(12):892‐894. 31780844 10.1038/d41573-019-00179-5

[381] Chen Z , Wu L , Zhou J , et al. N6‐methyladenosine‐induced ERRγ triggers chemoresistance of cancer cells through upregulation of ABCB1 and metabolic reprogramming. Theranostics. 2020;10(8):3382‐3396. 32206097 10.7150/thno.40144 PMC7069076

[382] Wang M , Liu J , Zhao Y , et al. Upregulation of METTL14 mediates the elevation of PERP mRNA N6 adenosine methylation promoting the growth and metastasis of pancreatic cancer. Mol Cancer. 2020;19(1):130. 32843065 10.1186/s12943-020-01249-8 PMC7446161

[383] Yang Z , Zhao F , Gu X , et al. Binding of RNA m6A by IGF2BP3 triggers chemoresistance of HCT8 cells via upregulation of ABCB1. Am J Cancer Res. 2021;11(4):1428‐1445. 33948366 PMC8085870

[384] Zhang X , Su T , Wu Y , et al. N6‐methyladenosine reader YTHDF1 promotes stemness and therapeutic resistance in hepatocellular carcinoma by enhancing NOTCH1 expression. Cancer Res. 2024;84(6):827‐840. 38241695 10.1158/0008-5472.CAN-23-1916

[385] Barbieri I , Tzelepis K , Pandolfini L , et al. Promoter‐bound METTL3 maintains myeloid leukaemia by m(6)A‐dependent translation control. Nature. 2017;552(7683):126‐131. 29186125 10.1038/nature24678 PMC6217924

[386] Yankova E , Blackaby W , Albertella M , et al. Small‐molecule inhibition of METTL3 as a strategy against myeloid leukaemia. Nature. 2021;593(7860):597‐601. 33902106 10.1038/s41586-021-03536-w PMC7613134

[387] Wang L , Yang Q , Zhou Q , et al. METTL3‐m6A‐EGFR‐axis drives lenvatinib resistance in hepatocellular carcinoma. Cancer Lett. 2023;559:216122. 36898427 10.1016/j.canlet.2023.216122

[388] Xu QC , Tien YC , Shi YH , et al. METTL3 promotes intrahepatic cholangiocarcinoma progression by regulating IFIT2 expression in an m(6)A‐YTHDF2‐dependent manner. Oncogene. 2022;41(11):1622‐1633. 35094011 10.1038/s41388-022-02185-1 PMC8913368

[389] Yang Y , Zhang Y , Chen G , et al. KAP1 stabilizes MYCN mRNA and promotes neuroblastoma tumorigenicity by protecting the RNA m(6)A reader YTHDC1 protein degradation. J Exp Clin Cancer Res. 2024;43(1):141. 38745192 10.1186/s13046-024-03040-9 PMC11092262

[390] Pomaville M , Chennakesavalu M , Wang P , et al. Small‐molecule inhibition of the METTL3/METTL14 complex suppresses neuroblastoma tumor growth and promotes differentiation. Cell Rep. 2024;43(5):114165. 38691450 10.1016/j.celrep.2024.114165 PMC11181463

[391] Zhou J , Zhang H , Zhong K , et al. N6‐methyladenosine facilitates mitochondrial fusion of colorectal cancer cells via induction of GSH synthesis and stabilization of OPA1 mRNA. Natl Sci Rev. 2024;11(3):nwae039. 38549713 10.1093/nsr/nwae039 PMC10977914

[392] Chen Y , He Y , Li Z , et al. METTL3 facilitates renal cell carcinoma progression by PLOD2 m(6)A‐methylation under prolonged hypoxia. Cell Death Dis. 2024;15(1):62. 38233403 10.1038/s41419-023-06411-w PMC10794171

[393] Xuan YF , Lu S , Ou YJ , et al. The combination of methionine adenosyltransferase 2A inhibitor and methyltransferase like 3 inhibitor promotes apoptosis of non‐small cell lung cancer cells and produces synergistic anti‐tumor activity. Biochem Biophys Res Commun. 2024;716:150011. 38704890 10.1016/j.bbrc.2024.150011

[394] Xiao H , Zhao R , Meng W , Liao Y . Effects and translatomics characteristics of a small‐molecule inhibitor of METTL3 against non‐small cell lung cancer. J Pharm Anal. 2023;13(6):625‐639. 37440912 10.1016/j.jpha.2023.04.009 PMC10334285

[395] Fraser G , Sorlet C , Parmentier N , et al. EP102: pharmacological inhibition of METTL3 elicits tumor growth inhibition in vivo and demonstrates synergy with venetoclax in various AML models. Blood. 2023;142(1):2263‐2263.

[396] Dolbois A , Bedi RK , Bochenkova E , et al. 1,4,9‐Triazaspiro[5.5]undecan‐2‐one derivatives as potent and selective METTL3 inhibitors. J Med Chem. 2021;64(17):12738‐12760. 34431664 10.1021/acs.jmedchem.1c00773

[397] Huang Y , Su R , Sheng Y , et al. Small‐molecule targeting of oncogenic FTO demethylase in acute myeloid leukemia. Cancer Cell. 2019;35(4):677‐691. 30991027 10.1016/j.ccell.2019.03.006 PMC6812656

[398] Huang Y , Xia W , Dong Z , Yang CG . Chemical inhibitors targeting the oncogenic m(6)A modifying proteins. Acc Chem Res. 2023;56(21):3010‐3022. 37889223 10.1021/acs.accounts.3c00451

[399] Xiao P , Duan Z , Liu Z , et al. Rational design of RNA demethylase FTO inhibitors with enhanced antileukemia drug‐like properties. J Med Chem. 2023;66(14):9731‐9752. 37418628 10.1021/acs.jmedchem.3c00543

[400] Huff S , Tiwari SK , Gonzalez GM , Wang Y , Rana TM . m(6)A‐RNA Demethylase FTO Inhibitors Impair Self‐Renewal in Glioblastoma Stem Cells. ACS Chem Biol. 2021;16(2):324‐333. 33412003 10.1021/acschembio.0c00841 PMC7901021

[401] Huff S , Kummetha IR , Zhang L , et al. Rational design and optimization of m(6)A‐RNA demethylase FTO inhibitors as anticancer agents. J Med Chem. 2022;65(16):10920‐10937. 35939803 10.1021/acs.jmedchem.1c02075 PMC9421652

[402] Su R , Dong L , Li Y , et al. Targeting FTO suppresses cancer stem cell maintenance and immune evasion. Cancer Cell. 2020;38(1):79‐96. 32531268 10.1016/j.ccell.2020.04.017 PMC7363590

[403] Canaani J , Danylesko I , Shemtov N , et al. A phase II study of bisantrene in patients with relapsed/refractory acute myeloid leukemia. Eur J Haematol. 2021;106(2):260‐266. 33159365 10.1111/ejh.13544

